# The U1 snRNP protein U1C and Helix H of U1 snRNA are critical for small molecule splicing modulator function

**DOI:** 10.1038/s41467-026-74765-8

**Published:** 2026-07-16

**Authors:** Zhiling Kuang, Brian Kosmyna, Xueni Li, Zhichao Tang, Shasha Shi, Karoline Lambert, Wenzheng Zhang, Joseph Giovinazzo, Kerstin A. Effenberger, Jairo Sierra, Lanqing Ying, Scott J. Barraza, Wencheng Li, Jingxin Wang, Christopher R. Trotta, Rui Zhao

**Affiliations:** 1 Department of Biochemistry and Molecular Genetics, University of Colorado Anschutz Medical Campus, Aurora, CO USA; 2 PTC Therapeutics Inc., Warren, NJ USA; 3 Section of Genetic Medicine, Department of Medicine, Biological Sciences Division, University of Chicago, Chicago, IL USA; 4 Present Address: College of Plant Protection, Shandong Agricultural University, Tai’an, China; 5 Present Address: Department of Molecular and Cell Biology, School of Life Sciences, China Pharmaceutical University, Nanjing, China

**Keywords:** RNA, RNA splicing

## Abstract

Risdiplam and branaplam represent two classes of small-molecule splicing modulators that promote U1 snRNP recognition of weak non-canonical GA/GU-containing 5’ splice sites (ss). We demonstrate that branaplam enhances recognition of these 5’ ss by reconstituted U1 snRNP in vitro, and that this effect depends on the ZnF domain of U1C and Helix H of U1 snRNA, but not U1A or U1-70K. We also demonstrate that branaplam enhances the weak 5’ ss recognition through a dual act of strengthening the U1 snRNP–5’ ss interaction and U1 snRNP–U1C interaction. In cells, depletion of U1C reduces or abolishes compound-induced exon inclusion for most cassette exons. Interestingly, a subset of cassette exons become responsive to compound only upon U1C knockdown, supporting a model in which U1C stabilizes specific conformations at the 5’ ss–U1 snRNA interface in a context-dependent manner that can either facilitate or hinder compound binding. Surprisingly, risdiplam shows no effect on weak 5’ ss recognition in vitro, suggesting additional cellular factors are required for its activity.

## Abstract (web summary)

How small molecule splicing modulators such as branaplam promote U1 snRNP recognition of weak 5’ splice sites remain incompletely understood. Here the authors combine in vitro and in vivo analyses to reveal key components of U1 snRNP required for branaplam activity and their mechanism of action.

## Introduction

Pre-mRNA splicing is an essential step in the gene expression pathway of all eukaryotes^1,2^. A majority of human genes contain alternatively spliced introns, which serve as a fundamental mechanism of gene regulation^3^. Accurate and efficient recognition of the splice sites is crucial for eukaryotic gene expression. The spliceosome, a huge protein-RNA complex, is responsible for recognizing and splicing introns^4^. During early spliceosome assembly, the 5′ splice site (ss) is recognized by the U1 snRNP, a major component of the spliceosome^5–8^. Human U1 snRNP consists of a 164 nt U1 snRNA, seven Sm proteins that form a heptameric Sm ring, U1A, U1C, and U1–70 K proteins. The recognition of the 5′ ss by U1 snRNP involves base pairing between the 5′ ss and the 5′ end of U1 snRNA. The U1C protein binds and stabilizes this RNA duplex using its Zinc-Finger (ZnF) domain^9,10^. Recently, small molecule splicing modulators that improve the recognition of the weak non-canonical 5′ ss by U1 snRNP have emerged as a unique approach to efficiently modulate protein levels in genetic diseases affecting the CNS, such as Spinal Muscular Atrophy (SMA).

SMA is the leading cause of infant death in the US. It is caused by the mutation or deletion of the *SMN1* gene, resulting in the reduction or absence of SMN proteins and subsequent damage or death of motor neurons^11^. All humans have a paralog of the gene, *SMN2*, which encodes the same protein but contains silent mutations that lead to the skipping of exon 7, which contains a weak non-canonical 5′ ss with the GA/GU sequence (where “/” denotes the exon and intron boundary and nucleotides upstream and downstream of the boundary are numbered as −1 and +1, respectively) instead of the canonical AG/GU sequence^12,13^. The skipping of exon 7 generates a truncated and non-functional protein. Inhibition of this skipping increases the level of full-length SMN protein and rescues disease phenotypes in mouse models^14^. PTC Therapeutics, in collaboration with Roche and the SMA Foundation, identified and developed a class of sequence-specific small molecule splicing modulators represented by risdiplam. These modulators selectively improve the recognition of the non-canonical GA/GU 5′ ss by U1 snRNP^14^. Risdiplam is orally bioavailable with excellent CNS distribution and has been approved for the treatment of SMA by the FDA since 2020.

Risdiplam and related compounds have generated tremendous excitement in the field as small-molecule splicing modulators offer unique approaches to modulating protein levels. One such approach is to promote the inclusion of an exon with a non-canonical 5′ ss by a small molecule in an intron of a pathogenic protein that creates a premature stop codon, leading to reduced protein levels through nonsense-mediated mRNA decay (NMD)^15–17^. This approach is particularly attractive for neurodegenerative diseases such as Huntington’s disease due to the excellent distribution profile of these small molecules in the CNS. Two small-molecule splicing modulators, HTT-C2 and branaplam, target the GA/GU 5′ ss and promote the inclusion of an NMD-inducing exon within intron 49 of the *HTT* pre-mRNA, leading to down-regulation of mutant *HTT* mRNA and protein in mouse models of HD. Optimization of the HTT-C2 class of molecules has led to the advancement of these molecules for treatment of HD in clinical trials^18^.

Although both risdiplam and branaplam enhance the recognition of weak non-canonical GA/GU 5′ ss, they each target a subset of these 5′ ss with preferred upstream and/or downstream sequences. RNAseq analysis of splicing events affected by treatment of cells with either risdiplam or branaplam demonstrated that target 5′ ss were highly enriched for exons with a GA at the −2 and −1 position, with a preference for adenosine at −4 for risdiplam and −3 for branaplan^18^. Massive parallel splicing assay followed by RNAseq analyses further revealed that the risdiplam class of compounds prefers the ANGA/GUHDNN (H = A, C, or U, D = U, A, or G, *N* = any) sequence, while the branaplam class of compounds works on either the above motif or the NAGA/GUNNNN motif^19^. The first motif is present in the 5′ ss of *SMN2* exon 7, and the second motif is present in the 5′ ss of a compound-induced Exon (iExon) within *HTT* intron 49. These added sequence preferences provide impressive target specificity for these compounds. For example, RNA-seq analyses show that branaplam treatment at 24 nM (approximately 2 times the IC50 for *HTT*) for 24 h only affects 165 out of numerous possible splicing events in human SH-SY5Y cells^18^.

The molecular mechanism by which these structurally different compounds improve the recognition of the non-canonical GA/GU sequence with additional upstream and downstream sequence specificity is unclear. An NMR structure of short oligos representing the 5′ ss (11 nt) and U1 snRNA (11 nt) indicates that the 5′ ss and U1 snRNA duplex form a bulge where the non-canonical −1A of the weak 5′ ss flips out, likely preventing efficient binding of the 5′ ss to U1 snRNP^20^. The structure of this short RNA duplex with SMN-C5 (a risdiplam analog) suggests that SMN-C5 brings −1A in and repairs the bulge, strengthening the interaction between the 5′ ss and U1 snRNA^20^. However, the K_d_ between the compound and the short RNA duplex (28 µM) is over 1000-fold higher than the EC50 (15–30 nM) of the compound in cells^20,21^, likely due to the lack of any other components of U1 snRNP in the NMR structure. Furthermore, the structure cannot explain the sequence specificity upstream and downstream of the −1A position, as the critical adenosine at the −4 position was not included in the target RNA. In this study, we used biochemical approaches with purified native U1 snRNP and reconstituted U1 snRNP, combined with in vivo assessments of splicing activity, to understand the mechanism of these small-molecule splicing modulators, which is critical for developing additional compounds into therapeutics for other splicing-related diseases.

## Results

### Branaplam improves the binding of U1 snRNP to weak 5′ ss in vitro

**Fig. 1:**
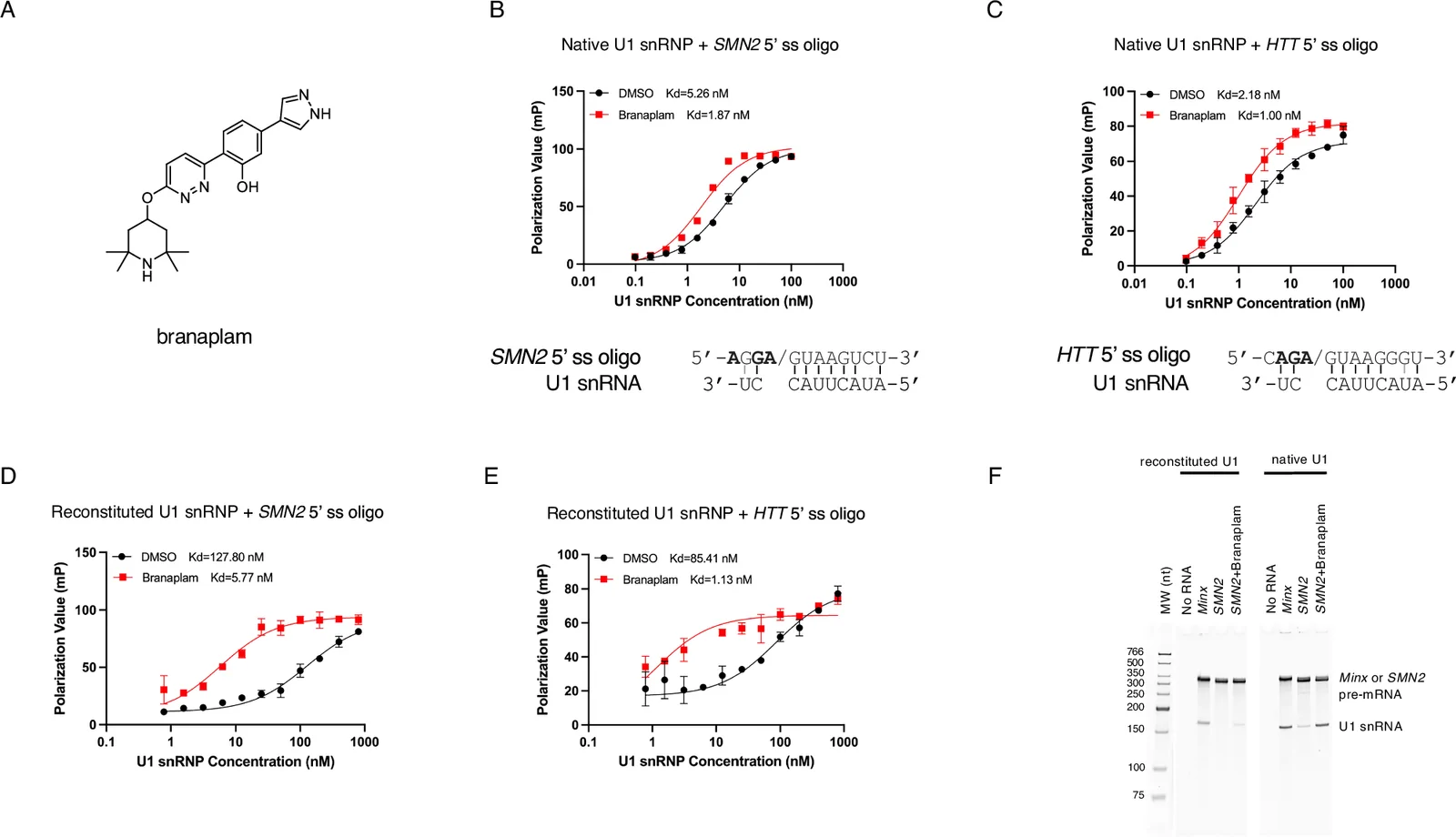
Branaplam improves the binding of native or reconstituted U1 snRNP to the 5′ ss of both *SMN2* and *HTT.* **A** Structure of branaplam. **B** Branaplam improves the binding affinity between native U1 snRNP and the *SMN2* 5′ ss oligo in fluorescence polarization (FP) experiment. In all FP experiments described in this paper, the 5′ ss oligo was fluorescently labeled, and increasing concentrations of U1 snRNP was added to the binding reaction in the presence of DMSO, or 10 μM branaplam dissolved in the same concentration of DMSO, unless otherwise stated. All data in this figure are presented as mean +/− SD from two independent biological replicates. **C** Branaplam improves the binding affinity between native U1 snRNP and the *HTT* 5′ ss oligo in FP experiment. **D** Branaplam improves the binding affinity between reconstituted U1 snRNP and the *SMN2* 5′ ss oligo in FP experiment. **E** Branaplam improves the binding affinity between reconstituted U1 snRNP and the *HTT* 5′ ss oligo in FP experiment. **F** MBP bound MS2 tagged *Minx* (as a positive control with the canonical 5′ ss) or *SMN2* pre-mRNA containing the weak 5′ ss of exon 7 was immobilized on amylose resin, incubated with either native or reconstituted U1 snRNP in the absence or presence of branaplam. RNAs from the eluted samples were analyzed on a denaturing gel with SybrGold staining. The image shown is representative of two independent biological replicates.

To evaluate the effect of branaplam on U1 snRNP’s ability to bind weak 5′ ss, we used a fluorescence polarization (FP) experiment with a fluorescently labeled RNA oligo containing sequence around the 5′ ss of exon 7 in the *SMN2* pre-mRNA (AGGA/GUAAGUCU, where/indicates the exon and intron boundary, designated as *SMN2* 5′ ss oligo) or around the 5′ ss of an iExon within intron 49 in the *HTT* pre-mRNA (CAGA/GUAAGGGU, designated as *HTT* 5′ ss). We engineered a protein A-tag on U1A in HeLa cells using CRISPR-Cas9 and purified native U1 snRNP using the protein A tag and IgG resin (Supplementary Fig. 1A). We titrated increasing concentrations of purified native U1 snRNP and determined the K_d_ between U1 snRNP and these weak 5′ ss in the presence of DMSO or branaplam. Since U1C is weakly associated with U1 snRNP and may fall off at low U1 snRNP concentrations^22^, we added a large excess of full-length U1C (800 nM) expressed in and purified from *E. coli* in all binding reactions unless otherwise stated. Using this experiment, we found that branaplam increased the binding affinity between native U1 snRNP and the *SMN2* or *HTT* 5′ ss oligo by about twofold (Fig. 1A–C). Although the affinity difference is small, it is highly reproducible.

To evaluate the effect of branaplam on reconstituted U1 snRNP, protein components were expressed in and purified from *E. coli*, using constructs provided by the Allain group, essentially following their published protocol^23^. Among these, the SmB construct contains residues 1–174 (the full length contains 240 residues) and the U1–70 K construct contains residues 1–216 (the C-terminal RS domain was truncated). The other subunits are all full-length. We reconstituted the U1 snRNP using these protein components and full-length U1 snRNA and purified the reconstituted U1 snRNP by gel filtration (Supplementary Fig. 1B). Similar to the previous result when using purified native U1 snRNP, branaplam dramatically increased the binding affinity between the reconstituted U1 snRNP and the *SMN2* or *HTT* 5′ ss oligo (Fig. 1D, E).

As an orthogonal method, we used pulldown assays to evaluate the effect of branaplam on U1 snRNP and 5′ ss binding. In this assay, we immobilized *Minx* (as a positive control with a canonical 5′ ss) or *SMN2* pre-mRNA containing the weak 5′ ss of exon 7 on amylose resin, pulled down either native or reconstituted U1 snRNP, and analyzed the RNAs in the pulldown samples as a surrogate of the amount of U1 snRNP bound to the pre-mRNA. We found that branaplam significantly increased the amount of native or reconstituted U1 snRNPs pulled down by the *SMN2* pre-mRNA (Fig. 1F). We also found that the *SMN2* pre-mRNA pulled down more native than reconstituted U1 snRNP under all conditions, suggesting that the native U1 snRNP binds the *SMN2* pre-mRNA better than the reconstituted U1 snRNP, consistent with our observations in FP experiments (Fig. 1B–E).

### Risdiplam does not improve the binding of U1 snRNP to weak 5′ ss in vitro

**Fig. 2:**
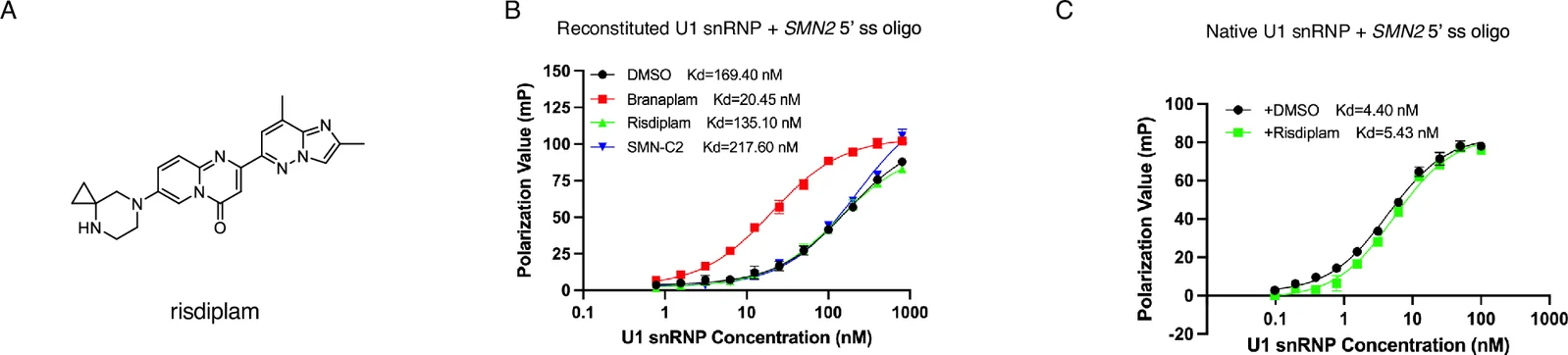
Risdiplam does not improve the binding of U1 snRNP to weak 5′ ss in vitro. **A** Structure of risdiplam. **B** Branaplam, but not risdiplam or its SMN-C2 analog, improves the binding of reconstituted U1 snRNP to the *SMN2* 5′ ss oligo in FP experiments. All data in this figure are presented as mean +/− SD from two independent biological replicates. **C** Risdiplam has no effect on the binding of native U1 snRNP to the *SMN2* 5′ ss oligo in FP experiments.

We next evaluated the effect of risdiplam on the binding between U1 snRNP and weak 5′ ss containing the GA/GU motif. To our surprise, risdiplam (or its analogs such as SMN-C2) had no effect on the binding between either native or reconstituted U1 snRNP and the *SMN2* 5′ ss oligo (or the FOXM1 5′ ss oligo with the sequence of **A**U**GA/**GUAAGUUC) (Fig. 2A–C and Supplementary Fig. 2). Due to this limitation, all our subsequent in vitro experiments were performed using branaplam and analogs.

### Branaplam analogs have remarkably similar in vitro and in vivo activity profiles

**Fig. 3:**
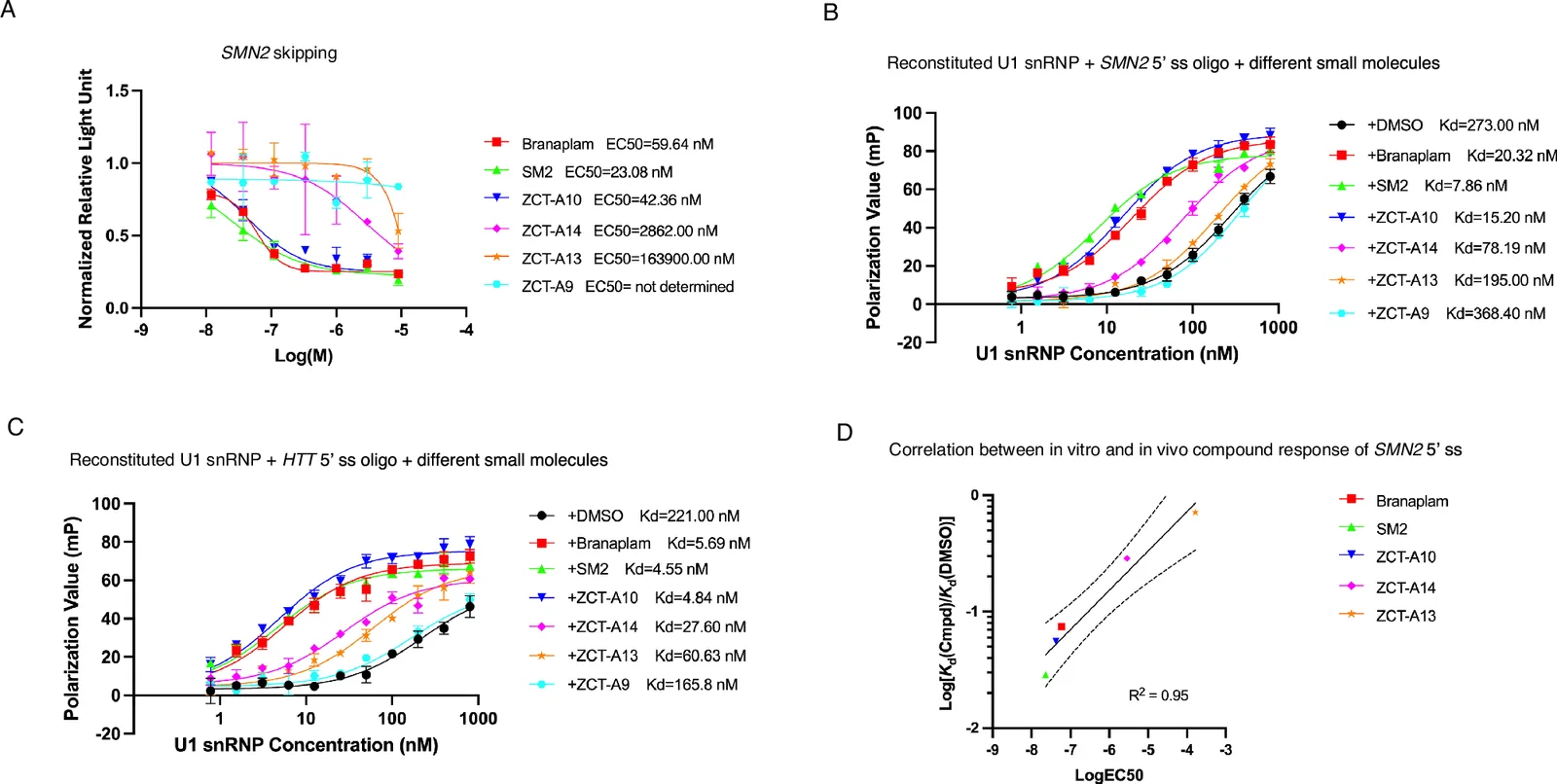
Various branaplam analogs have similar activity profiles in inducing *SMN2* exon 7 inclusion in cells and in binding to the *SMN2* or *HTT* 5′ ss oligos in vitro. **A** A *SMN2* exon 7 reporter plasmid was transformed into HEK293T cells, treated with increasing concentrations of branaplam analogs, and the extent of *SMN2* exon 7 skipping was evaluated using a luciferase reporter (skipping of exon 7 leads to higher luciferase signal, see “Methods” for details) and quantified. All data in this figure are presented as mean +/− SD from two independent biological replicates. **B** FP experiments were carried out using fluorescently labeled *SMN2* 5′ ss oligo, increasing concentrations of reconstituted U1 snRNP, in the presence of DMSO or 10 μM of branaplam analogs. **C** FP experiments were carried out using fluorescently labeled *HTT* 5′ ss oligo, increasing concentrations of reconstituted U1 snRNP, in the presence of DMSO or 10 μM of branaplam analogs. **D** Scatterplot of log(EC50) values from the reporter assay versus log[K_d_(compound)/K_d_(DMSO)] from the in vitro binding assay using *SMN2* 5′ ss, showing a strong correlation between cellular potency and direct binding affinity in vitro. The solid line denotes the best-fit linear regression (*R*² = 0.95), and the dashed lines represent the 95% confidence interval.

To assess how our in vitro binding assay correlates with the cellular activity of branaplam analogs, we synthesized a series of branaplam analogs with various substituents on the pyridazine core (Supplementary Fig. 3). We evaluated the cellular effect of these analogs on inducing *SMN2* exon 7 inclusion using a splicing reporter assay in HEK293 cells and showed that they had various levels of activity with the order: branaplam ~ SM2 ~ ZCT-A10 > ZCT-A14 > ZCT-A13 > ZCT-A9 (Fig. 3A). We next evaluated the effect of these analogs on the binding affinity between U1 snRNP and the *SMN2* or *HTT* 5′ ss oligo in FP experiments (Fig. 3B, C). The order of the in vitro effects of these analogs was remarkably consistent with their in vivo cellular activities in the splicing reporter assay (Fig. 3D).

### Mutations of critical nucleotides near the 5′ ss abolish the effect of branaplam

**Fig. 4:**
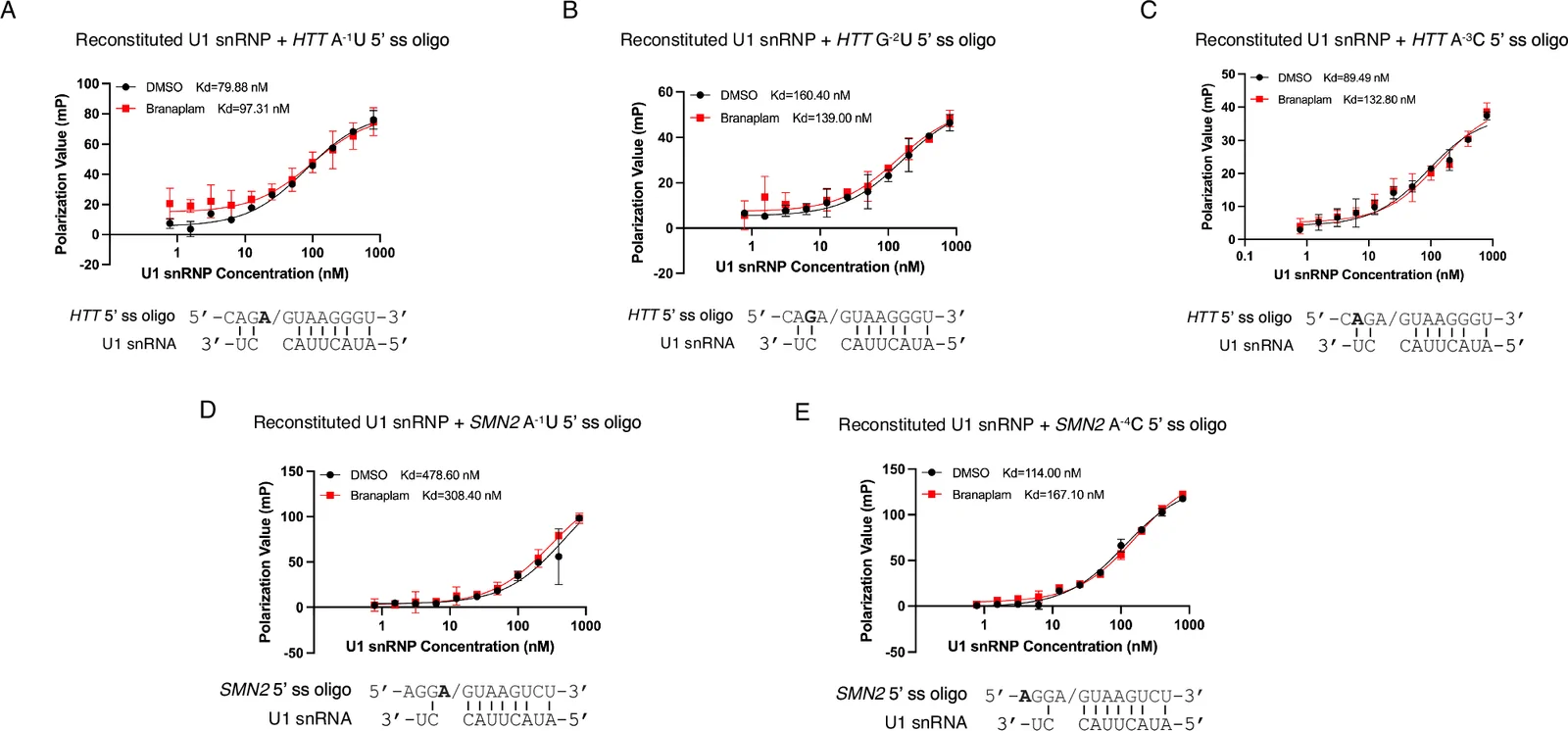
Mutations of critical nucleotides in the *SMN2* and *HTT* 5′ ss oligos abolish the effect of branaplam on 5′ ss binding. FP experiments were performed using fluorescently labeled *HTT* (**A**–**C**) or *SMN2* (**D**, **E**) oligo carrying the indicated mutant and increasing concentrations of reconstituted U1 snRNP in the presence of DMSO or branaplam. The nucleotide mutated is shown in bold in the U1 snRNA and 5′ ss duplex sequence below each panel. All data in this figure are presented as mean +/− SD from two independent biological replicates.

Massive parallel splicing assays (MPSA) revealed that ANGA/GUHDNN and NAGA/GUNNNN are two 5′ ss motifs responsive to splicing modulators^19^. The first motif is reflected in the *SMN2* 5′ ss (**A**G**GA**/GUAAGUCU, referred to as the −4A motif) and the second motif is reflected in the *HTT* 5′ ss (C**AGA**/GUAAGGGU, referred to as the −3A motif). RNAseq analysis of exons included upon treatment with splicing modulators also showed that a distinct specificity for the −3A motif with branaplam and −4A motif with risdiplam^18^. We mutated nucleotides at several key positions in these motifs and evaluated their effect on U1 snRNP recognition of these 5′ ss in the presence of branaplam. For the *HTT* oligo, A^−1^U (nucleotide A at −1 position mutated to U), G^−2^U, and A^−3^C mutants all abolished the effect of branaplam (Fig. 4A–C and Supplementary Fig. 4). We chose the mutant nucleotide to be different from that in the motif but avoided the canonical 5′ ss nucleotides at these positions (A at −2 and G at −1 positions). For the *SMN2* oligo, the A^−1^U and A^−4^C mutants also abolished the effect of branaplam (Fig. 4D, E and Supplementary Fig. 4). These results are all consistent with motifs revealed by RNAseq and MPSA analyses, supporting that the in vitro effect of branaplam on reconstituted U1 snRNP and 5′ ss binding recapitulates its effect in cells, thus it is likely that direct compound interaction with U1 snRNP and 5′ ss RNA is the primary contributor to the in vivo sequence specificity^18,19^.

### U1C but not U1A or U1–70 K is important for the effect of branaplam on 5′ ss binding in vitro

**Fig. 5:**
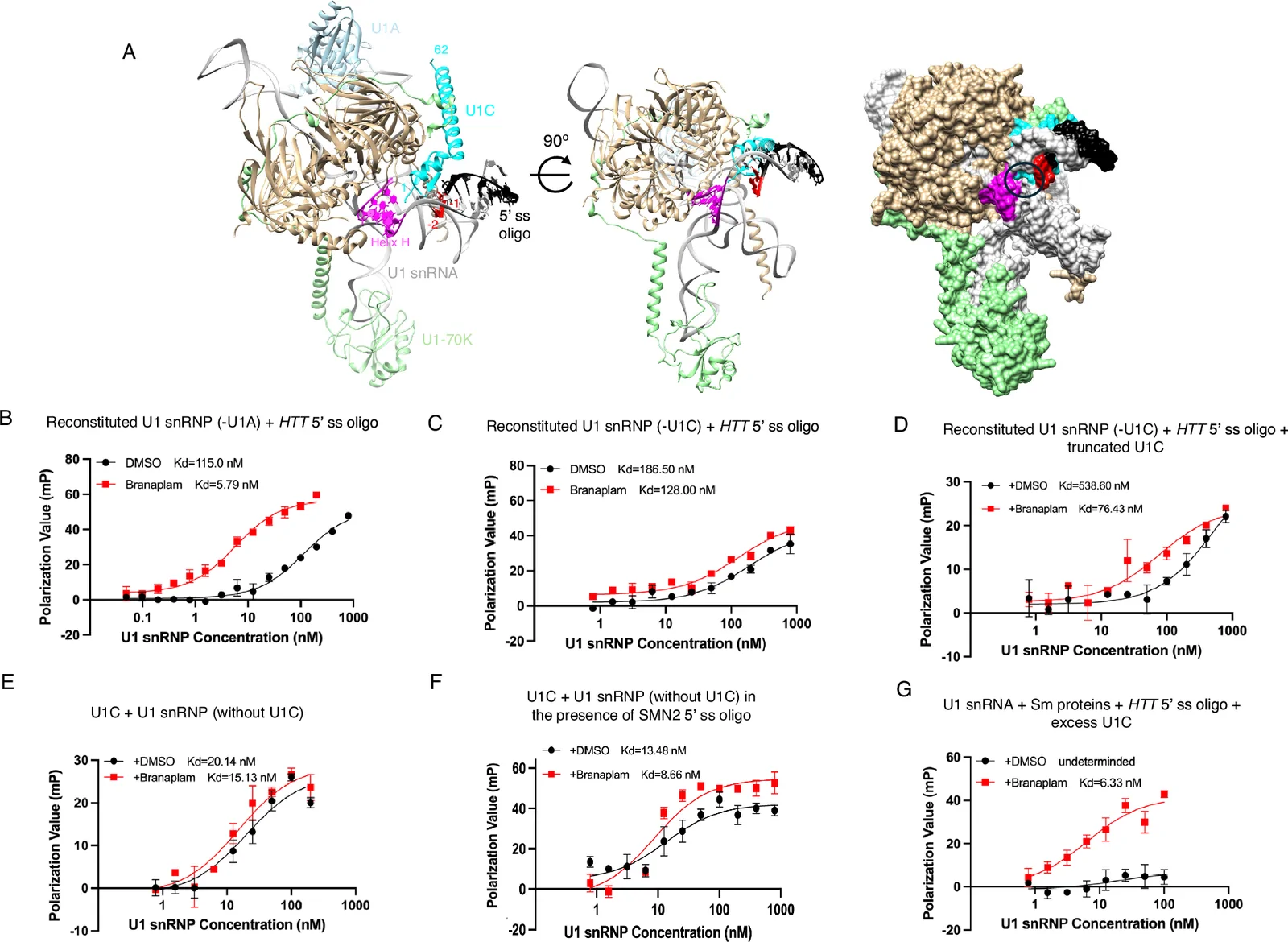
U1C, but not U1A or U1–70 K, is important for the effect of branaplam on 5′ ss binding in vitro. **A** Left panel: a model of reconstituted U1 snRNP based on previous low-resolution or partial structures of human U1 snRNPs ^9,10,37^. Middle panel: a view that is rotated 90° along the horizontal axis from the left view. Right panel: the surface representation of the middle panel with a potential cavity near the 5′ ss highlighted in black circle. Black is the 5′ ss oligo used in the crystal structure of a minimal U1 snRNP that fully complement the 5′ end of U1 snRNA^10^. −1 and −2 positions (relative to the 5′ ss) of the oligo are indicated in red. Purple represent Helix H on the U1 snRNA. Numbers on U1C indicate residue numbers. **B** U1 snRNP was reconstituted without U1A and used for FP experiments with fluorescently labeled *HTT* oligo in the presence of DMSO or branaplam. All data in this figure are presented as mean +/− SD from two independent biological replicates. **C** U1 snRNP was reconstituted without U1C and used for FP experiments with fluorescently labeled *HTT* oligo in the presence of DMSO or branaplam. **D** U1 snRNP was reconstituted without U1C and used for FP experiments with fluorescently labeled *HTT* oligo in the presence of DMSO or branaplam as well as excess truncated U1C (residues 1–61). **E** Increasing concentrations of U1 snRNP without U1C was added to fluorescently labeled U1C in FP experiment. **F** Increasing concentrations of U1 snRNP without U1C was added to fluorescently labeled U1C in the presence of SMN2 5′ ss oligo in FP experiment. **G** U1 snRNP was reconstituted with U1 snRNA, Sm proteins, and 373 μM of U1C and used for FP experiments with fluorescently labeled *HTT* oligo in the presence of DMSO or branaplam.

We next took advantage of the modular nature of reconstituted U1 snRNP to first decipher the protein components required for branaplam’s effect on U1 snRNP and weak 5′ ss binding. U1 snRNP contains seven Sm proteins that are shared with other snRNPs and three U1 snRNP-specific proteins, U1A, U1C, and U1–70 K (Fig. 5A). We evaluated the role of the three U1-specific proteins in the effect of branaplam. We first reconstituted U1 snRNP without U1A or U1C. We found that branaplam still dramatically improved the binding of U1 snRNP to the *HTT* 5′ ss oligo in the U1A-depleted U1 snRNP but no longer did so for U1 snRNP without U1C (Fig. 5B, C). The addition of a truncated U1C (residues 1–61) that contains the ZnF domain without the disordered C-terminal domain (Fig. 5A) restored the effect of branaplam (Fig. 5D). These data together demonstrate that the ZnF domain of U1C, but not U1A protein, is important for the effect of branaplam on 5′ ss binding in vitro.

We then evaluated the effect of the compound on the interaction between U1C and the rest of U1 snRNP. We labeled the N-terminus of U1C with a Cy5 fluorophore and carried out FP experiment with labeled U1C and increasing concentrations of U1 snRNP without U1C. In the absence of a 5′ ss oligo, branaplam did not change the binding affinity between U1C and the rest of U1 snRNP (Fig. 5E). Intriguingly, branaplam improved the binding affinity between U1C and the rest of U1 snRNP in the presence of the *SMN2* 5′ ss oligo, with a small but highly reproducible decrease in K_d_ (Fig. 5F). We used the *SMN2* 5′ ss oligo because we had unlabeled *SMN2* but not unlabeled *HTT* oligo and because the two behaved similarly in all FP experiments (Figs. 1 and 3).

It is more difficult to evaluate the effect of U1–70 K depletion on branaplam activity because the N-terminus of U1–70 K interacts with U1C and the removal of U1–70 K will also remove U1C from U1 snRNP (Fig. 5A). To overcome this problem, we assembled the U1 snRNA with Sm proteins, then added large excess of U1C protein (373 μM). We showed that branaplam was able to improve the binding of this reconstituted U1 snRNP (without U1A and U1–70 K) to the *HTT* oligo (Fig. 5G), suggesting that U1–70 K is not required for the effect of branaplam.

### U1 snRNA and 5′ ss duplex with the A bulge is not sufficient for the effect of branaplam

It was speculated that the −1A bulge formed between the 5′ end of U1 snRNA and the weak 5′ ss containing GA/GU is critical for the effect of risdiplam analogs (and potentially branaplam)^23^. We found that the 5′ end of U1 snRNA (nucleotide 1–10) or the entire U1 snRNA had no substantial binding to the *HTT* 5′ ss oligo (−4 to +8 around the 5′ ss), even in the presence of U1C (Supplementary Fig. 5A–C). We suspected, in this in vitro system (containing only the 5′ ss oligo, U1 snRNA, and U1C), the bulge A at the −1 position was not formed in the U1 snRNA and 5′ ss RNA duplex, potentially due to nucleotides 9 and 10 of U1 snRNA did not form stable basepairs with nucleotides −2G and −3A of the *HTT* 5′ ss oligo. Indeed, when we deleted the −1A nucleotide to allow continuous base pairing between the *HTT* 5′ ss oligo and the U1 snRNA, there was clear binding between the *HTT* 5′ ss oligo and the U1 snRNA (either the 5′ end or the entire U1 snRNA) (Supplementary Fig. 5D, E). As expected, this binding was not improved by branaplam (Supplementary Fig. 5D, E), since there was no −1A bulge. To ensure the presence of a −1A bulge in the RNA duplex, we extended the U1 snRNA to nucleotide 1–16 and generated an artificial *HTT* 5′ ss oligo that added 4 additional nucleotides upstream of the −4C nucleotide to ensure stable base pairing upstream of the potential −1A bulge. U1 snRNA bound to this artificial *HTT* 5′ ss oligo, but branaplam had no effect on the binding affinity (Supplementary Fig. 5F). These results indicate that the −1A bulge in the U1 snRNA and 5′ ss duplex alone without any U1 snRNP protein component is not sufficient for the effect of branaplam, consistent with our observation that U1C is required for the effect of branaplam (Fig. 5).

### Helix H of U1 snRNP is important for the effect of branaplam

**Fig. 6:**
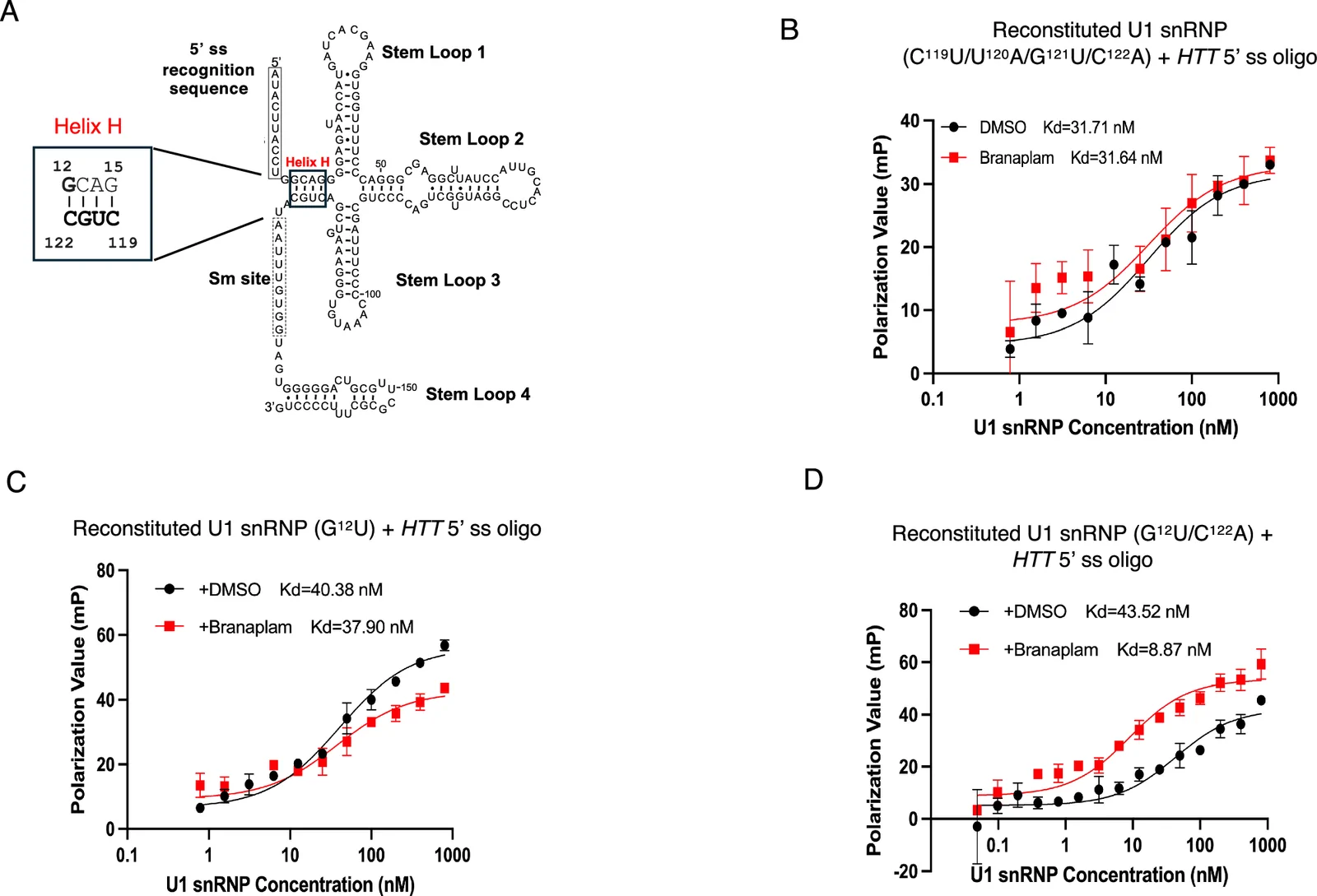
Helix H of U1 snRNA is important for the effect of branaplam on 5′ ss binding in vitro. **A** Secondary structure of U1 snRNA indicating the position of Helix H. An enlarged view of helix H with nucleotide numbers and mutated nucleotides in bold is also shown. **B** U1 snRNP was reconstituted using a U1 snRNA carrying a tetra-mutant that disrupts Helix H and used in FP experiments. All data in this figure are presented as mean +/− SD from two independent biological replicates. **C** U1 snRNP was reconstituted using a U1 snRNA carrying the G^12^U mutant that disrupted the first basepair of Helix H and used in FP experiments. **D** U1 snRNP was reconstituted using a U1 snRNA carrying a double mutant G^12^U/C^122^A that restored Helix H and used in FP experiments.

We next evaluated the role of U1 snRNA, specifically Helix H (Fig. 6A), in the effect of branaplam. To this end, we generated a U1 snRNA tetra mutant (C^119^U/U^120^A/G^121^U/C^122^A) that completely disrupted Helix H. U1 snRNP reconstituted with this snRNA assembled equally well as the WT (Supplementary Fig. 1C) and bound the *HTT* 5′ ss oligo, but this binding was no longer improved by the presence of branaplam (Fig. 6B). We next generated a single G^12^U mutant in Helix H and showed that this mutant also abolished the effect of branaplam on improving the binding between U1 snRNP and *HTT* 5′ ss (Fig. 6C). Furthermore, when we generated a compensatory mutant to restore Helix H (G^12^U/C^122^A), the effect of branaplam on 5′ ss binding was restored (Fig. 6D), indicating the presence of Helix H in U1 snRNA is important for the effect of branaplam. Inspecting the surface representation of the structural model of U1 snRNP + 5′ ss indicated that there is indeed room to potentially accommodate a small molecule in the vicinity of Helix H of U1 snRNA, 5′ ss, and U1C (Fig. 5A).

### Helix H is critical for the recognition of 5′ ss in vivo

**Fig. 7:**
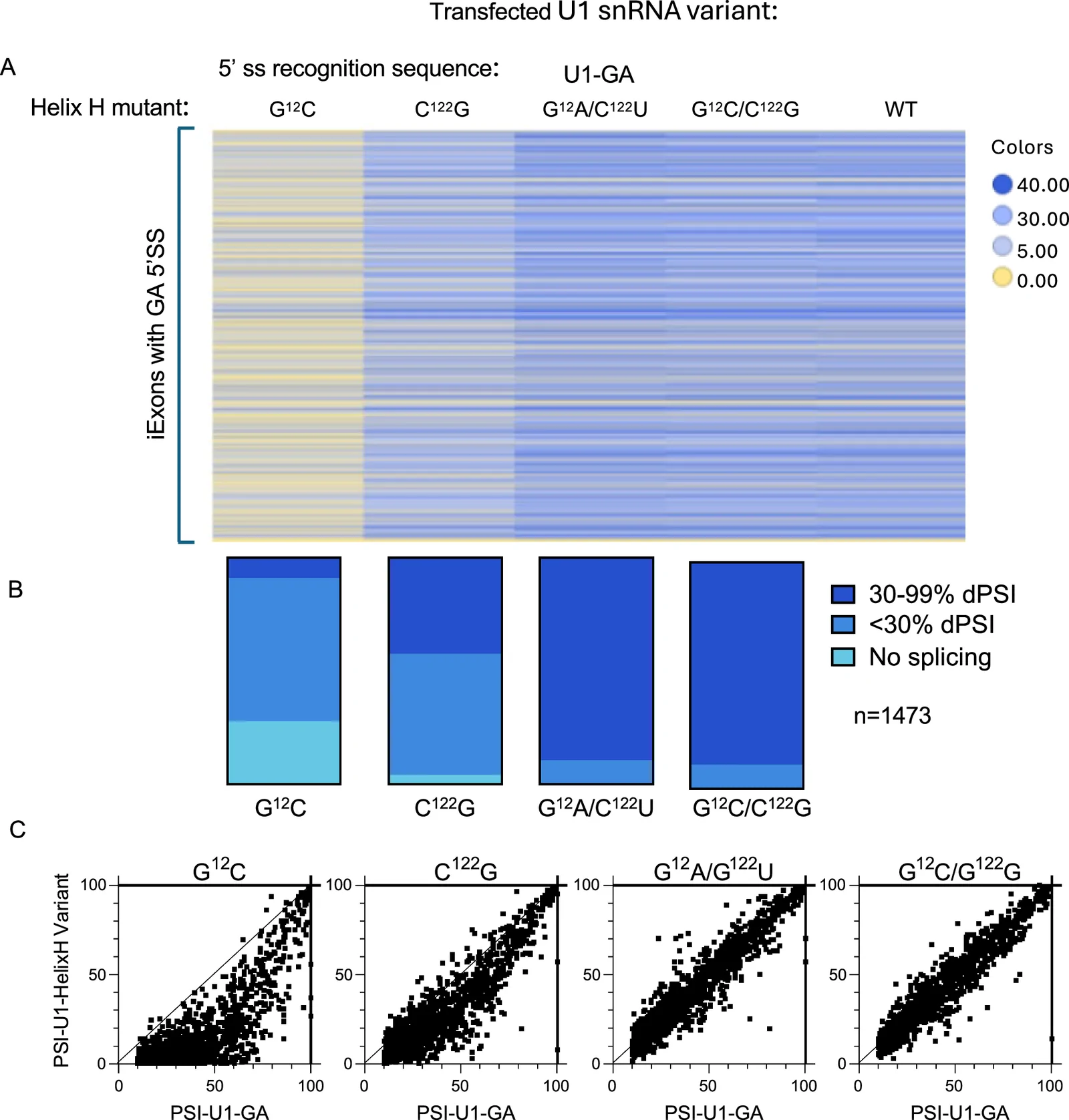
Helix H structure and nucleotide identity are critical to U1 snRNA function in vivo. U1-GA variants each carrying mutations in Helix H were expressed in HEK293 cells and then RNAseq was utilized to determine the level of inclusion of exons containing a GA/GU 5′ ss. **A** Heat map of 1473 iExons induced by U1-GA WT and helix H mutants, colored by delta PSI between the U1-GA helix H variant and mock transfection with larger delta PSI values shown in darker blue and delta PSI 0 in yellow. The heatmap indicates that U1-GA WT induced the inclusion of many iExons, the G^12^C and C^122^G mutants reduced these inclusions, and the G^12^A/C^122^U and G^12^C/C^122^G mutants largely restored the inclusions. **B** Comparison of iExon inclusion between U1-GA WT and U1-GA helix H mutants as indicated by the percent decrease in inclusion of iExons (PSI_mutant_/PSI_WT_ × 100) in three different ranges: light blue indicates iExon does not splice in U1-GA helix H variant, medium blue indicates a ratio of ≤30%, and dark blue indicates a ratio of >30%. **C** Plot of PSI for all GA/GU exons induced by U1-GA variants. PSI for U1-GA with WT helix H is on the *x*-axis and PSI for U1-GA carrying each of the four Helix H mutations is on the *y*-axis. **B** and **C** both indicate that the G^12^C and C^122^G mutants reduced the inclusions induced by U1-GA WT, and the G^12^A/C^122^U and G^12^C/C^122^G mutants largely restored the inclusions. Line drawn is x = y.

To understand the importance of Helix H to splicing in vivo, we utilized a U1 variant that has been engineered to recognize the non-canonical GA/GU 5′ ss by altering U1 snRNA sequence at position 9 and 10 from CU to UC, complementary to −2 and −1 at the non-canonical 5′ ss (designated as U1-GA variant, Supplementary Fig. 6A). Previous studies demonstrated that the U1-GA snRNA variant is incorporated into U1 snRNP and elicits a set of iExons containing GA/GU 5′ ss, whose splicing is dependent on the variant U1 snRNA^18^. When the U1-GA variant is combined with Helix H mutations (Supplementary Fig. 6B), we can now measure the in vivo effect of mutations to U1 Helix H on splicing of the set of exons with a matched 5′ ss that are activated by the U1-GA variant. After expression of either U1-GA variant with wild-type Helix H or U1-GA variant with mutations to key nucleotides in Helix H, we performed RNA sequencing and monitored the inclusion of exons with GA/GU 5′ ss sequences by calculating the deltaPSI (between cells transfected with U1 variants and mock transfected) and determined the effect of mutations to Helix H on exons that are statistically significantly induced with a delta PSI > 10. Our results showed that mutation of the G12 residue at the base of Helix H negatively impacted the ability of the U1-GA variant to function in splicing, resulting in loss or diminished splicing of >90% of GA/GU iExons (Fig. 7A–C and Supplementary Data 1). Additionally, mutation of C122 (also at the base of Helix H) resulted in loss or diminished splicing of ~40% of iExons (Fig. 7A–C). The fact that the G^12^C mutant has a much more pronounced effect than C^122^G suggest that the nucleotide identity in Helix H mutants influences which U1-GA induced iExons are most sensitive to Helix H mutations. Multiple compensatory mutations that restore the base pairing (G^12^A/C^122^U or G^12^C/C^122^G), but alter the identity of the nucleotides, restored the majority of the splicing activity of GA/GU iExons in vivo (Fig. 7A–C and Supplementary Data 1).

We engineered two additional U1 variants to elicit a distinct set of 5′ ss-matched exons containing either non-canonical UU or UG 5′ ss (designated as U1-UU and U1-UG). The effect of Helix H mutations in these additional U1 variants was remarkably consistent with the U1-GA (Supplementary Fig. 6C–E). Together, the in vivo data showing a critical role for Helix H in splicing and our in vitro assay showing the effect of Helix H mutations on the ability of the compound to stabilize the interaction between U1 snRNP and noncanonical *HTT* 5′ ss, support that Helix H plays a critical role in the ability of the compound to induce noncanonical GA 5′ ss recognition and usage.

### U1C modifies activities of branaplam and risdiplam in vivo

To substantiate the role of U1C in compound-mediated splicing in cells, we knocked down (KD) U1C by siRNA in HEK293 cells with scrambled (scr) siRNA serving as a mock knockdown and negative control (Supplementary Fig. 7) and treated cells with either risdiplam or branaplam in different doses for 24 h. Knockdown of U1C alone had a substantial effect on splicing, leading to skipping of 8364 cassette exons throughout the genome independent of the sequence at −2 and −1 positions (Supplementary Fig. 8A, B and Supplementary Data 2), consistent with previous published studies of U1C^24^. Skipped exons are highly enriched for noncanonical 5′ ss sequences (on either the intronic or exonic side), which account for 90% of skipped exons (Supplementary Fig. 8C). For example, among skipped exons with a canonical exonic 5′ ss sequence, 74% contain noncanonical intron sequence. These observations are consistent with the previous published role for U1C stabilization of the U1 snRNA-pre-mRNA duplex at the 5′ ss^25–28^. Interestingly, U1C knockdown also led to the inclusion of 782 cassette exons (Supplementary Fig. 8A, B), suggesting that in addition to its role in stabilization of 5′ ss, U1C also plays a role in suppression of splicing for a subset of exons. We will refer to these as class I exons for clarity in later discussion.

**Fig. 8:**
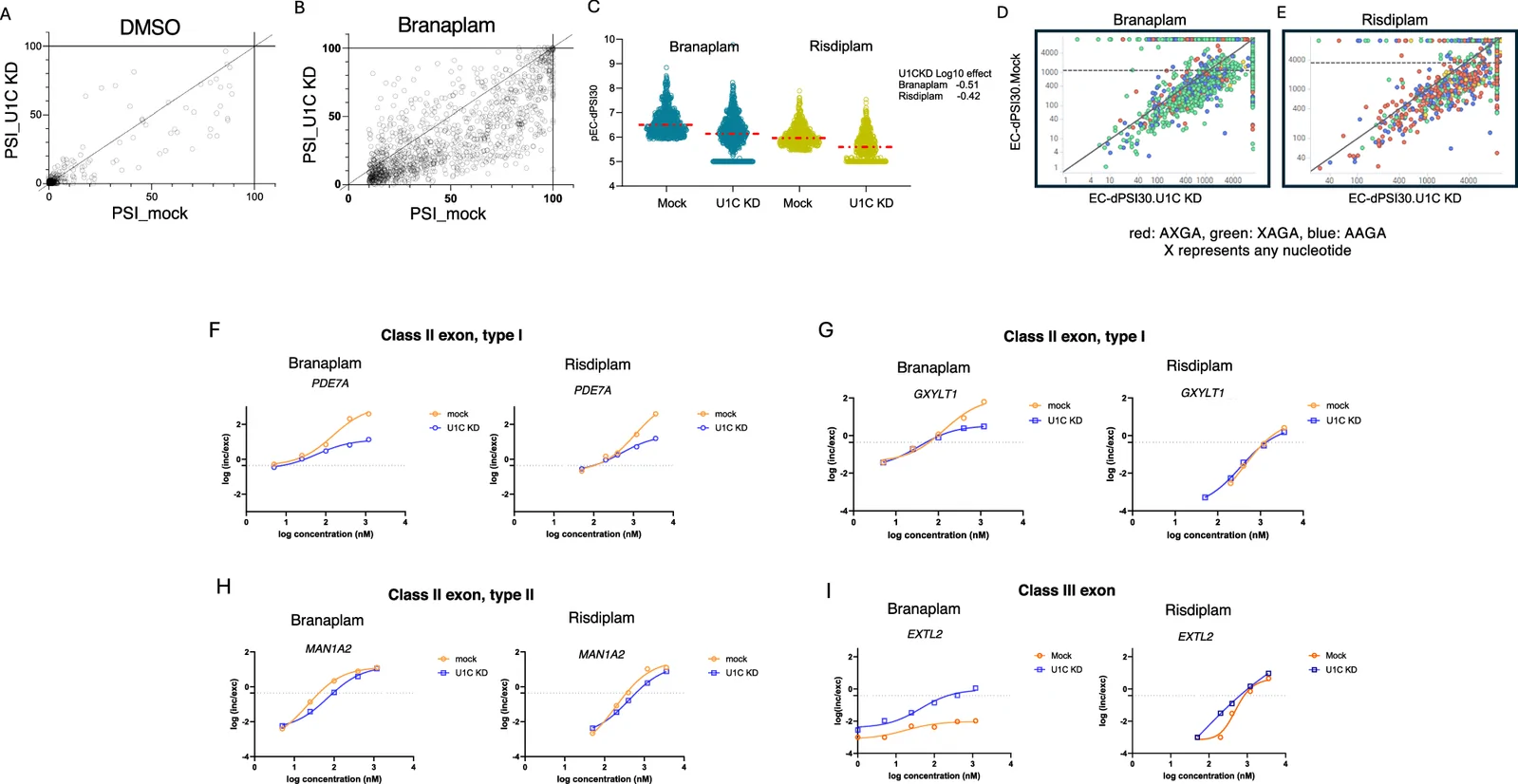
U1C knockdown modifies splicing modulator activity. **A** Scatter plot of PSI of branaplam-induced GA/GU iExons for either scramble siRNA-transfected (*x*-axis) or U1C KD (*y*-axis) cells treated with DMSO. Events where splicing is attenuated by U1C reduction are shifted to the right of the no effect line (y = x) and those that are enhanced shift to the left of the line. Most exons are <10 PSI. **B** Same as (**A**), except that the samples were treated with 1.2 μM branaplam. **C** Plot of pEC_dPSI30 (−log10 transformed compound concentration when dPSI achieves 30%) for all GA/GU iExons. The red dashed lines represent the geomean of each treatment with branaplam mock = 6.61, U1C KD = 6.10; risdiplam mock = 6.05, U1C KD = 5.63. The log10 effect is calculated by subtracting the pEC-dPSI30 of mock versus U1C KD and shows a 0.5 and 0.4 log10 fold decrease for branaplam and risdiplam, respectively. **D** Scatter plot of the EC-dPSI30 for siRNA scramble control (*y*-axis) versus U1C KD (*x*-axis) in branaplam-treated cells. Events where compound potency is attenuated by U1C reduction are shifted to the right of the no effect line (y = x) and those that are enhanced shift to the left of the no effect line. Events that are inactive for either treatment are seen along the top and right side of the plot (no calculable EC-dPSI30). Red, green, and blue dots represent different sequences at the exonic part of the activated exons. **E** Same as (**D**), expect that cells were treated with risdiplam. **F**–**H** Class II exons. Plot of Log10(inclusion/exclusion) of representative exons induced by compound treatment for response type I (**F** and **G**) and response type II (**H**). The inclusion of these exons by compound is reduced by U1C knockdown (except GXYLT1 with risdiplam). Dash lines in (**F**–**I**) represent PSI30. All logarithms in this figure are to base 10. **I** Class III exons. Plot of PSI of a representative exon which has low inclusion in the mock knockdown, but its branaplam-induced inclusion is greatly enhanced by U1C knockdown.

We next examined the effect of U1C knockdown on compound-mediated splicing. Of the approximately 1200 exons with GA/GU 5′ ss that were statistically significantly induced with a deltaPSI_compound-DMSO_ of >10 at the highest concentration of branaplam, approximately 1100 had basal splicing levels in the DMSO of <7.5 PSI (Fig. 8A and Supplementary Fig. 9A). Treatment with 1.2 μM branaplam for 24 h induced exon inclusion ranging from 10 to 100 PSI, however the increased inclusion upon treatment was less when U1C was knocked down (Fig. 8B and Supplementary Fig. 9A). We next used the complete dose response to calculate the effective concentration at which deltaPSI_compound-DMSO_ is 30 (EC-dPSI30). We show that a reduction in U1C led to an overall 0.5 log decrease in the potency for the majority of iExon inclusion events elicited by branaplam (Fig. 8C, D). The same observations can be made for risdiplam (Fig. 8C, E).

The previously observed sequence preference for A at −3 or −4 position from the splice site for branaplam and risdiplam^18^ was not altered by U1C knock down (Supplementary Fig. 9B, C). The effect of U1C knock down did not change the rank order of potencies for compound effect on GA/GU target exons (Supplementary Data 2). For example, the top five iExons most responsive to the compounds remained the most potent events with the same rank order when U1C was knocked down, indicating that U1C KD decreased compound potency across these iExons by a similar magnitude without changing the potency rank order. This suggests that some of the effect on potency could be due to a general negative effect on splicing by U1C knockdown. In addition to the global effect potentially caused by U1C reduction, there was a target specific effect on potency as demonstrated by a wide range of effects for compound induced splicing. These observations suggest that, for many splicing modulator target exons, the exon response to the compound upon reduction of U1C is differentially influenced by U1C availability, in addition to a global effect of U1C reduction on splicing. In particular, significant reduction or complete loss of compound response upon U1C knockdown was observed for a substantial set of exons (Fig. 8C), indicating a requirement of U1C in mediating the compound’s effect, consistent with our in vitro observations (Fig. 5C). These results were confirmed for several exons by end-point PCR, where decreases in exon inclusion could be visualized upon U1C KD (Supplementary Fig. 10A, B), which could be restored upon re-expression of full-length U1C (Supplementary Fig. 10C). We will refer to these as class II exons for clarity in later discussions.

To better understand the effect of U1C KD on the potency of splicing modulators, we analyzed the compound responses of class II exons in greater detail. Two distinct effects were observed across the dose response of either branaplam or risdiplam. The first type of response (type I), was represented by numerous class II exons, where exon inclusion was at a similar level in U1C KD relative to mock at the lowest compound concentration, but was unable to achieve maximal exon inclusion (Emax) even at the highest concentration, strongly suggesting that U1C is required to either establish or stabilize the binding site for small molecule splice modulators (Fig. 8F, G, Supplementary Fig. 11A, B). Since the binding site for splicing modulators is likely the complex between the U1 snRNA and the pre-mRNA 5′ ss, this data suggests that U1C is critical to stabilize a specific structure(s) that is conducive to compound binding and ultimately splicing of noncanonical GA/GU exons. The second type of response (type II), was represented by exons whose compound dose response curvs were right shifted upon U1C KD (Fig. 8H and Supplementary Fig. 11C). A majority of these exons achieved a similar Emax, either at the same concentration seen in mock or at a higher dose for some. This type of response suggests that loss of U1C alters the binding affinity of the small molecule splicing modulators for their target binding site, the U1 snRNA–5′ ss interface, such that the compound can only achieve its full potential of exon inclusion at high concentrations. For both types of response, there were examples where both branaplam and risdiplam were equally effected and ones where the response was compound specific (Fig. 8G and Supplementary Fig. 11C).

Intriguingly, we also observed a set of low inclusion exons where compound activity was greatly enhanced upon U1C knockdown (Fig. 8I). In most cases, both branaplam and risdiplam demonstrated a similar increase in potency, while for other targets such as *EXTL2* and *PPARG* (Fig. 8I and Supplementary Fig. 11D), only branaplam activity was enhanced. We refer to these as class III exons. Combined with the observation that U1C knockdown alone is sufficient to cause inclusion of many cassette exons, this suggests that U1C may suppress splicing of a subset of exons or the effect of compounds in a context-dependent manner and once U1C levels are reduced, the activity of compounds that promote inclusion of these exons can be demonstrated.

## Discussion

To understand the mechanism of how small molecule splicing modulators improve the recognition of weak 5′ ss with an AGA/GU or ANGA/GU sequence by U1 snRNP, we first evaluated the effect of branaplam on the binding affinity between purified native U1 snRNP and either a *SMN2* or *HTT* 5′ ss oligo. We showed that branaplam consistently increased binding affinity by two to threefold (Fig. 1B, C, F). We next showed that branaplam also dramatically increased the binding affinity between either the *SMN2* or *HTT* 5′ ss oligo and U1 snRNP reconstituted from protein components purified from *E. coli* and in vitro transcribed U1 snRNA (Fig. 1D–E). Interestingly, we noticed that the native U1 snRNP bound the 5′ ss much better than reconstituted U1 snRNP (24–37 folds and ~threefold in the absence and presence of branaplam). Our reconstituted U1 snRNP was composed of proteins expressed and purified from *E. coli* and in vitro transcribed U1 snRNA. They lacked the post-translational modifications on human proteins and U1 snRNA (such as the pseudoU on nucleotides 5 and 6 of U1 snRNA^29^), which potentially contributed to the difference in binding affinities. Furthermore, we synthesized a number of branaplam analogs with various degrees of activity in inhibiting *SMN2* exon 7 skipping in cells (Fig. 3A). The rank order of the in vitro activity of these analogs was identical to their in vivo cellular activities in the splicing reporter assay (Fig. 3), supporting that the in vitro reconstituted U1 snRNP contains all key components required for branaplam activity and revealing the structure and activity relationship of branaplam analogs (Supplementary Fig. 3B). The activity profiles of these compounds for improving the binding of U1 snRNP to either the *SMN2* or *HTT* 5′ ss are strikingly similar to their cellular activities in preventing *SMN2* exon 7 skipping (Fig. 3B, C). Ishigami and colleagues speculated that branaplam binds to the *SMN2* and *HTT* motifs in two very different modes^19^. However, the similar activity profile of the branaplam analogs for both the *SMN2* and *HTT* 5′ ss suggests that branaplam binds to both motifs using a similar mode.

We further showed that when the A^−1^, G^−2^, and A^−3^ nucleotide in the *HTT* oligo, or A^−1^ and A^−4^ nucleotide in the *SMN2* oligo were mutated, branaplam no longer increased the binding affinity between reconstituted U1 snRNP and these 5′ ss oligos (Fig. 4). These observations are consistent with the branaplam responsive sequence motifs observed in cells using massive parallel splicing assays and RNAseq experiments^18,19^, indicating that the reconstituted U1 snRNP contains all key elements required for branaplam’s effect on U1–5′ ss binding. The failure of a mutated *HTT* or *SMN2* 5′ ss with U^−1^ bulge to respond to branaplam demonstrates that the nucleotide identity at the −1 bulge is a key determinant for branaplam recognition (Fig. 4A, D).

To further understand the determinants for branaplam function, we took advantage of the modular nature of the reconstituted U1 snRNP. We evaluated the RNA components of U1 snRNP and showed that a U1 snRNA and *HTT* 5′ ss duplex alone was not sufficient for the branaplam effect (Supplementary Fig. 5). We showed that Helix H of U1 snRNA was important for branaplam binding in vitro (Fig. 6). Disrupting the first set of base pairs (G12–C122) in Helix H abolished the effect of branaplam. Restoring this to a (U12–A122) basepair restored the response to branaplam, suggesting that the basepair nature of Helix H is important for the effect of branaplam. White and colleagues demonstrated that a U1 snRNP containing a minimal U1 snRNA construct (60 nt in length, with Helix H maintained but most of the stem loops 1–3 beyond Helix H replaced by a kissing loop) modified from the one used for crystallographic studies in Kiyoshi Nagai’s lab^10^ also responded to branaplam^22^, supporting the importance of Helix H in compound response. We further evaluated the effect of Helix H mutants on GA/GU 5′ ss recognition by U1 snRNP in vivo. Utilizing a U1-GA variant, we demonstrated that compensatory changes that maintain base pairing in Helix H (G^12^A-C^122^U or G^12^C-C^122^G) were functional, establishing a critical role for base pairing at the base of Helix H in vivo. Interestingly, there is a significantly larger effect on loss of splicing with the G^12^C mutant than with the C^122^G mutant, suggesting that the identity of a guanosine at position 12 is also important for the proper function of U1 snRNA in vivo. It is unlikely that these results arised from altered expression levels of variant U1 snRNAs based on three additional pieces of evidence. First, we showed that U1 snRNA variants with Helix H reconstituted equally well as WT U1 snRNP in vitro (Supplementary Fig. 1C). Second, we tested two additional sets of U1 snRNA variants that induced splicing of exons with 5′ ss containing UG and UU at −2 and −1 positions and demonstrated that these sets of variants displayed nearly identical changes to the splicing of a distinct set of iExons elicited by these U1 variants (compared to the U1-GA variant) when combined with Helix H mutations. Third, the compensatory Helix H variants G^12^A-C^122^U and G^12^C-C^122^G, both of which carry mutations at these two sites, were fully functional and resulted in splicing activity that was nearly identical to their parent U1-GA variant with WT Helix H (Fig. 7, Supplemental Fig. 6C).

Helix H is formed by base pairing between nucleotides 119–122 and 12–15, whereas the remaining 11 nucleotides at the 5′ end of U1 snRNA upstream of Helix H are flexible. Disruption of Helix H is therefore expected to untether or relax the 5′ end of U1 snRNA without compromising the overall structural stability of the U1 snRNP (especially a single nucleotide substitution at the end of Helix H (G^12^U)). Indeed, U1 snRNP with Helix H mutant reconstituted similarly to the WT U1 snRNP (Supplementary Fig. 1C) and retained the ability to bind the *HTT* 5′ ss (Fig. 6B and C). However, even with a single nucleotide change at the end of Helix H, branaplam could no longer enhance the interaction between U1 snRNP and weak 5′ ss in vitro. Our in vivo data demonstrate that maintaining base pairing at the base of Helix H generally affected 5′ ss recognition and usage. Together with our in vitro data showing direct effect of Helix H integrity on compound activity, the data from our in vivo experiments suggests that the structural configuration of Helix H play a critical role in the ability of the U1 snRNP to promote productive splicing and likely in the ability of branaplam to enhance U1 snRNP’s interaction with non-canonical 5′ ss.

We also systematically evaluated the role of all U1-specific proteins in branaplam-mediated weak 5′ ss recognition. The binding of branaplam to U1 snRNP and the 5′ ss and its dependency on U1C had been described previously using a minimal U1 snRNP^22^. This minimal complex contained truncated Sm proteins, lacks U1A, includes only a 61-residue fragment of U1C (out of 159 residues), a 59-residue fragment of U1–70 K (out of 437 residues), and a 60-nucleotide U1 snRNA (out of 164 nt) with an engineered kissing loop. As a result, it is difficult to determine whether observations made using this minimal U1 snRNP (such as the dependency on U1C) reflects physiological behavior or arise from the absence of many native U1 snRNP components. Using a near native U1 snRNP (differing only by a short C-terminal truncation of SmB and removal of the C-terminal RS domain of U1–70 K , with all other proteins and U1 snRNA being full-length), we unambiguously demonstrated the dependency of branaplam activity on U1C (Fig. 5). U1C has been proposed to stabilize the interaction between weak 5′ ss and  U1 snRNP through direct interaction with the RNA duplex formed upon U1 snRNP binding^10^. Intriguingly, we found that branaplam enhanced the interaction between U1C and U1 snRNP in the presence of weak 5′ ss (Fig. 5E, F), potentially because U1C prefers to bind the U1 snRNA and 5′ ss duplex. This observation reveals an unexpected dual mechanism of action for branaplam: strengthening the interaction between U1 snRNP and the weak 5′ ss while also enhancing the U1C association with the U1 snRNP, with these effects potentially acting synergistically.

We further considered two possible roles for U1C in splicing modulator function. The first is that U1C functions to promote a conformation among the ensemble of possible structures of the U1 snRNA and 5′ ss duplex that is conducive to compound binding, similar to what has been proposed in other systems^30,31^. The second possibility, as suggested by White et al., is that compound binding depends on direct interaction with U1C, which is required for modulation of U1-GA/GU 5′ ss recognition^22^. To gain transcriptome-wide insight into these two potential models for U1C’s role in compound activity, we tested the effect of U1C KD in vivo on splicing modulator potency by RNA-seq. Our data demonstrated that U1C knockdown broadly reduced the effect of branaplam and risdiplam (Fig. 8B and Supplementary Figs. 9A–C and 11). While the respective sequence specificities of branaplam and risdiplam did not change upon U1C knockdown, the majority of compound-induced GA/GU iExon inclusion events displayed geomean 0.5 log decrease in potency. These in vivo data, based on splicing at over a thousand exons, illuminate two potential mechanisms for the role of U1C in splicing modulation. The consistent loss of splicing potency across a majority of compound-targeted exons suggests that U1C reduction has a general effect on splicing efficiency consistent with the known role of U1C in 5′ ss recognition. In addition to this global effect, analysis of the shift in compound potency of class II exons uncovered two types of splicing response upon U1C KD. For type I response, the inability of compound to promote exon inclusion to the same Emax seen in cells containing wild-type levels of U1C strongly suggests that U1C is directly involved in establishing a specific binding site recognized by splicing modulators at GA/GU 5′ ss engaged with U1 snRNP. For type II response, these exons exhibited a right-shifted response to splicing modulator upon U1C KD. This suggests that U1C is directly affecting the binding affinity for splicing modulators such that the compound can only achieve its full potential in exon inclusion at high concentrations. This effect on affinity could be through either a decrease in association of compound or an increase in the dissociation of compound to the GA/GU 5′ ss-U1 interface. The reduced Emax and rightward shift observed following U1C KD in type II exons are consistent with the critical role of U1C in splicing modulator activity that we observed in vitro.

The RNA-seq results further revealed two additional classes of exons that provided unique insights into the role of U1C function and its effect on splicing modulators. Class I represents a set of U1C-suppressed cassette exons wherein U1C KD increased exon inclusion in the absence of compound (comparing U1C KD and scramble control in DMSO, Supplementary Fig. 8A, B). These cassette exons did not display any bias for exonic 5′ ss sequences and were never responsive to compound. In this class of events, the presence of U1C could be enhancing the stability of structures that are not conducive to 5′ ss recognition and progression toward splicing. This notion is supported by observations in yeast showing that hyper-stabilization of U1-5′ ss interaction inhibits splicing, and that this defect can be rescued by weakening the interaction between U1C and U1 snRNP^26,32^. Class III is identified by cassette exons, which hade GA/GU 5′ ss and were either fully skipped or included at very low levels and responded poorly to compound treatment when U1C expression was normal. Only upon reduction of U1C expression did these exons become highly responsive to compound treatment, leading to their inclusion (Fig. 8I and Supplementary Data 2). Interestingly, several events, such as splicing of *GXYLT1* and *PPARG* iExons, responded in a compound-specific manner (Fig. 8G and Supplementary Fig. 10D), suggesting that these are compound-specific effects independent of U1C KD.

These in vivo studies allowed us to validate our in vitro observations and uncover unexpected insights into the function of U1C and splicing modulators by including transcriptome-wide analysis of endogenous substrates of splicing modulators not represented in focused in vitro studies. The identification of classes I and III of exons revealed a previously unrecognized role of U1C in suppressing inclusion of a subset of exons or the effect of compounds in a context-dependent manner. For class I exons, the interaction between U1 snRNA and these 5′ ss may be too tight in the presence of U1C, which will hinder subsequent steps in the splicing cycle (such as the removal of U1 snRNP and transition of 5′ ss base pairing to U6 snRNA). The reduction of U1C can potentially release its suppression of exon inclusion. However, class III exons are mostly noncanonical, and the binding affinity is likely suboptimal, making this scenario unlikely. Although the exact mechanism of U1C-mediated suppression of this subset of exons remains unclear, the fact that two distinct chemical classes of splicing modulators are active on several classes of exons only upon U1C reduction makes it unlikely that direct contact between U1C and compounds is a driving force of compound activity. Instead, our data are consistent with a model whereby the main role for U1C is to stabilize a specific structural conformation at the interface between U1 snRNA and a GA/GU 5′ ss, such as those found in *SMN2* or *HTT*, which can be targeted by the compound. The noncanonical nature of targeted exons, where intronic sequence is the canonical GUAAG and exonic sequence is a noncanonical GA sequence, strongly suggests that U1C promotes stabilization within the noncanonical NAGA and ANGA exonic portion of the U1 snRNA-5′ ss complex. In this model, Helix H, positioned in close proximity to the exonic portion of the 5′ ss, likely provides additional stability, as changes that alter the stability abrogate the ability of the compound to bind a U1-5′ ss in vitro. Similar changes that alter the stability of Helix H are detrimental to exon inclusion in vivo, demonstrating the importance of structural conformation within this region for 5′ ss recognition. In the context of the class III exons, we hypothesize that U1C can decrease the prevalence of the conformation recognized by branaplam and risdiplam, which therefore allows for compound activity to be enhanced only upon knockdown of U1C. Understanding the critical role of protein and RNA elements in driving a specific conformation conducive for compound binding has broad implications for the development of other splicing modulators and RNA-targeting small molecules in general.

Finally, the lack of activity of risdiplam on either the native or reconstituted U1 snRNP’s ability to bind *SMN2* 5′ ss in vitro, even in the presence of U1C (Fig. 2), suggests that additional cellular factors may be needed for the risdiplam effect. One such candidate is the LUC7L family of alternative splicing factors (LUC7L, L2, and L3). These proteins are homologs of the yeast Luc7 protein, which binds to the U1 snRNA and 5′ ss RNA duplex on the opposite side of U1C and stabilizes the duplex together with U1C^33,34^. The presence of exons whose response to branaplam is strongly enhanced, but whose response to risdiplam remains unchanged upon U1C KD (Supplementary Fig. 11D), is consistent with the requirement of other cellular factors for the effect of risdiplam, which may render these exons less sensitive to U1C KD. It is possible that additional cellular factors, such as LUC7L proteins, could contribute to the binding of risdiplam in a similar manner as U1C, by altering the ensemble of structures formed to present a more favorable binding site for the compound.

## Methods

### Engineering HTP tag on endogenous U1A in HeLa cells

Parental HeLa-S3 cell lines used in this study were from Cell Technologies Shared Resource of the University of Colorado Anschutz Medical Campus. HeLa-S3 cells were routinely cultured adherently in MEM medium (Corning) supplemented with 10% FBS and 1× penicillin-streptomycin (Invitrogen).

To generate C-terminal fusions of a HTP (HA-His10-TEV-protA) tag in-frame with U1A gene, two guide RNAs (gRNAs) target close to the stop codon of U1A were selected using the gRNA design tool CRISPOR. Each gRNA was inserted into pX330 as described by ref. ^35^ (Supplementary Table 1). Linear doner DNA fragments were generated by PCR using primers U1ADF/U1ADR to incorporate 100 bp up and downstream of the stop codon of U1A as the homology arms to an HTP-T2A-neo cassette (the template plasmid pAC5-HTP-T2A-neo was a gift from David Bentley^36^). Two phosphorothioate linkages were included at the 5′ end of each primer. HeLa-S3 cell culturing adherently in 60 mm dish were co-transfected with 2 μg each of pX330 gRNA plasmid and linear donor DNA using Lipofectamine LTX (Thermo Fisher Scientific). G418 (800 μg/mL) was added 72 h after transfection and the medium was changed every 2–3 days until the G418-sensitive cells were eliminated, and then G418 concentration was dropped to 400 μg/mL. After 2–3 weeks, colonies were transferred to 24-well plates, and genomic DNA was isolated for PCR genotyping. For the U1A-HTP colonies, U1AendF /U1A3UTRR (363 bp product) were used to screen for the presence of the wild-type allele, and U1AendF /HTPR (757 bp product) were used to screen for incorporation of the HTP-T2A-neo cassette. The PCR products were confirmed by Sanger sequencing. Western blots were performed using anti-HA antibody (clone 3F10, Roche, 100 ng/ml final concentration) to further confirm the fusion of the tag to U1A.

### Native U1 snRNP purification

U1A-HTP cells were adapted to grow in suspension in Joklik’s MEM (Sigma) supplemented with 5% (v/v) newborn calf serum and 1× Pluronic™ F-68 (Gibco). Cells were cultured in 2-liter flasks on an orbital shaker (~ 110 rpm) at 37 °C and maintained at a density of 0.4–1 × 10^9^ cells/L throughout the culture period. 8–10 liters of cells at density of ~1 × 10^9^ cells/L were harvest and washed with PBS. The cell pellet was snap-frozen for native U1 snRNP purification.

The cell pellet was thaw on ice and gently resuspended in 5 pellet volumes of cold Buffer A (10 mM Hepes pH 7.9, 10 mM KCl, 1.5 mM MgCl2, 0.5 mM DTT) and left on ice for 10 min. The cell suspension was centrifuged for 20 min at 25,000 × *g* at 4 °C. The supernatant was removed. The nuclei pellet was resuspended in cold Buffer IP 250 (20 mM Hepes pH 7.9, 250 mM NaCl, 1.5 mM MgCl_2_, 0.05% NP-40, 0.5 mM DTT) with protease inhibitors (Sigma), and sonicated 2–3 times for 10 s at 40% amplitude. The lysate was centrifuged for 30 min at 25,000 × *g* and the supernatant was incubated with 0.5–1 ml of preequilibrated IgG Sepharose 6 affinity resins (Cytiva) for 2–3 h. The resins were then washed with buffer IP 250, IP 200 (same with IP 250 with 200 mM NaCl), IP 150 (same with IP 250 with 150 mM NaCl) without NP-40. The U1 snRNP was released by TEV cleavage O/N at 4 °C. The purified U1 snRNP was further concentrated using an Amicon centrifugal concentrator and analyzed by SDS-PAGE and RNA gel electrophoresis to confirm the presence all U1 snRNP proteins and U1 snRNA.

### Pull-down experiment

The *SMN2* pre-mRNA fragment used in pull down experiment contains 100 nt up and downstream of the 5′ ss of *SMN2* intron 7 followed by three copies of MS2 tag. It was cloned downstream of a T7 promoter in the Sp72 vector. The *SMN2* pre-mRNA was in vitro transcribed and purified by 7 M Urea denaturing gel.

0.5 μM of *SMN2*-MS2 (pre-incubated with 50-fold excess of MBP-MS2 protein) was incubated with 0.3 μM of reconstituted or native U1 snRNP in buffer (20 mM Hepes, pH 7.5, 150 mM KCl, 3 mM MaCl_2_, 1 mM TCEP), in presence of 50 μM of Branaplam or 0.5% DMSO. The complex was then incubated with amylose resin and washed with the same buffer. The resins were treated in 5 μl buffer containing 1 mg/ml proteinase K at 37 °C for 30 min to release the RNAs. The resulting samples were run on 6% denaturing polyacrylamide gel and stained with Sybr Gold to visualize the RNAs.

### Expression and purification of individual U1 snRNP protein components

The plasmids used for individual protein components of human U1 snRNP expression were kindly provided by Dr. Frédéric Allain^23^. These include Sm B (1–174)-D3, Sm D1–D2, and Sm E-F-G, U1A, U1C, and U1–70 K (1–216). *Escherichia coli* BL21 (DE3) (Novagen) strains transformed with the above plasmids were grown to an OD_600_ of ~0.6 at 37 °C and then induced with 0.5 mM isopropyl β-D-thiogalactoside (IPTG) for an additional 20 h at 16 °C.

Cells expressing these proteins were harvested by centrifugation and lysed by sonication. Individual components were purified following the protocol described by the Allain lab^23^. More specifically, the SmB-D3, U1A, and U1C proteins were purified by sequential Ni-NTA metal ion affinity chromatography (Qiagen), cation-exchange chromatography (SP Sepharose Fast Flow; GE Healthcare), and gel filtration (Superdex 75 columns, GE Healthcare) chromatography. SmD1-D2 was purified by cation-exchange followed by gel filtration. SmE-F-G and U1–70 K were purified by Ni-NTA and gel filtration. Purified proteins were concentrated using a Millipore concentrator and stored at −80 °C.

### In vitro transcription and purification of U1 snRNA

The DNA template for full-length U1 snRNA was cloned into the pSP72 plasmid following a T7 promoter. The recombinant vector was linearized by PstI restriction digestion at 37 °C overnight and subsequently purified by phenol: chloroform extraction followed by ethanol precipitation. Large-scale in vitro transcription was performed in the presence of 40 mM MgCl2 at 37 °C for 3 h. The transcription mixture was applied to denaturing urea polyacrylamide gel electrophoresis (PAGE) and total RNA was extracted, purified using phenol: chloroform extraction followed by ethanol precipitation, dissolved in DEPC-dd H_2_O and stored at −80 °C. Site-specific U1 snRNA mutants were generated by PCR-based method and verified by DNA sequencing. The purification procedure of mutant U1 snRNAs was identical to that of the wild-type U1 snRNA.

### In vitro reconstitution of U1 snRNP

Prior to complex reconstitution, U1 snRNA was first heated at 80 °C for 3 min, and then cooling immediately on ice for 10 min. The annealed U1 snRNA was then incubated with Sm B-D3, Sm D1–D2, Sm E-F-G in a molar ratio of 1:1.5:1.5:1.5. After incubation at 30 °C for 30 min and at 37 °C for 15 min, the mixture was cooled on ice for 5 min. U1–70 K (1–216) and U1A in equal molar amounts to U1 snRNA were added successively, with an interval of 20 min. After incubated on ice overnight, U1 snRNP ΔU1C was purified by anion exchange chromatography on a 1 ml RESOURCE^TM^ Q (Cytiva) previously equilibrated with buffer B (10 mM Hepes pH7.5, 250 mM KCl, 5 mM DTT, 2 M Urea)  and eluted with a linear gradient of 0.25–2.0 M KCl in buffer C (10 mM Hepes pH 7.5, 2 M KCl, 5 mM DTT). The U1 snRNP ΔU1-C was then dialyzed overnight against buffer D (10 mM sodium phosphate pH6.8, 100 mM NaCl, 5 mM DTT) and quantified. Finally, two-fold molar amounts of U1-C was added. After incubated on ice for 4 h, the mixture was separated by size exclusion chromatography using a Superdex 200 increase column in buffer D. Peak fractions containing the desired U1 snRNP complex were collected and concentrated.

### Fluorescence polarization assays for U1 snRNP binding to 5′ ss oligo

All of 5′-FAM labeled RNA oligos used in this study were synthesized and purified by IDT (USA). Fluorescence polarization (FP) assays were performed in binding buffer (20 mM HEPES pH 7.5, 1 mM MgCl2, 200 mM KCl, 0.01% TritonX-100, 0.1%PEG3350, 2%DMSO) at room temperature using a Envision HTS Plate Reader system (Revvity). Each reaction system contains 3 nM of FAM-labeled RNA oligo, reconstituted U1 snRNP (or native U1 snRNP, or full-length U1 snRNA, or the 5′end of U1 snRNA) at twofold increasing concentrations, 800 nM (for reconstituted U1 snRNP) or 400 nM (for native U1 snRNP) U1C, different small molecules in DMSO (10 μM unless otherwise stated). The equilibrium dissociation constants (K_d_) of binding reaction were calculated using the following single-site binding model formula in GraphPad Prism:

$$y={START}+\frac{({END}-{START})x}{{k}_{D}+x}$$

where *x* and *y* represent the concentration of U1 snRNP (or U1 snRNA) and the polarization value measured respectively, START represents the polarization value of protein-free nucleic acid probe, END represents the maximum polarization value when U1 snRNP (or U1 snRNA) binds to the fluorescently labeled 5′ ss oligo. All FP experiments are carried out in two distinct biological replicates and error bars represent the standard deviation.

### Fluorescence polarization assays for U1 snRNP binding to U1C

Cy5-NHS was added to U1C in amine-free reaction buffer (50 mM sodium phosphate, pH 6.5, 150 mM sodium chloride) to achieve a 5:1 molar ratio. After incubating the mixture at 4 °C for 24 h, non-reacted Cy5-NHS and reaction byproducts were removed using a desalting spin column. The final concentration of Cy5-U1C was measured by Nanodrop.

Each FP assay contains 40 nM of Cy5-labeled U1C and reconstituted U1 snRNP at twofold increasing concentrations ranging from 0 to 800 nM in the binding buffer (20 mM HEPES pH 7.5, 1 mM MgCl2, 200 mM KCl, 0.01% TritonX-100, 0.1%PEG3350, 2%DMSO) at room temperature. Fluorescence was read using a Envision HTS Plate Reader system (Revvity). The equilibrium dissociation constants (K_d_) of binding reaction were calculated using the same formula as above.

### Compound synthesis

See supplementary information.

### SMN2 splicing minigene reporter assay

The *SMN2* splicing reporter minigene plasmid (pCI-*SMN2*-skipping-GLuc, Addgene #218670) encodes truncated exons 6 and 8, mutated exon 7 (with a single nucleotide insertion), and truncated introns 6 and 7 of the human *SMN2* gene fused to Gaussia luciferase (GLuc), as previously described^37,38^. Exon 7 inclusion places the GLuc gene out of frame, thereby reducing luciferase activity. HEK293T cells (ATCC, CRL-11268, authenticated by manufacturer using STR profiling) were cultured at 37 °C with 5% CO_2_ in complete growth medium containing 10% FBS (Cytiva, #SH3039603HI) in DMEM (Thermo, #11995073) supplemented with Antibiotic-Antimycotic (Thermo, #15240062). Transfection was performed in 10 cm dishes using Lipofectamine 2000 (Thermo, #11668019) according to the manufacturer’s protocol. 6–16 h post-transfection, cells were trypsinized (Thermo, #12605010) and seeded into 384-well plates (Greiner, #781080) that had been preloaded with compounds via acoustic dispensing. Compounds were dispensed in duplicate across a 1:3 serial dilution series ranging from 0.012 to 9 µM. Following a 36 h incubation at 37 °C, luciferase activity was assessed by adding 12 µL of a custom luciferase reagent to each well. The luciferase reagent (12 mL total volume) consisted of 60 µL 50× coelenterazine substrate (Nanolight, #320), 3 mL Nanolight GLuc buffer, 30 µL 10% Triton X-100 (Thermo, #A16046.AP), and 8.9 mL deionized water. Plates were shaken at 600 rpm for 6 min, and luminescence was measured using a Cytation 5 multimode plate reader (BioTek). Half-maximal effective concentration (EC_50_) values were calculated using GraphPad Prism (v10.4) with four-parameter nonlinear regression curve fitting.

### Helix H U1-GA, U1-UG, and U1-UU variants and RNA sequencing

Synthesis of the U1 variants constructs, growth of HEK293 cells (ATCC, CRL-1573, authenticated by manufacturer using STR profiling), transfection, and RNA isolation were described earlier^18^. Briefly, wild-type and U1 minigene constructs were designed at PTC and synthesised at GenScript^®^. For U1 constructs 5 × 10^5^ HEK293 cells were transfected with 2 μg of plasmid deoxyribonucleic acid (DNA) or mock control in six-well plates, using 6 μl Fugene6^®^ (Promega) according to the manufacturer’s instructions. Total RNA was extracted using the RNeasy Plus Mini Kit. RNA concentration and quality were assessed using a NanoDrop spectrophotometer (ThermoFisher Scientific). For library preparation and sequencing, mRNA was enriched from ~3 μg of total RNA using oligo(dT) beads. The mRNA was fragmented randomly using fragmentation buffer followed by complementary DNA (cDNA) synthesis using an mRNA template and random hexamers primer. Second-strand synthesis buffer (Illumina), deoxynucleotides, ribonuclease H, and DNA polymerase I were added to initiate second-strand synthesis. After a series of terminal repair, A-ligation, and sequencing adaptor ligation, the double-stranded cDNA library was completed through size selection and PCR enrichment. RNA libraries were sequenced in a HiSeq sequencer (Illumina).

### siRNA knockdown of U1C and RNA sequencing

HEK293T cells were grown in DMEM media with 10% FBS. 500,000 cells in 1.75 mL of media were plated into a 6-well plate for approximately 6 h. Either siRNA negative control (ThermoFisher Silencer^TM^ Negative control #2, AM4613) or U1C siRNA (ThermoFisher Silencer^TM^ Select Pre-designed siRNA, S13225) were transfected for a final concentration of 25 nM using Invitrogen Lipofectamine RNAiMax (13778-150). In brief, 10 μL 5 μM siRNA was added to 115 μL of OptiMEM, while 5 μL Lipofectamine added to 120 μL OptiMEM for 5 minu before mixing the solutions and incubating for 20 min. 250 μL of Lipofectamine:siRNA solution was gentle added to the 1.75 mL cells. After 24 h post-transfection, cells were treated with 10 μL compound resuspended in DMSO. Final concentrations of branaplam were 1.2 μM, 400, 100, 25, and 5 nM. Final concentrations of risdiplam were 3.6 μM, 1.2 μM, 400, 200, and 50 nM. Each compound-treated experimental condition was treated and collected in duplicate, while four DMSO only treated samples per siRNA transfection were collected. RNA was isolated using KingFisher^TM^ Apex instrument and the MagMAX™ *mir*Vana™ Total RNA Isolation Kit (A27828) by Thermo Fisher according to manufacturer’s protocol. Isolated RNA was submitted to Admera Health for library preparation (polyA selected, stranded) and mRNA-sequencing and >20 M 150 bp pair-end reads were returned.

### RT-qPCR validation of U1C knock down

BioRad iTaq universal SYBR green reaction mix (Bio-Rad 172-5150) was used according to manufacturer’s protocol with modifications. For a total reaction volume of 10 μL, 50 ng of total RNA was amplified with U1C forward and reverse primers (Supplementary Table 1) at a final concentration of 300 nM. Reactions were initial incubated at 50 °C for 10 min followed by heat inactivation of RT at 95 °C for 1 min. Two-step PCR amplification was performed at 95 °C for 10 s and 60 °C for 30 s for 40 cycles followed by measurement of a melt curve. PPIA primers (Supplementary Table 1) were used to measure an endogenous control and fold change was calculated using ΔΔ Ct method.

### Evaluation of U1C and SmB levels in whole cell lysate and U1 snRNP after U1C knockdown

Transfection of siU1C for knockdown in HEK293 was performed similarly as for RNA sequencing experiments above. RNA was extracted with a Qiagen RNeasy Plus Mini kit (74134) after lysate homogenization with a Qiashredder (79656). RT-qPCR validation of U1C knockdown is also similar as above, except for use of the New England Biolabs Luna One-Step RT-qPCR kit (E3005S). For protein studies, 48 h cell pellets were lysed with 50 mM Tris-HCl, pH 7.5, 150 mM NaCl, 1% Igepal, 0.25% deoxycholate, 0.1% SDS, and 1× protease inhibitor cocktail (P3100-010) and sonicated with a Diogenode Bioruptor Pico for 2 s × 3 cycles with 1 m rest on ice between cycles. Lysates were clarified by centrifugation at 18,000 × *g* for 20 m at 4 °C. For immunoprecipitation, lysate was incubated with U1A antibody (Santa Cruz Biotechnology sc-376027, 1:25 dilution) for 2.5 h at 4 °C, followed by incubation with Protein G beads (ThermoFisher Scientific, 10004D) 1 h at 4 °C. IP samples were washed 3 times with IP Wash Buffer (50 mM Tris-HCl, pH 7.5, 150 mM NaCl, 0.1% Igepal), and eluted with 2× Laemmli sample buffer followed by boiling for 5 m. Whole cell lysates and IP samples were immunoblotted with anti-U1C (Santa Cruz Biotechnology sc-101549, 1:500 dilution), anti-U1A (Santa Cruz Biotechnology sc-376027, 1:1000 dilution), and anti-SmB (Y12, gift from Dr. Joan Steitz’s lab^39^, 1:1000 dilution). Conformation-specific secondary antibodies (Rockland 18-8817-30, 1:4000) were used for immunoblot.

### RNAseq processing and splicing analysis

RNAseq and splicing analysis were described earlier using DEDseq (https:// github.com/liwc01/DEDSeq^18^) with minor modifications. RNA sequencing reads were mapped to human genome (hg19) using STAR (version 2.7.10b); only uniquely mapped reads (with MAPQ > 10) with <5 nt/100 nt mismatches and properly paired reads were used. For splicing analysis, all junction reads (read with a gap in alignment indicating splicing) were used, including the ones mapped to unannotated splice sites. Reads were counted for different exons (for cassette exon [CE]) or exonic regions (for A5′ss or A3′ss). For each splicing event, a percent-spliced-in (PSI) value was calculated using the percent of average read number supporting the inclusion among all reads supporting either the inclusion or the exclusion. A minimum of 10 for the denominator of PSI calculation was required. Otherwise, a “NA” value would be generated. PSI values for biological replicates were averaged, and the PSI difference between the two treatment groups was calculated. For a statistical test, a 2 × 2 read counts table was made for each event, with rows for reads supporting inclusion or exclusion, and columns for the two comparing sample groups. Fisher’s exact test was used for the statistical tests. For biological replicates, each pair of the testing and control sample was analyzed by Fisher’s exact test and the geometric mean of all *P*-values was reported as a final *P*-value. PSI change (deltaPSI) of >10% (or < −10%) and *P* < 0.001 was used to select splicing events being regulated by the treatment. PSI calculation, Fisher’s exact test were performed using R (4.0.5). 5′ ss composition analysis was performed as described in ref. ^25^. Potency of splicing modulator was determined by plotting dPSI and identifying the concentration at which the change in PSI reached 30% and reported as EC_dPSI30.

### RT-PCR analyses of iExon inclusion

A total of 500,000 SH-SY5Y cells were plated in 6-well tissue culture plates. After 4 h of recovery, cells were transfected with final 100 nM siRNA (silencer select s13212 or negative control No.2 from Ambion) using Lipofectamin RNAi Max (ThermoFisher) according to the manufacturer’s protocol. After 24 h incubation, cells were treated with splicing modulating compounds for additional 24 h. Cells were lysed in RLT buffer ( + beta-mercaptoethanol) and RNA was extracted using the RNeasy kit (Qiagen) according to the manufacturer’s protocol.

Knockdown was confirmed by RT-qPCR using TaqMan assays (Hs00863364_gH, Hs02758991_g1, from ThermoFisher). Splicing changes were confirmed by endpoint PCR using the primer sets listed in Supplementary Table 1. Relative amounts of *U1C* RNA were calculated for control knockdown vs. U1C knockdown (normalized to *GAPDH* expression level).

HEK293 cells were transfected for siRNA in 6-well plates, similarly as for RNA-sequencing above. Sixteen hours later, the media was changed to include DMSO or branaplam (100 nM or 400 nM). Overexpression of 1.5 μg pMEV-2HA-EV or pMEV-U1C-2HA was carried out with Lipofectamine3000 (L3000008) 6 h later. RNA was harvested 24 h later, extracted as above, and reverse transcribed with a Qiagen QuantiTect RT kit (205311). For end-point RT-PCR, 30 ng cDNAs were used in 30 μL reactions with Platinum II Hot Start Green PCR Mastermix (14001012), the primer pairs (*SCO1*: CAACTCTGCCAGATCTAACTCC and TATGTAGTCTTCATCTTCGTCCTTG; *DPH1*: CGGCGCTGGTCGTATCC and CATTTGCAAGGCCACCTTCTT), at 66 °C Tm, and 28 cycles. The amplicons were resolved on 6% polyacrylamide gels at 100 V for 1.5 h and gels incubated with 0.5 μg/ml ethidium bromide for 30 min prior to imaging.

### Reporting summary

Further information on research design is available in the Nature Portfolio Reporting Summary linked to this article.

## Supplementary information

Supplementary Information

Description of Additional Supplementary Files

Supplementary Data 1

Supplementary Data 2

Reporting Summary

Transparent Peer Review file

## Source data

Source Data

## Data Availability

The data supporting the findings of this study are available from the corresponding authors upon request. The RNA-Seq data was deposited to GEO database under accession GSE304951. Source data for the figures and Supplementary Figs. are provided as a Source Data file. Source data are provided with this paper.
