# Supplementary material for: The U1 snRNP protein U1C and Helix H of U1 snRNA are critical for small molecule splicing modulator function
Source: Nat Commun. 2026 Jul 16;17:8837. doi: 10.1038/s41467-026-74765-8 (PMC13500670; doi:10.1038/s41467-026-74765-8)
Supplement: Supplementary file 1 [file 41467_2026_74765_MOESM1_ESM.pdf]

Supplementary Fig. 1

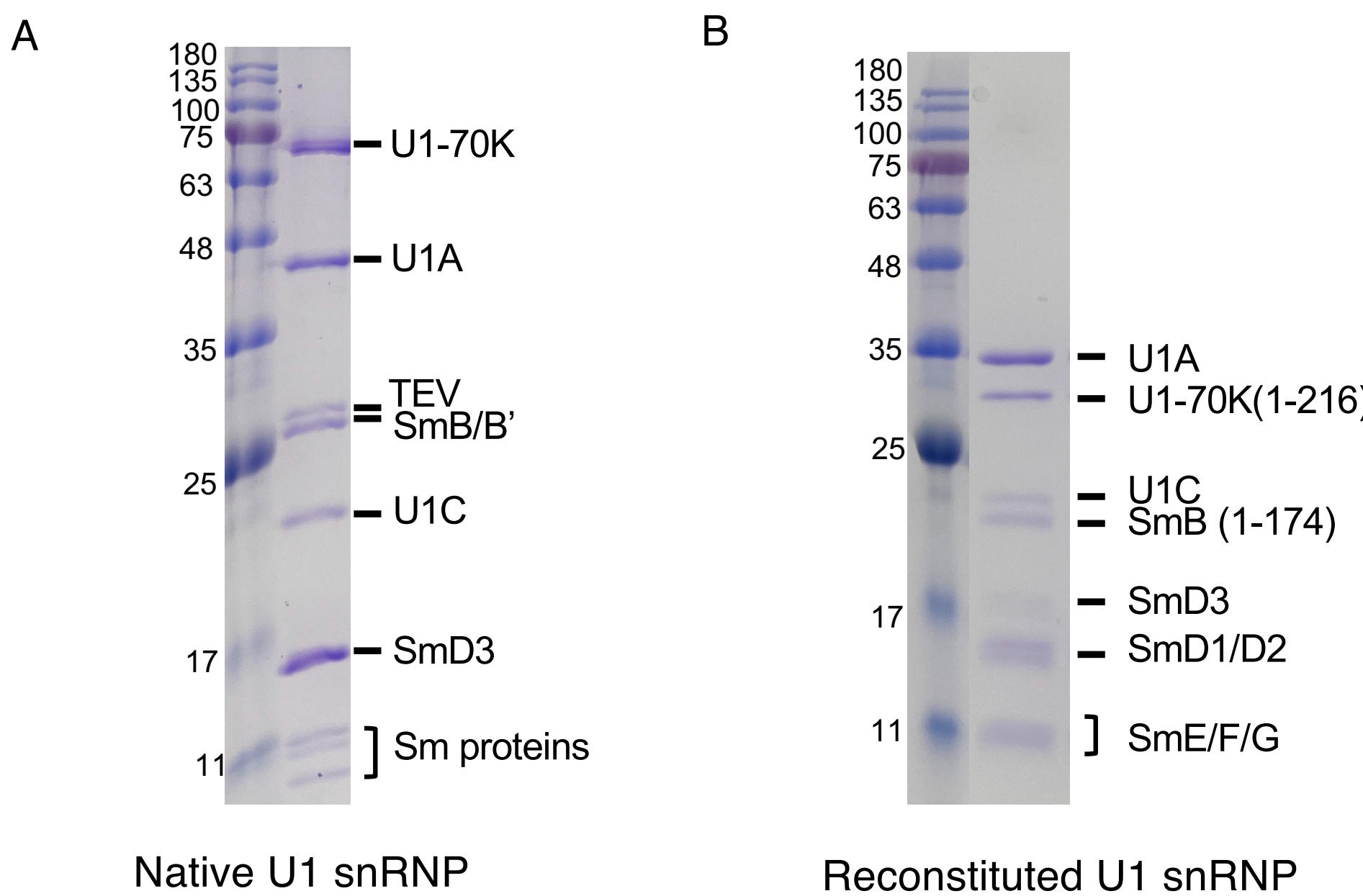

C

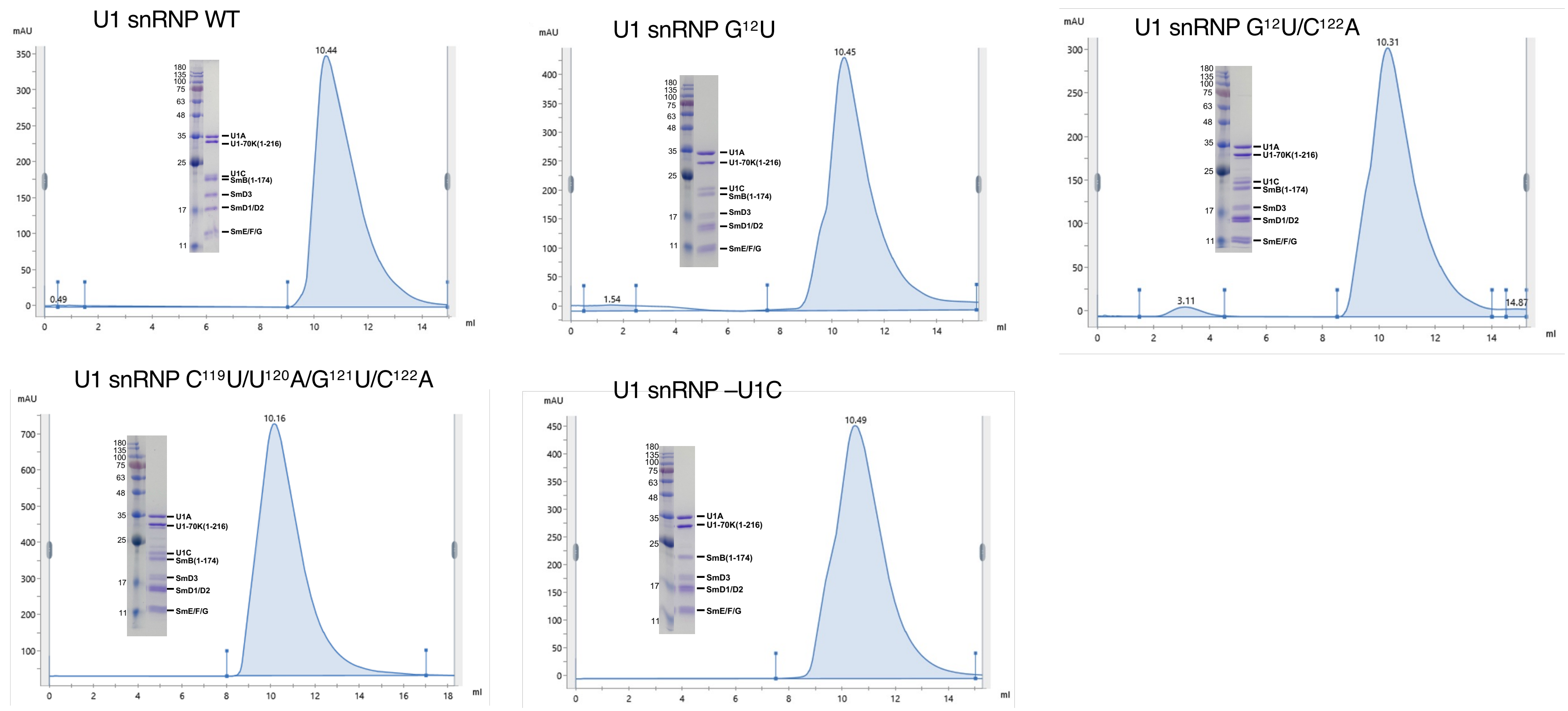

Supplementary Fig. 1. Expression and purification of the native and reconstituted U1 snRNP.

A. Coomassie stained SDS PAGE gel of native U1 snRNP purified from HeLa cells using the protein A tag on U1A.

B. Coomassie stained SDS PAGE gel of U1 snRNP reconstituted from protein components purified from E. coli.

C. Gel filtration profile and Coomassie stained SDS PAGE showing various U1 snRNP mutants and without U1C reconstitute similarly to the WT.

Supplementary Fig. 2

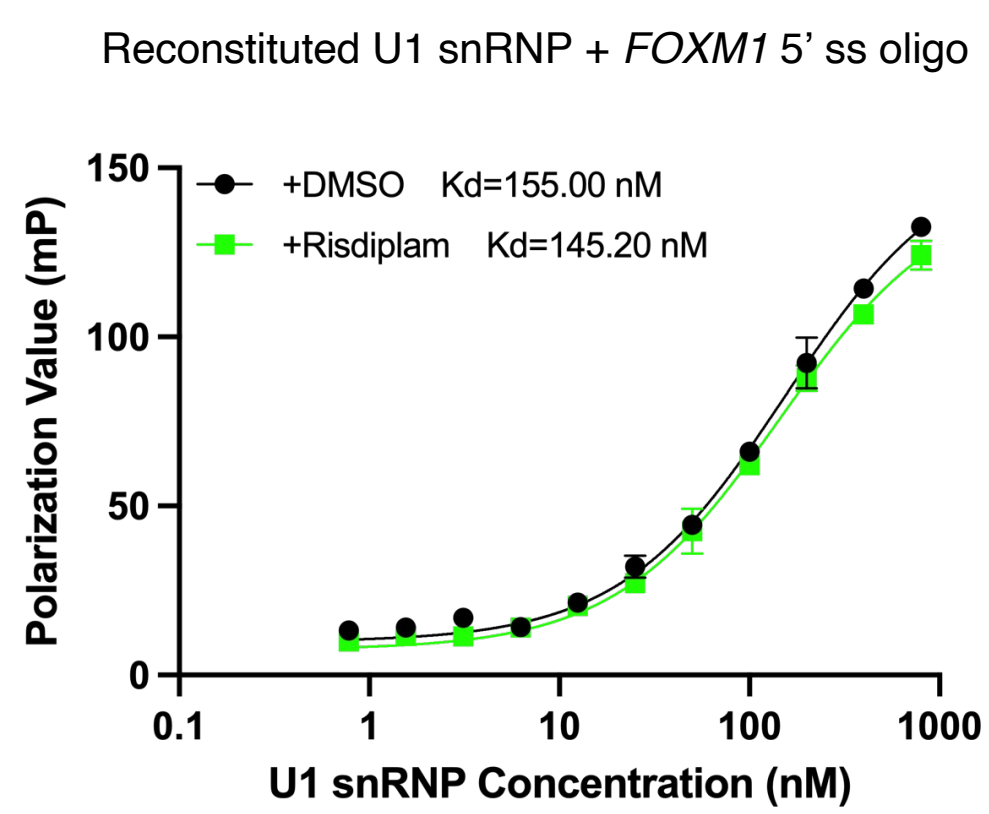

Supplementary Fig. 2. FP experiments using the *FOXM1* 5' ss oligo (A̲UG̲A̲/GUAAGUUC) indicate that risdiplam does not improve the binding of reconstituted U1 snRNP to this oligo that shares the *SMN2* motif (underlined).

Supplementary Fig. 3

A

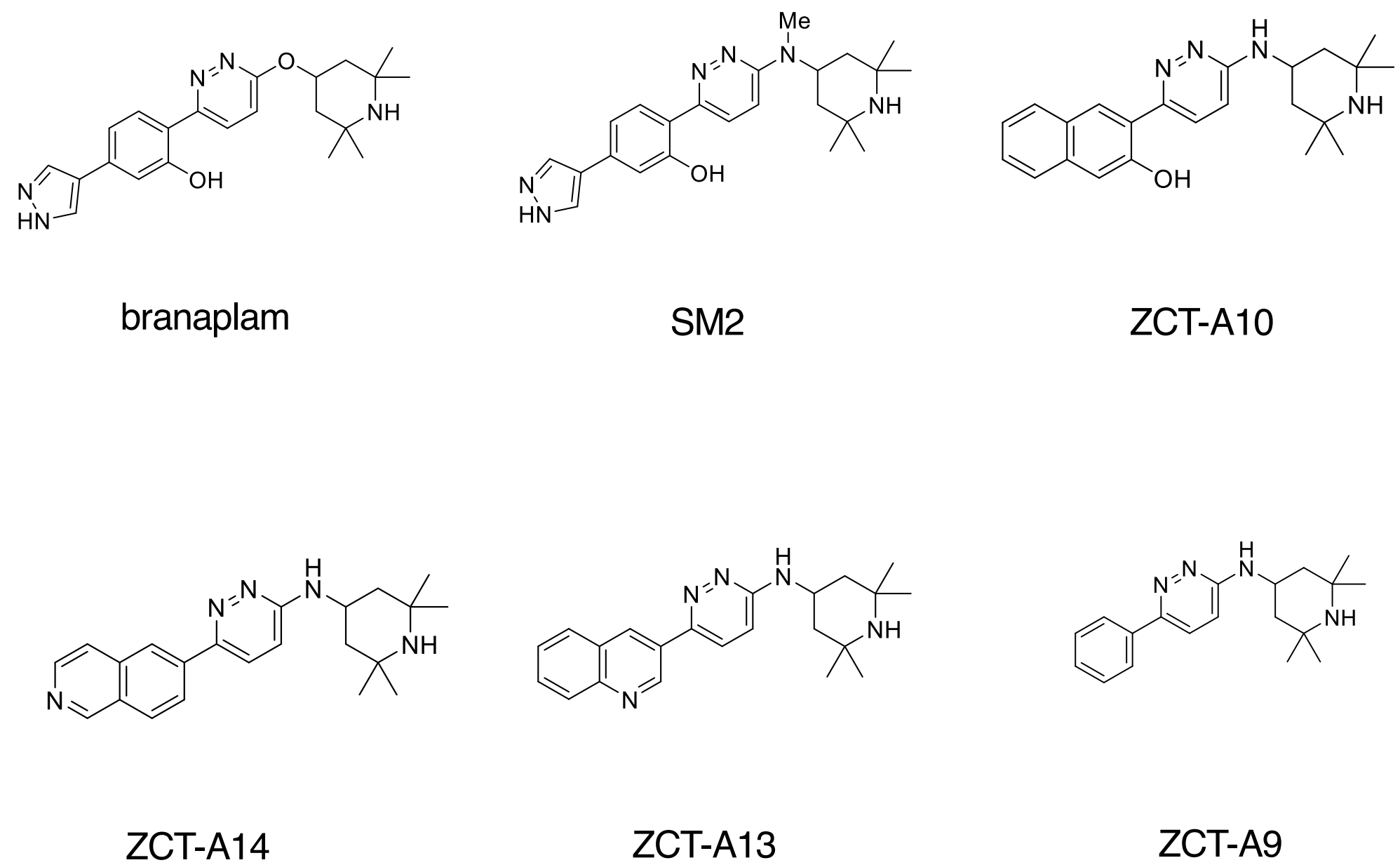

B

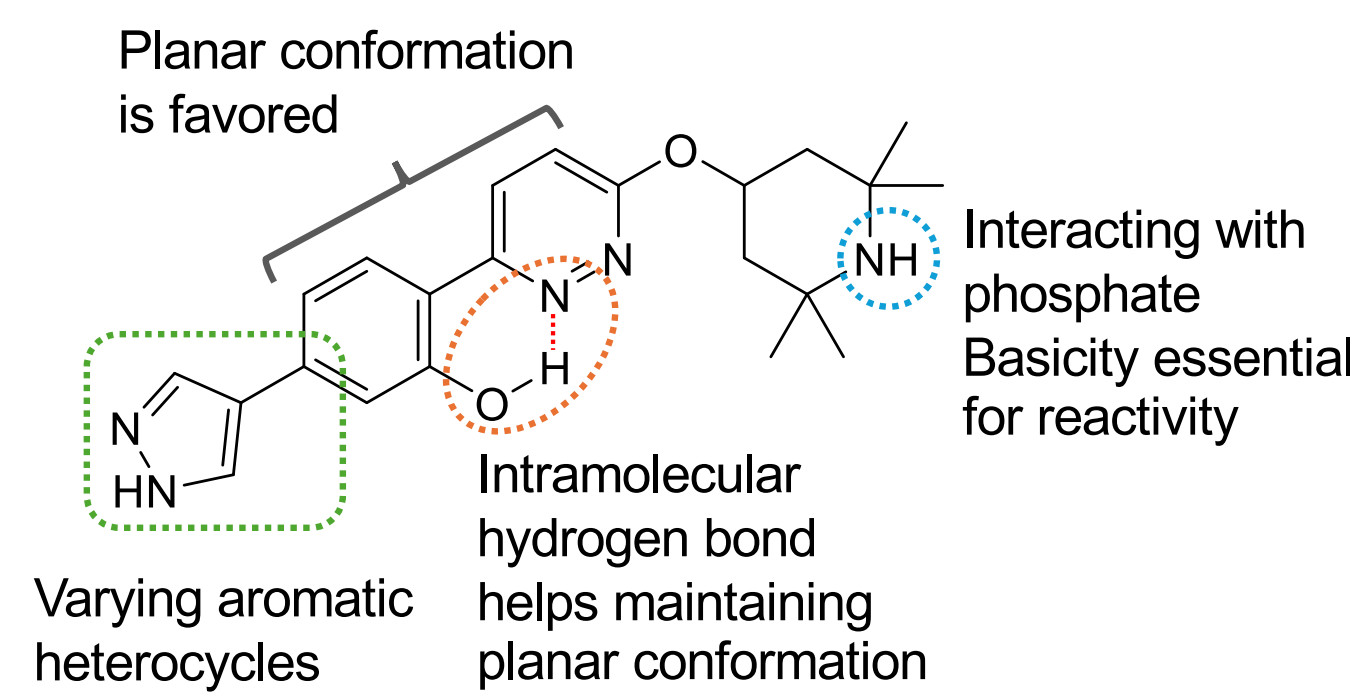

Supplementary Fig. 3. Structures of branaplam analogs (A) and functional groups important for branaplam activity (B).

Supplementary Fig. 4

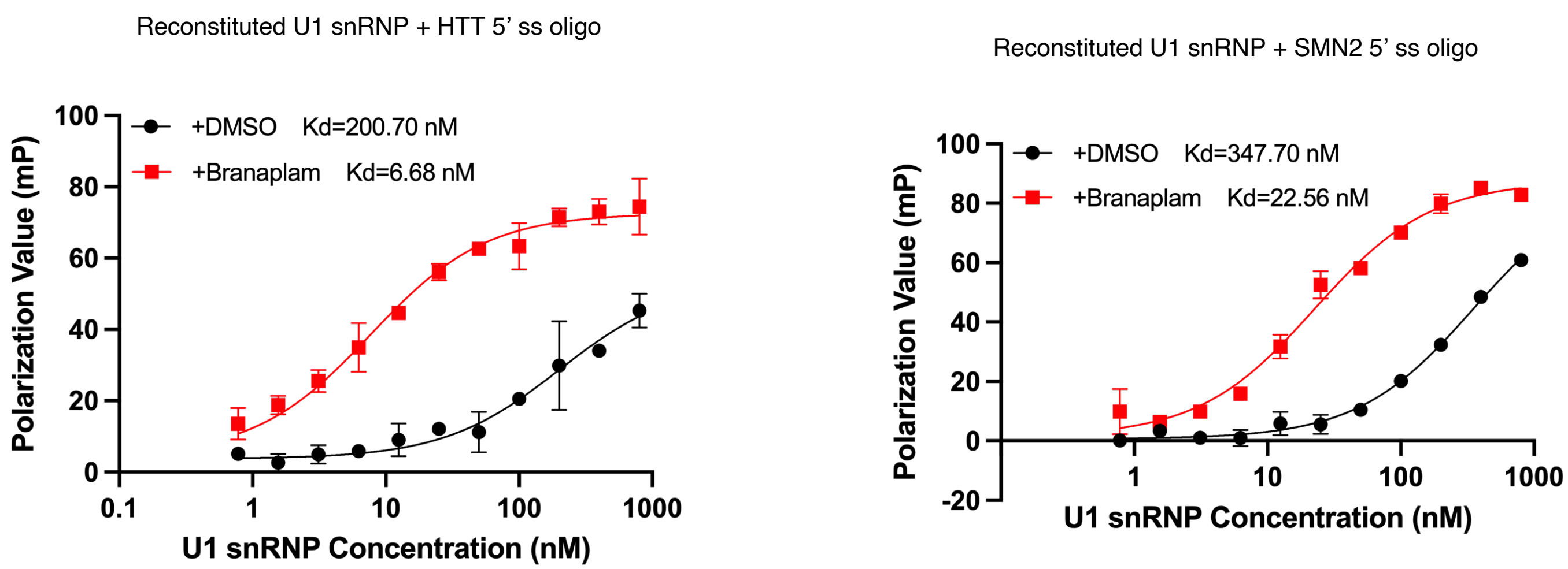

Supplementary Fig. 4. Positive controls for Fig. 4 showing that branaplam increases the binding affinity between U1 snRNP and *HTT* or *SMN2* 5' ss in the experiments conducted in Fig. 4.

Supplementary Fig. 5

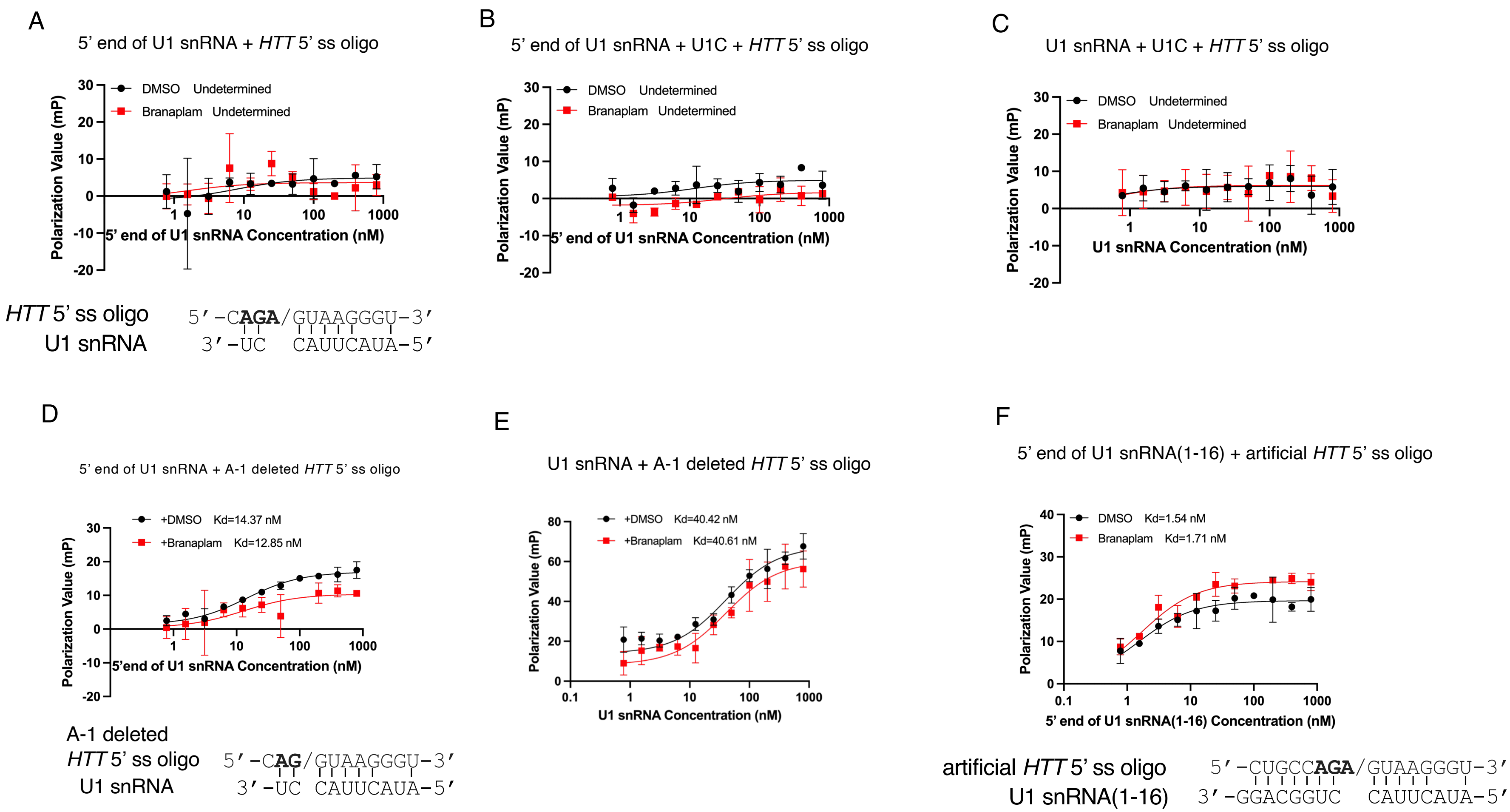

Supplementary Fig. 5. U1 snRNA and 5' ss duplex with the A bulge is not sufficient for the effect of branaplam.
A. FP experiment using fluorescently labeled *HTT* 5' ss oligo and increasing concentration of an oligo representing the 5' end of U1 snRNA (nucleotide 1-10) indicates there is no obvious binding between the two oligos.
B. The same FP experiment carried out as in A with the addition of 800 nM of U1C indicates there is no obvious binding between the two oligos even in the presence of U1C.
C. FP experiment carried out with the full length instead of the 5' end of U1 snRNA and the SMN2 5' ss oligo indicates there is no obvious binding between U1 snRNA and the *HTT* 5' ss oligo even in the presence of U1C.
D. FP experiment carried out with the -1A nucleotide in the *HTT* 5' ss oligo deleted indicates that the removal of the -1A bulge generate appreciable binding between the mutant *HTT* 5' ss oligo and the 5' end of U1 snRNA, but this binding is not improved by the presence of branaplam.
E. The same FP experiment was carried out as in D with the full length instead of the 5' end of U1 snRNA, demonstrating even more substantial binding with the mutant *HTT* 5' ss oligo (with the -1A bulge removed), but this binding is not improved by the presence of branaplam.
F. An artificial *HTT* oligo was designed to basepair with nucleotides 1-16 of U1 snRNA and ensure the presence of a -1A bulge. FP experiments using this oligo with a fluorescent label and nucleotide 1-16 of U1 snRNA indicate that these two oligos bind to each other but this binding is not improved by the presence of branaplam.

Supplementary Fig. 6

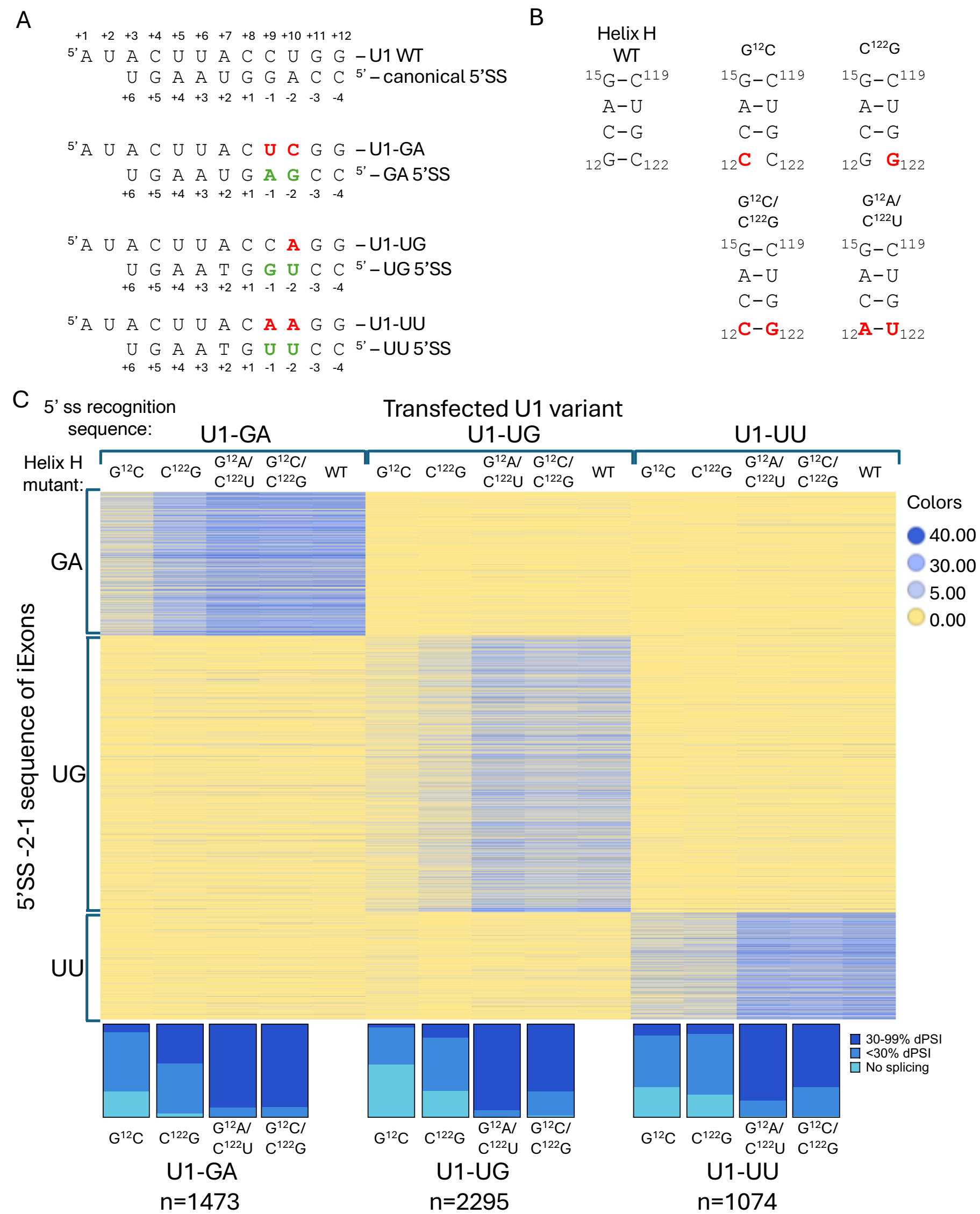

Supplemental Figure 6

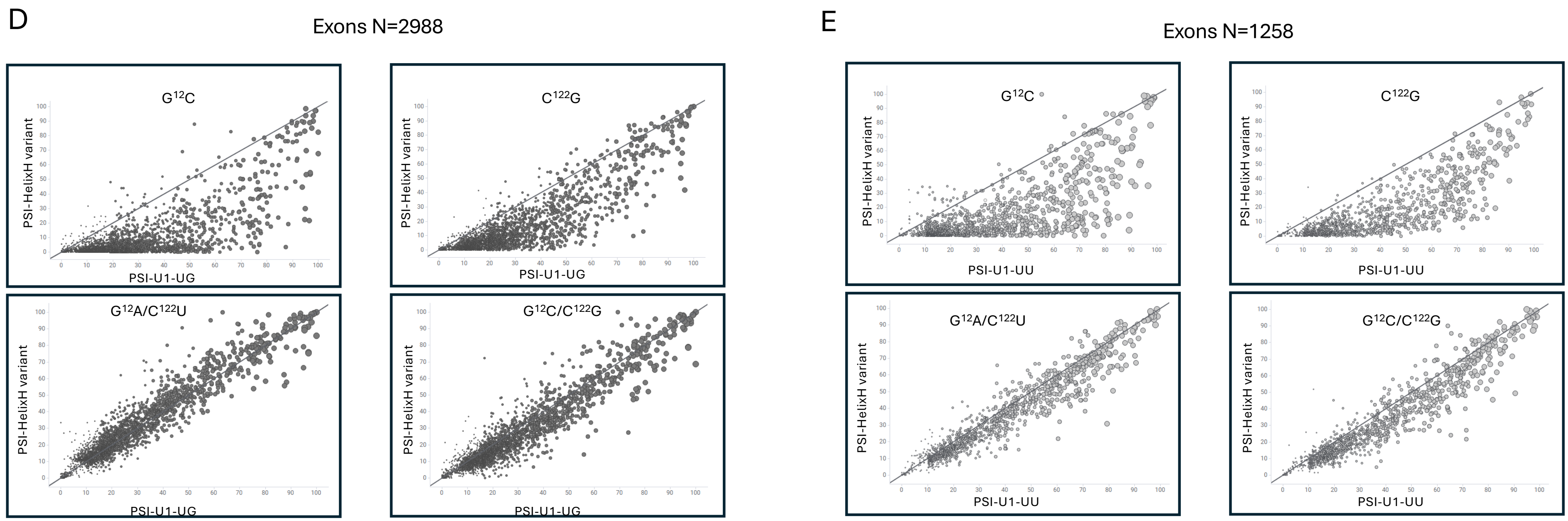

Supplementary Fig. 6. Helix H structure and nucleotide identity are critical to U1 snRNA function in vivo.

U1-GA, U1-UG, and U1-UU variants each carrying mutations in Helix H were expressed in HEK293 cells and then RNAseq was utilized to determine the level of inclusion of exons containing a GA/GU, UG/GU, and UU/GU 5' splice sites.

A. Schematic of 5' ss (bottom 3' to 5') base pairing to U1 WT or variants with substitutions at nucleotides 9 and 10. Highlighted in red are substitution in U1 WT sequence and highlighted in green are the -2/-1 nucleotides of exons induced by these U1 snRNA mutants.

B. Schematic of WT helix H, and $G^{12}C$, $C^{122}G$, $G^{12}A/C^{122}U$ and $G^{12}C/C^{122}G$ mutants.

C. Heat map of 1473 GA/GU 5' ss iExons induced by U1-GA WT, 2295 UG/GU 5' ss iExons induced by U1-UG, and 1074 UU/GU 5' ss iExons induced by U1-UU and corresponding helix H mutants for each, colored by delta PSI between the each U1 variant and mock transfection with larger delta PSI values shown in darker blue and delta PSI 0 in yellow. The heatmap indicates that U1-GA variants, U1-UG variants, U1-UU variants each specifically induced the inclusion of many iExons with GA/GU, UG/GU, and UU/GU 5' ss, respectively. For all U1 5' ss selective variants tested, the $G^{12}C$ and $C^{122}G$ mutants reduced these inclusions, and the $G^{12}A/C^{122}U$ and $G^{12}C/C^{122}G$ mutants largely restored the inclusions. Bar graphs under each column of the heat map are same as Fig. 7C and show comparison of iExon inclusion between U1-GA WT and U1-GA helix H mutants, U1-UG WT and U1-UG helix H mutants, and U1-UU WT and U1-UU helix H mutants as indicated by the percent decrease in inclusion of iExons (PSI_{mutant} / PSI_{WT}*100) in three different ranges: light blue indicates iExon does not splice in U1 helix H variant, medium blue indicates a ratio of <=30%, and dark blue indicates a ratio of >30%.

D. Plot of PSI for all UG/GU exons induced by U1-UG variants. PSI for U1-UG with WT helix H is on the x-axis and PSI for U1-UG carrying each of the four Helix H mutations is on the y-axis. Panels show that the $G^{12}C$ and $C^{122}G$ mutants reduced the inclusions induced by U1-UG WT, and the $G^{12}A/C^{122}U$ and $G^{12}C/C^{122}G$ mutants largely restored the inclusions. Line drawn is x=y.

E. Plot of PSI for all UU/GU exons induced by U1-UU variants. PSI for U1-UU with WT helix H is on the x-axis and PSI for U1-UU carrying each of the four Helix H mutations is on the y-axis. Panels show that the $G^{12}C$ and $C^{122}G$ mutants reduced the inclusions induced by U1-UG WT, and the $G^{12}A/C^{122}U$ and $G^{12}C/C^{122}G$ mutants largely restored the inclusions. Line drawn is x=y.

Supplementary Fig. 7

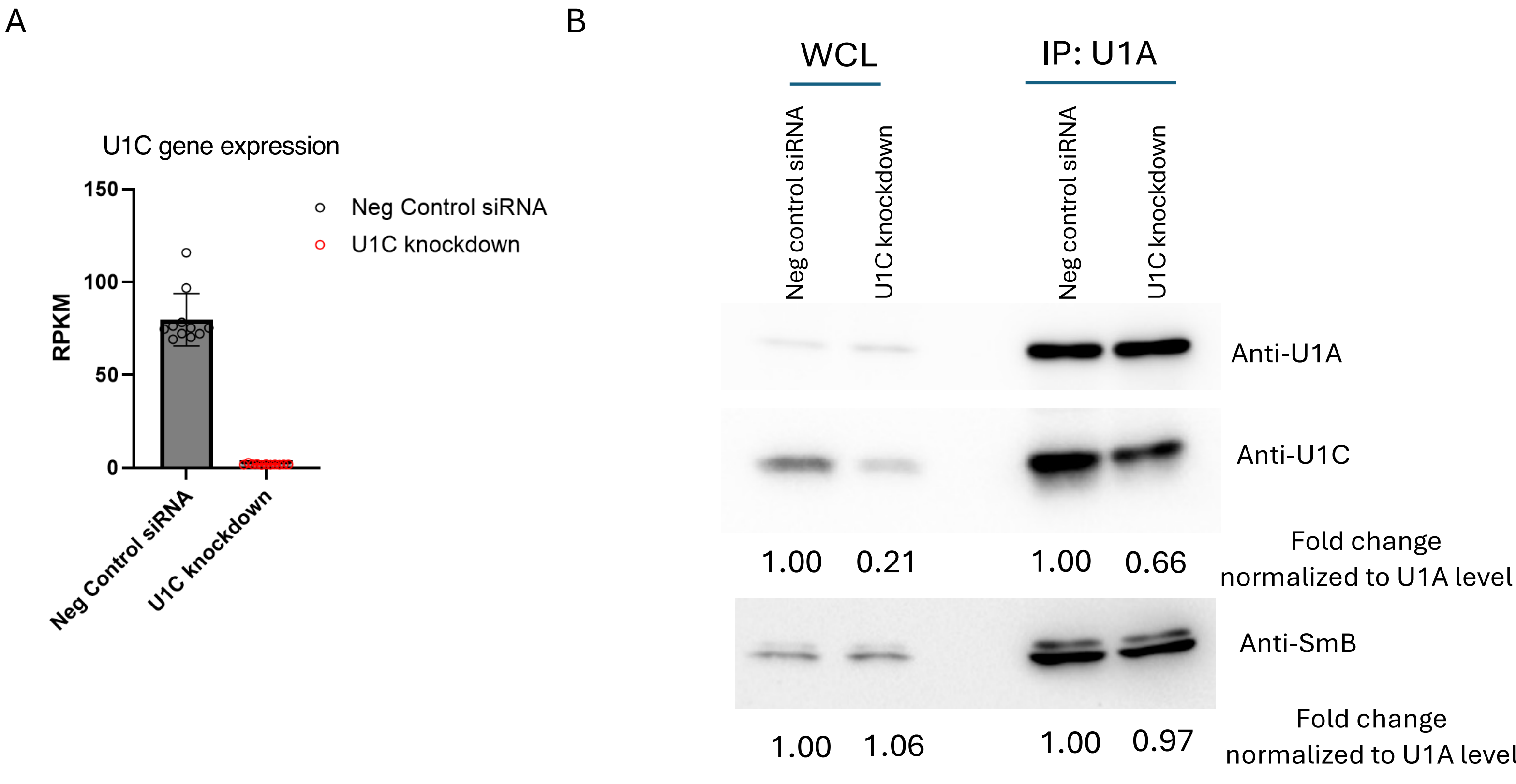

Supplementary Fig. 7. U1C protein level is dramatically reduced in whole cell lysate but remained at a substantial level in U1 snRNP after U1C KD.

A. U1C mRNA level determined by qRT-PCR after U1C KD.

B. U1C, U1A, and SmB protein levels in whole cell lysate (WCL) and U1 snRNP (immunoprecipitated by anti-U1A antibody) determined by Western blot. Some U1C remains in U1 snRNP (possibly due to a slow turnover rate) 48 hours after U1C KD.

Supplementary Fig. 8

A

PSI10_Pfisher0.001			Exon		Alt 5'SS		Alt 3'SS		Retained Introns	
Treatment	Comparison	Total	Included	Skipped	Up	Down	Up	Down	Up	Down
U1C KD	Scramble	14279	782	**8364**	528	222	267	93	2838	1185
branaplam 1.2uM	DMSO	13365	4031	3245	693	176	455	98	3809	858
branaplam 400nM	DMSO	5767	1941	1153	334	76	205	42	1324	692
branaplam 100nM	DMSO	1775	671	196	94	28	75	17	493	201
branaplam 25nM	DMSO	601	208	33	26	8	22	4	189	111
branaplam 5nM	DMSO	298	34	10	4	4	3	1	139	103
risdiplam 3.6uM	DMSO	7809	2438	2045	409	109	266	81	1825	636
risdiplam 1.2uM	DMSO	3488	1170	731	168	44	120	33	961	261
risdiplam 400nM	DMSO	4529	823	361	158	39	241	32	2047	828
risdiplam 200nM	DMSO	576	192	31	18	5	26	5	165	134
risdiplam 50nM	DMSO	240	35	3	4	3	3	0	105	87
branaplam 1.2uM	U1C KD	11920	3775	3675	827	103	387	97	1916	1140
branaplam 400nM	U1C KD	4819	1759	947	373	42	164	36	956	542
branaplam 100nM	U1C KD	1503	613	102	79	14	50	18	401	226
branaplam 25nM	U1C KD	517	141	21	12	3	10	2	193	135
branaplam 5nM	U1C KD	334	30	7	3	2	3	1	172	116
risdiplam 3.6uM	U1C KD	5789	1952	1602	310	65	192	71	987	610
risdiplam 1.2uM	U1C KD	2005	831	260	100	17	77	18	461	241
risdiplam 400nM	U1C KD	904	351	38	35	8	39	6	255	172
risdiplam 200nM	U1C KD	1209	259	74	40	8	76	5	393	354
risdiplam 50nM	U1C KD	1548	275	100	41	15	99	9	522	487

B

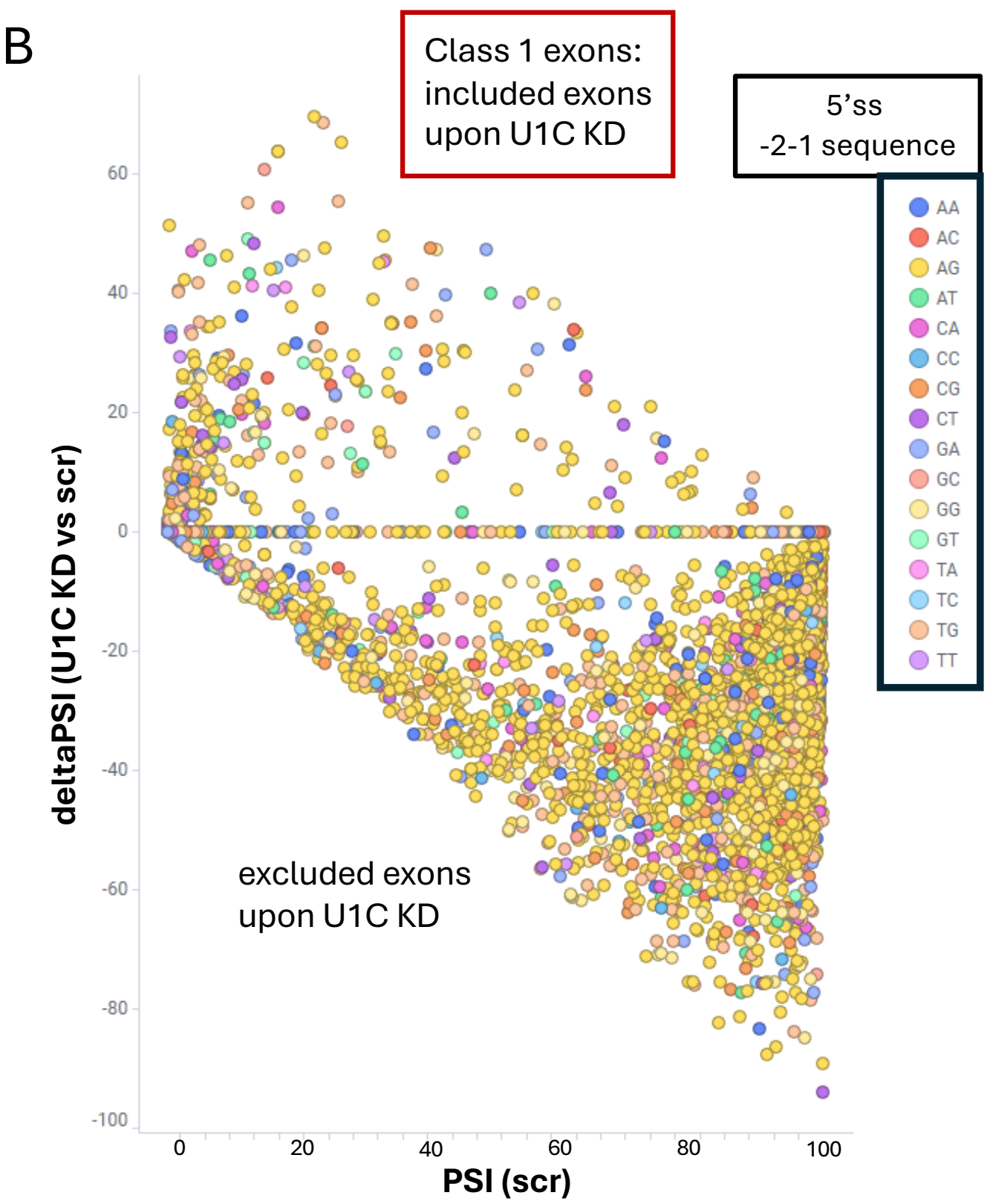

C

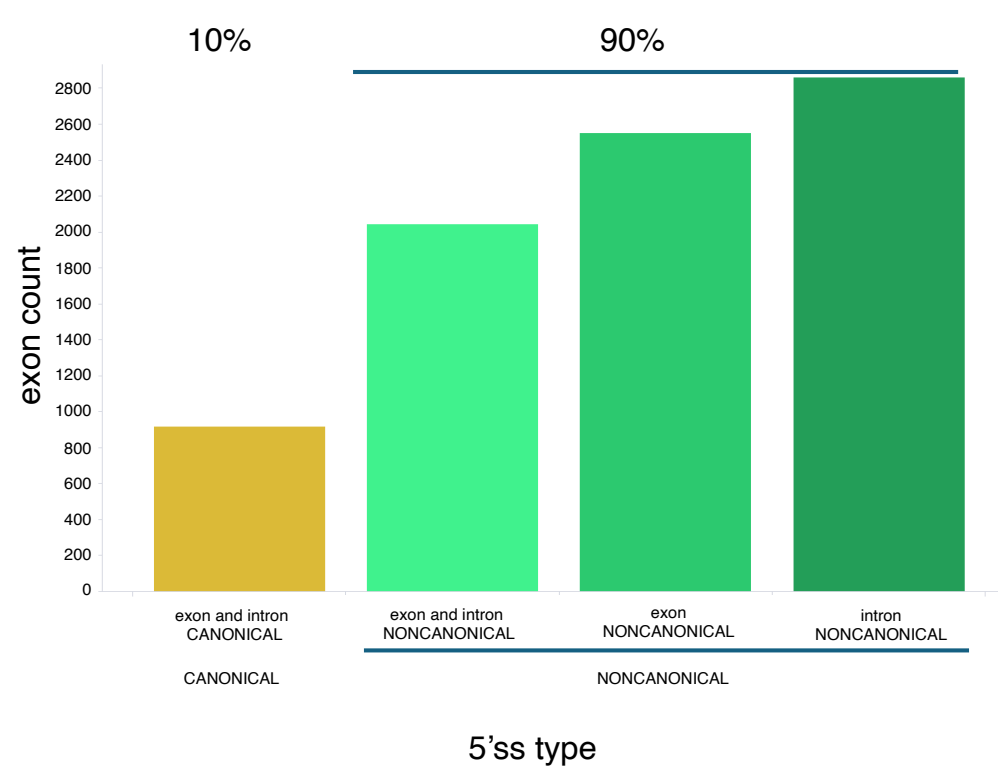

Supplementary Fig. 8. Splicing statistics for U1C Knockdown.

A. A summary table of all splicing changes for U1C Knockdown, including statistics of identified exons (included and skipped), Alternative 5' splice site (Alt 5'SS), Alternative 3' splice site (Alt 3'SS) and Retained Introns. Row 1, U1C siRNA Knockdown compared to negative control siRNA; Row 4-8 and 9-13, dose response branaplam or risdiplam, normalized to negative control siRNA; Row 15-19 and 20-24, dose response branaplam or risidiplam normalized to U1C siRNA knockdown.

B. Scatter plot of cassette exon deltaPSI for all U1C siRNA Knockdown deltaPSI (y-axis) versus PSI of negative control siRNA (baseline PSI; x-axis). Color represents the different -2 and -1 nucleotides at the 5'ss of the exons.

C. Bar graph representation of analysis of 5' ss sequence composition of skipped exons, indicating enrichment for noncanonical 5' ss. Canonical and noncanonical nature of the exonic and intron portions of the 5' ss were calculated as described by Kenny et.al, 2025 [24] for each skipped exon (n=8364) upon U1C KD. If both exon or intron score is canonical, exon is considered CANONICAL; if either or both scores are noncanonical, exon is considered NONCANONICAL.

Supplementary Fig. 9

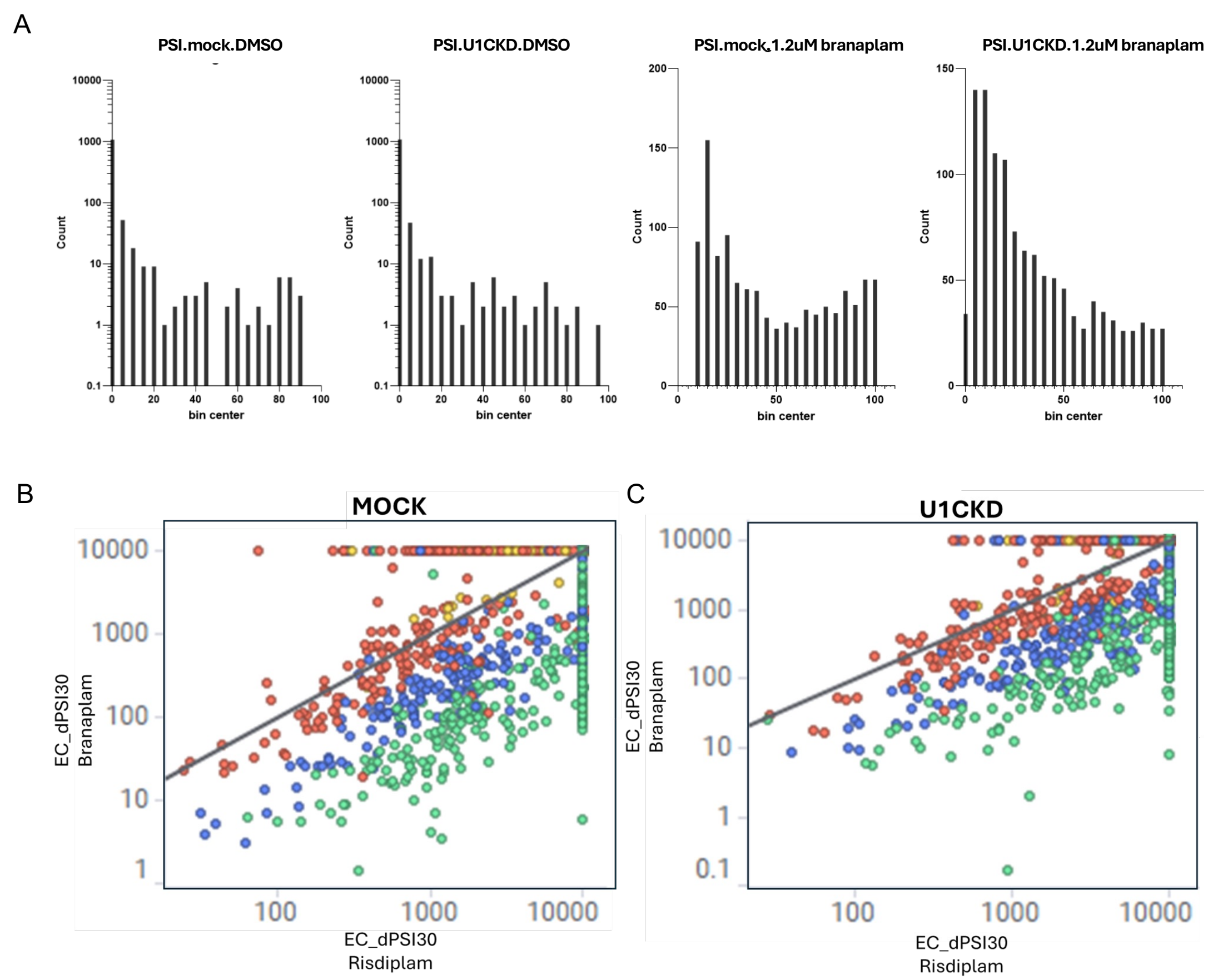

Supplemental Fig. 9. U1C knockdown does not change the sequence preference of branaplam and risdiplam.

A. Bar graph representation of the PSI distribution for all exons included in the analysis of the effect of U1CKD on compound potency. Each panel displays a binned count of PSI values ranging from 0 to 100%. Shown is baseline PSI for DMSO treated and U1C KD compared to the PSI profile upon Branaplam treatment in mock or U1C KD cells. In the mock KD, Branaplam leads to a right shift of the PSI distribution, consistent with its role in inducing more exon inclusions. Upon U1C KD, the distribution is shifted back towards lower PSI, indicating reduced exon inclusion potentially stemmed from a combination of the reduction in U1C level and compound sensitivity.

B. Scatter plot of EC_dPSI30 for Branaplam (y-axis) versus risdiplam (x-axis). Colors and symbols represent sequences found at the -4 or -3 position of the 5' splice site. There is a strong selectivity for -3A (green circles) with Branaplam, -4A (red circles) with Risdiplam and equipotency for -4A -3A (blue circles) with both compounds. Line drawn is x=y.

C. Same as B, but in cells with U1C knockdown. Comparison of panels B and C demonstrates no change to this selectivity upon U1C siRNA knockdown. Line drawn is x=y.

Supplementary Fig. 10

A

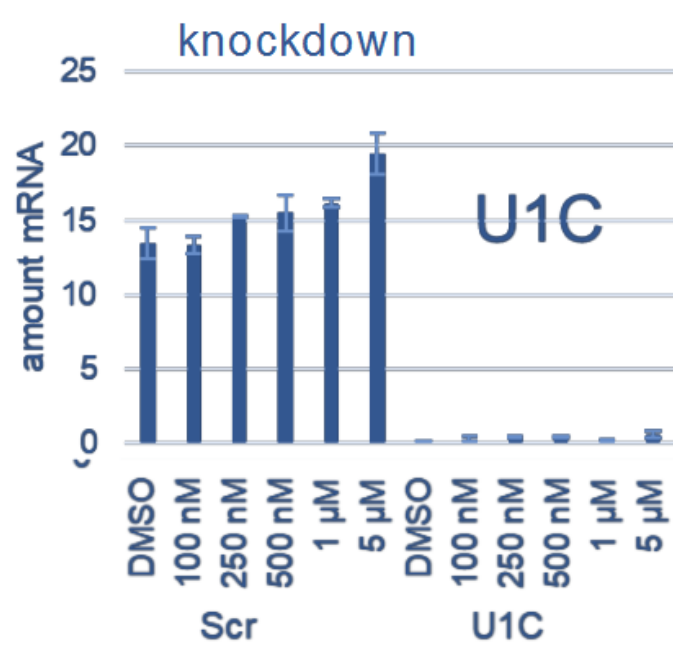

B

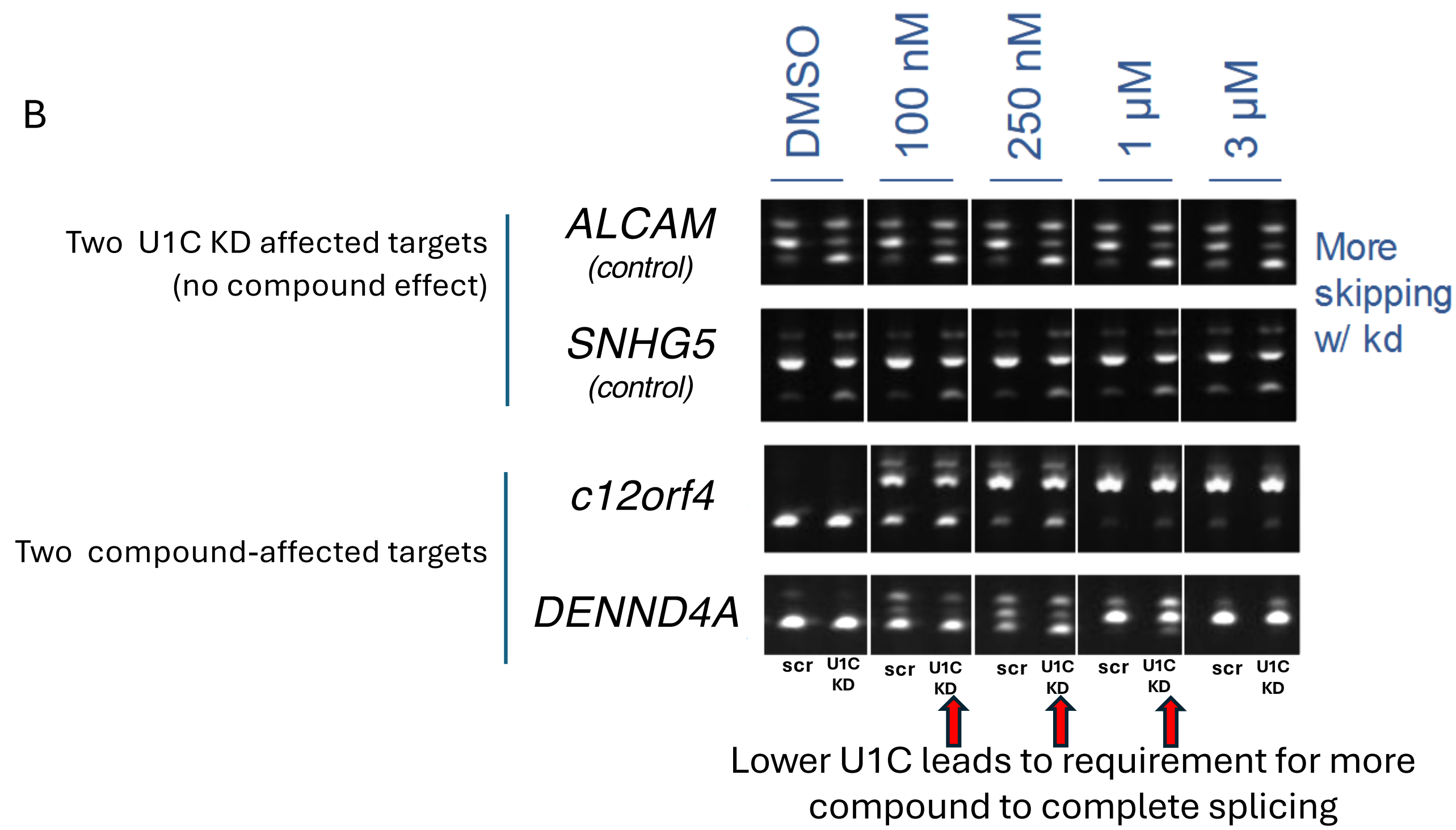

Supplementary Fig. 10

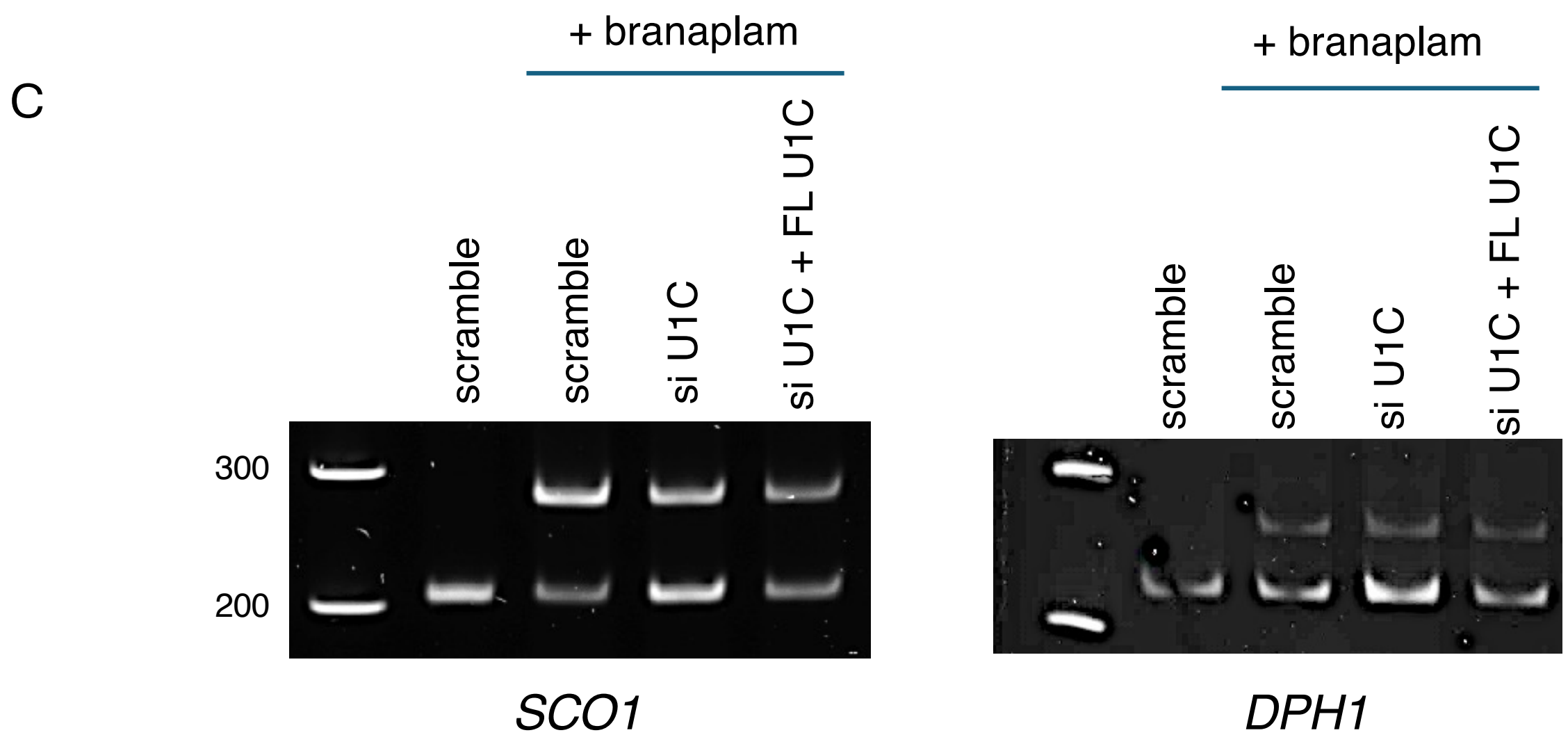

Supplementary Fig. 11. U1C Knockdown reduces branaplam potency as demonstrated by end-point PCR analysis.
A. qPCR measurement of U1C mRNA after siRNA knockdown with scrambled siRNA control and U1C siRNA.
B. End-point PCR for known U1C knockdown affected targets ALCAM and SNHG5 and GA/GU targets of branaplam (c12orf4 iExon 1a and DENND4A) scr control versus U1C KD with compound treatment at 3uM, 1uM, 250nm, 100nM and DMSO control. Red arrows depict the doses at which U1C KD effect on branaplam treatment is observed.
C. RT-PCR showing two representative iExons that was included upon branaplam treatment with scrambled siRNA. The inclusion was reduced upon U1C KD and was rescued by FL U1C addback, indicating the loss of is specific to U1C KD .

Supplementary Fig. 11

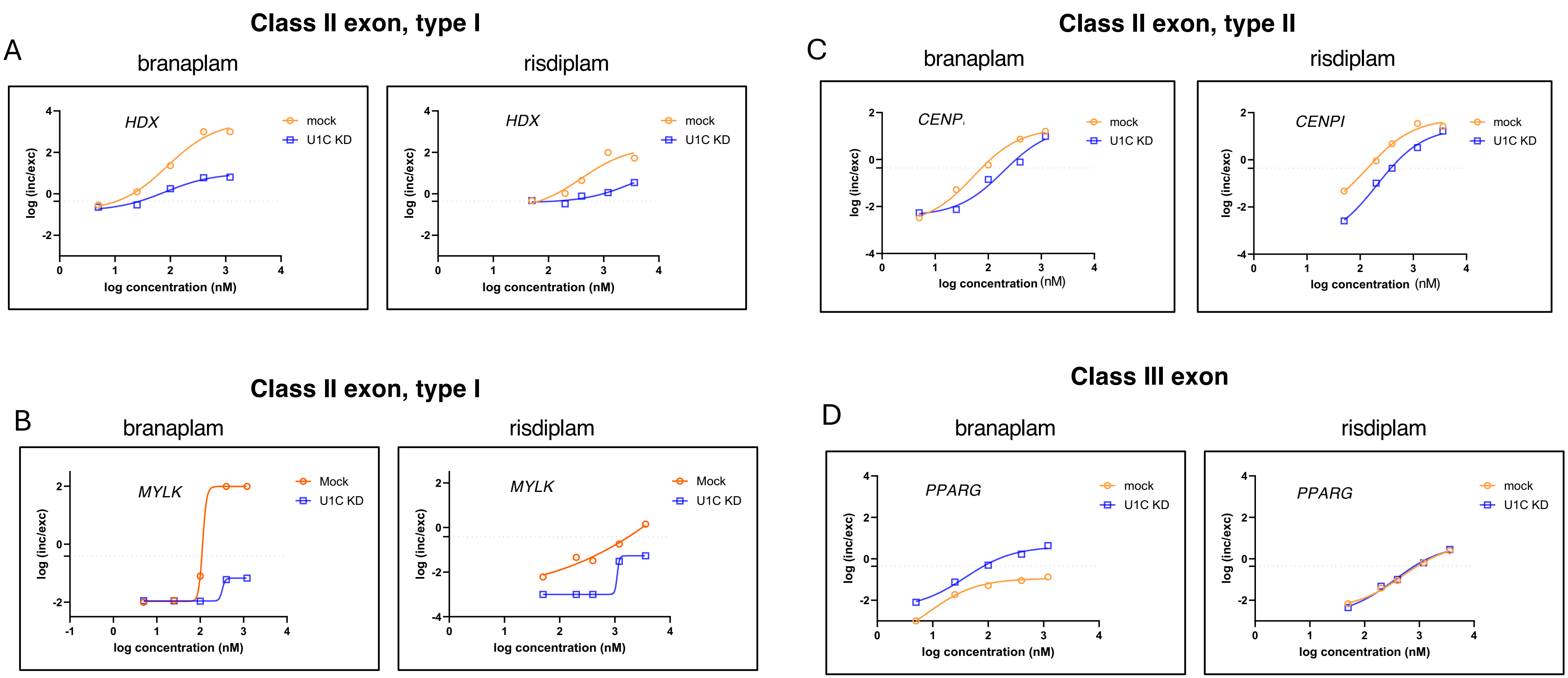

Supplementary Fig. 11. Additional examples demonstrate the effect of U1C KD on iExon inclusion mediated by branaplam or risdiplam.

A-C. Plot of PSI of a representative class II induced by splicing modulator compound treatment where the Emax inclusion of these exons by compound is reduced in U1C knockdown. Dash lines in all panels represent PSI30. All logarithms in this figure are to base 10.

D. Plot of PSI of a representative class III exon induced by splicing modulator compound treatment, which represents a very low inclusion exon where splicing inclusion by branaplam is greatly enhance by U1C knockdown.

Supplementary Table 1. Primers used in this study.

Name	Sequence	Notes
U1A-gRNA1F	CACCGAAGGTGCTACTTCTTGGCAA	For generating pX330-U1A-sgRNA1
U1A-gRNA1R	AAACTTGCCAAGAAGTAGCACCTTC	For generating pX330-U1A-sgRNA1
U1A-gRNA2F	CACCGGGGCAGGCATGGGGGGAAA	For generating pX330-U1A-sgRNA2
U1A-gRNA2R	AAACTTTCCCCCCATGCCTGCCCC	For generating pX330-U1A-sgRNA2
U1A DF	G*G*AGTTTGACAATGAGGTACAGGCAGGGGCAGCTCGCGATGCCCTGCAGGGCTTTAAGATCACGCAGAACAACGCCATGAAGATCTCCTTTGCCAAGAAGGGAGGCGGTTACCCATAC	N* means Phosphorothioated bases, forward primer used in PCR to gnerate linear doner DNA fragments
U1A DR	A*G*AAGGCCCCCAAGGGGGGACTTACCTTCAGGGGGCTGAGCCAAGGGGGAAAGGGGTGGCCCCAGAACAGGGGAAGGGGCAGGCATGGGGGGAAAGGTGGTCGACTGATCATAATCAGC	N* means Phosphorothioated bases, reverse primer used in PCR to gnerate linear doner DNA fragments
U1AendF	CTTCAAGGAGGTCCGTCTG	Forward PCR primer for screening genome-edited clones
U1A3UTRR	CCAAACCTCAAAAAGCCACTT	Used with U1AendF to screen for the presence of the wild-type allele
HTPR	CTTCATCGTGTTGCGCAA	Used with U1AendF to screen for incorporation of the HTP-T2A-neo cassette
U1C_Ex3F2	TATTATCAGAAATGGATGGAAGAGC	
U1C_Ex4R2	GTAGGAGGTATCTTTCCTTGTTG	
PPIA_qPCR_F	GACTGAGTGGTTGGATGGCAAGC	
PPIA_qPCR_R	TCGAGTTGTCCACAGTCAGCAATG	
ALCAM_F	GCACAGCAGAAAACCAACTGGAG	Expected skip product is 154bp and inclusion product is 193bp
ALCAM_R	AGCCAGTAGACGACACCAGCAAC	
SNHG5_F	GGAAAATCGCTTGGTTAAAACCTGACAC	Expected skip product is 106bp and inclusion product is 187bp
SNHG5_R	AGAAATCGTTGCAACTTTGGAACTTCC	
c12orf4_F	GCCCAGGACTTCGGAACTA	Expected skip product is 175bp and inclusion product is 310bp
c12orf4_R	TGACTGGCATTCTCTTGAACA	
DENND4A_F	TGACTGGCATTCTCTTGAACA	Expected skip product is 115bp and inclusion product is 142bp
DENND4A_R	CCATACTTTTCAACAGTTCCTGGT	
