# Supplementary material for: The U1 snRNP protein U1C and Helix H of U1 snRNA are critical for small molecule splicing modulator function
Source: Nat Commun. 2026 Jul 16;17:8837. doi: 10.1038/s41467-026-74765-8 (PMC13500670; doi:10.1038/s41467-026-74765-8)
Supplement: Supplementary file 3 [file 41467_2026_74765_MOESM6_ESM.pdf]

nature portfolio

Peer Review File

The U1 snRNP protein U1C and Helix H of U1 snRNA are critical for small molecule splicing modulator function

Corresponding Author: Dr Rui Zhao

This file contains all reviewer reports in order by version, followed by all author rebuttals in order by version.

Attachments originally included by the reviewers as part of their assessment can be found at the end of this file.

Version 0:

Reviewer comments:

Reviewer #1

(Remarks to the Author)
In this paper Kuang et al present an analysis of the effects of branaplam and risdiplam on binding of U1 snRNP complexes to model 5' splice sites. They also test several mutant splice site and U1 snRNA combinations (in reconstituted snRNPs) with and without branaplam and analogs. In vivo experiments documenting the effect of U1C protein depletion or mutant U1 snRNA expression on splicing in the presence and absence of compounds are presented. This is important work – these small molecules have interesting activity profiles on splicing events associated with specific 5' splice site subclasses, and appear to be able to access the brain in living mammals, offering an advantage to oligonucleotide medicines which must be injected across the blood-brain barrier. Their mechanism of action on 5' splice site selection is not well understood and this work begins to address some of the details of how the compounds influence 5'ss binding to U1 snRNP and affect splicing in vivo. The experiments are challenging but are well done, however I have some suggestions about how to make the presentation clearer and more accessible to general readers.
Major comments:
Comment 1. I found it striking that the Kd for both the 5'ss oligos in the presence of branaplam was not substantially different in the native vs reconstituted U1 snRNP, but the binding in the absence of drug was much worse for the reconstituted snRNP (Fig 1). This and the statements about the extra U1C added to counteract weak association of U1C with the snRNP made me wonder if branaplam stabilizes the binding of U1C with the snRNP in the absence of a 5'ss, or if it prevents dissociation of U1C when a 5'ss is bound. Measuring this would require different binding experiments but could reveal something more about the mechanism. It seems the difference in behavior between the reconstituted and native snRNPs could be discussed a bit more – the other difference to think about is the absence of pseudoUs and 2'-O-methyl modifications in the reconstituted snRNP. Although the pseudoUs interact with the intronic side of the 5' splice site away from the nucleotides of interest, there could be effects on U1C binding.
Comment 2. The similar order of binding effects and splicing effects for the series of branaplam derivatives is interesting – does it tell us anything about which functional groups of the most potent family member contribute to the effects? Are we only learning here that the binding assay recapitulates the activity profile? A diagram of the core molecule with the differences between it and the derivatives described would help a discussion about this.
Comment 3. In Fig 4 we are seeing the failure of every tested mutation of both 5'ss oligos to bind – the tested mutations focus on the exon nucleotides that distinguish the affected 5'ss from those not activated by branaplam in vivo. A wild type control would be nice to show here – as would a diagram showing the pairing that is thought to be necessary – visually capturing the location of the mutations in the pairing scheme will help readers identify impacts of the mutations on the pairing.
Comment 4. Figure 5 could also be more accessible along the lines noted above for Fig4. For example, reading this: "(C119U/U120A/G121U/C122A)" for most people will not result in an understanding of the changes in U1 snRNA folding that the authors are hoping for from their readers. At least an image of U1 is presented, perhaps adding numbers and/or an enlarged inset of the Helix H region with mutations indicated will help. A technical concern related to comment 1 here is how these mutations impact the binding of U1C. Do the mutant U1 snRNAs reconstitute equivalently to wild type? The compensatory double mutant that restores Helix H is almost back to normal, so it is likely the conclusions about the requirements for this helix are correct.
Comment 5. The experiment in Figure 6 is more complex than it might appear at first. One question is why these measurements were normalized to the mock transfection (no U1 gene transfected) rather than to the GA (otherwise wild

type) transfection. If we want to know how the mutations in Helix H affect the function of the U1-GA snRNP, then it seems the activity of the (otherwise wild type) U1-GA snRNP should be the comparator and should end up in the denominator. I'm also wondering if the expression levels of the U1-GA and its variants are the same or not – it seems possible that a mutation could cause U1 to fold differently and that might impact its accumulation and its activity independently of a specific Helix H defect. Are the splicing changes caused by the wild type U1-GA dependent on the level of expression of the transfected U1? What level of incoming U1 expression is required to see these changes over and above a large background of endogenous U1?
Comment 5. Figure 7 is an important experiment that might be presented earlier as it emphasizes the focus on the U1C protein's contributions and the lack of major contributions by U1A and U1 70K. Panel A is very helpful, however I wanted to rotate it upwards to look at the region from underneath and perhaps in a space filling rendition to see if there were nooks that might accommodate bulged nucleotides or drugs.
Comment 6. The interpretations to explain the splicing changes supported by the compounds in vivo under U1C depletion are complex (Fig 8). Regarding the exons whose inclusion is activated by U1C depletion, the idea that too tight binding of U1 snRNP to the 5' splice site as inhibitory is supported by findings in yeast engineered to have hyperstabilized U1-5'ss interactions. Work from Staley and T.H. Chang showed that mutations that weaken U1C binding (and other elements of the U1 snRNP) can rescue the hyperstabilized phenotype. PRP28 helicase is responsible for releasing U1 so that U6 can bind as the spliceosome assembly pathway progresses. In terms of the behavior of the different exon classes it may be important to remember that U1C depletion is not complete – it seems possible that what the drug is doing is advantaging the target exon in a competition for U1C-containing U1 snRNPs. In other words rather than replacing the contribution of U1C or accommodating its absence, the drug is preferentially enhancing the competitive ability of the unusual 5' splice site to access U1C-containing U1 snRNPs. It would be worth knowing in this regard whether U1C depletion reduces or destabilizes endogenous U1 snRNP. Is there a functional class of U1 snRNPs that lacks U1C and if so is it servicing a large number of canonical 5' splice sites? Or are all U1 snRNPs still bound to U1C but the population of U1 snRNP is depleted? Either way, competition for U1 could be exacerbated, enhancing the effect of the drug.

Reviewer #2

(Remarks to the Author)
Referee report for "The U1 snRNP protein U1C and Helix H of U1 snRNA 1 are critical for small molecule splicing modulator function"
By Kuang et al.
October 29th, 2025

Summary:
In this manuscript, Kuang et al. investigate the molecular mechanism by which the splicing modulator branaplam and its analogs enhance recognition of weak, non-canonical 5' splice sites (5'ss) by U1 snRNP. They assess this through measurements of U1 snRNP-5'ss interaction strengths and transcriptomic analyses under modified U1 snRNP conditions. The authors demonstrate that the secondary structure - but not the nucleotide sequence - of U1 snRNA Helix H, as well as residues 1-61 including the ZnF domain of U1C, are critical for drug function. These effects, observed in *in vitro* binding experiments, are further examined by transcriptomic profiling in cells overexpressing U1 Helix H mutants or with U1C knockdown.

This study provides valuable biochemical and transcriptomic data elucidating the effect of branaplam on 5'ss-U1 snRNP interactions, complementing previous work highlighting the role of U1C in branaplam functon(White et al., *Nat. Comm.* 2024, PMID: 39389991). However, several *in vitro* and *in vivo* experiments lack appropriate controls or contain interpretational issues that may affect the validity of the conclusions.

Major comments:

1.
In Figure 8 and Supplementary Figures 8 and 9B, the authors claim that "reduction in U1C leads to a significant decrease in the potency of both branaplam and risdiplam," as evidenced by the need for higher drug concentrations to achieve the same PSI under U1C knockdown (KD). However, this observation can alternatively be explained by differences in basal splicing efficiencies in the absence of drug treatment. These differences are not visible on gels or cannot be accurately measured by RNA-seq when PSI values approach 0 or 100. When these differences are maintained at higher drug concentrations in the log-scale of inclusion over exclusion ratios, it cannot be claimed that the drugs require U1C to activate target 5'ss.

To convincingly demonstrate that 5'ss sensitivity to the drugs decreases under U1C KD, exons with intermediate PSI values (5-95) at lowest drug concentration in both control and KD conditions should be analyzed. Drug responses should be plotted on a log scale of inclusion/exclusion ratios; only when the control curve is steeper or the KD condition turn up at higher concentrations in this log scale, can it be concluded that U1C reduction decreases drug potency. Currently, all examples in Fig. 8B-D and Supplementary Fig. 8 show saturated PSI values at 0 or 100 in at least one KD condition.

For RNA-seq data, inclusion/exclusion ratios can be calculated from PSI using the formula inc/exc = PSI / (100 - PSI), provided there are sufficient reads for the less abundant isoform. As an alternative to gel visualization, RT-qPCR measurements using isoform-specific primers could be used to quantify inclusion/exclusion ratios, where differences in cycles numbers are directly differences in inc/exc ratios in log2 scale.

2.
The authors claim that U1 snRNA Helix H is a key determinant of GA/GU 5'ss recognition, using U1 snRNA mutants where positions 9 and 10 are fixed as UC (instead of CU), and Helix H is altered. This experiment indicates that Helix H mutants are less functional, likely due to interference with 5'ss recognition by endogenous U1 snRNA. However, this was tested outside the context of small-molecule treatment, and while U1 snRNP binding to 5'ss is necessary, it is not sufficient for splicing, thus the *in vitro* data involving both branaplam and Helix H are insufficient to justify the title claim that "Helix H of U1 snRNA is critical for small molecule splicing modulator function".

If Helix H mutants are generally less functional U1 snRNAs, the statement that "U1 snRNP recognition of GA/GU 5'ss requires a specific structural configuration of Helix H" (lines 335-336) is misleading. It would be more accurate to state that "Helix H mutant U1 snRNA is less functional", possibly by disrupting downstream splicing steps.

To demonstrate that Helix H specifically affects GA/GU 5'ss recognition, additional U1 snRNA mutants (other than UC at positions 9 and 10) should be expressed, and their effects tested against corresponding 5'ss (e.g., AC U1 snRNA against GU/GU 5'ss, GA U1 snRNA against UC/GU 5'ss). If only GA/GU-specific effects are observed, the authors could justifiably claim specificity. RNA-seq is not essential for this, but the target cassette exons should have baseline PSI values between 5-95 or be measured by RT-qPCR, as noted in comment 1.

Minor comments:

- Possible citation management error at line 61: should the reference be PMID: 39223315?
- Line 215: "-1G and -2A" may be a typo; should this be "-1A and -2G" or "-2G and -1A"?
- The terms *in vitro* and *in vivo* should be italicized consistently throughout the manuscript.

Reviewer #3

(Remarks to the Author)
This manuscript investigates the important mechanisms by which small molecules used as therapeutic agents modulate splicing by enhancing U1 snRNP binding to suboptimal 5’ss. Authors mainly use an elegant in vitro biochemical approach to measure binding of various substrates to U1 snRNPs isolated in two different ways, either reconstituted or pulled down (native). While the experiments appear to be well conducted and the data is clear (with few exceptions outlined below), the main problem resides in the fact that this work does NOT carry virtually anything new and important, because most results were already shown in other systems. The binding of branaplam to 5’ss, its sequence specificity, and dependency on U1C were described previously. Furthermore, the branaplam dependency on helix H is not surprising as this helix is essential for the proper structure of the U1 snRNA, and it would have been more convincing to introduce mutations in all other helices and loops to show some specificity of the branaplam dependency. The final RNAseq experiments for the U1C knockdown are interesting yet not analysed in depth, and this by itself does not bring enough novelty to the table. Based on this and on the specific comments below, this referee thinks that this manuscript better belongs to another journal, because the largely confirmatory results are still important to be reported to the community.

Major specific comments:

1. The fact that the in vitro binding system did not work for risdiplam is of concern, any potential reasons why?
2. The in vitro data on Helix H in Figure 5 is not as clear as described in the text. In panel 1B, the U1 concentration lines did not show the curved shape for binding, probably because this U1 carries too many mutations and it might not be held together long enough. Panel 1C shows some of the correct shape but not as clear as 1D, which carries the only convincing data in this figure.
3. For the U1C knockdown plus RNAseq experiment, it would have been good to rescue cells with siRNA-resistant U1C, wild type or with domain mutations containing or lacking the zinc finger, to show some specificity.

Version 1:

Reviewer comments:

Reviewer #1

(Remarks to the Author)
I have read the authors' responses to the original reviews and have re-read the modified manuscript. I think the authors have done a meticulous job of addressing all the reviewers concerns and that the paper is ready for publication without further delay. The discoveries here are important for understanding the mechanisms and potential for intervention in splicing control by therapeutic small molecules and significantly extend what we know. Congratulations to the authors.

Reviewer #2

(Remarks to the Author)
The reviewer appreciates the attempt to improve the manuscript. However, there are several critical misinterpretations in the *in vivo* data which are the basis of the claim of this manuscript.

Response to the rebuttal for major comment 1.
When Response Fig. 2 is plotted by a log-log plot, instead of the linear-log and log-linear plots that the authors provided, U1C knockdown results in a downward shift rather than a right shift, with saturation at inclusion/exclusion ~ 1.0 (attached figure). The reason this type of experiment requires log-log plot is as follows.
Since I agree with the authors' statement "The primary function of U1 snRNP is to recognize the 5' splice site (5'ss) and to our knowledge there is no evidence that U1 snRNP plays a direct role in downstream splicing steps", we can assume that the ratio of U1 binding to the 5'ss in interest becomes the inclusion/exclusion rate. Also, we assume that the drug binds only to the state in which the U1 snRNP and the 5'ss form a duplex, and drug binding is independent of events outside the 5'ss, and vice versa, which means the free energies of U1 binding and drug binding are additive.

The probability of any microstates in thermodynamic equilibrium is given by the Boltzmann distribution (equation 1) where G_i is the Gibbs free energy of state *i*, and *Z* is the sum over all states. The model assigns Gibbs free energies to each state as follows:
State 1: nothing bound: G_1 = 0 (reference)
State 2: U1 bound, no drug: G_2 = ΔG_U1
State 3: U1 bound + drug bound: G_3 = ΔG_U1 + ΔG_drug
When we convert free energies to Boltzmann weights e^(-G/RT):
State 1: e^0 = 1
State 2: e^(-ΔG_U1/RT) -> defined as *S*
State 3: e^(-(ΔG_U1 + ΔG_drug)/RT) = e^(-ΔG_U1/RT) × e^(-ΔG_drug/RT) = *S* × e^(-ΔG_drug/RT)
We define *E* as the sum of Boltzmann weights for all U1-bound states, normalized by the Boltzmann weight of the drug-free U1-bound state alone (equation 2). As *S* cancels in this definition, *E* depends on the drug-binding free energy ΔG_drug, and is independent of other context outside the 5'ss. At this point, we do not know if ΔG_drug is influenced by the presence of U1C.
The total Boltzmann weight of all U1-bound states is shown as the sum of State 2 and 3: *S* × *E* (equation 3). The partition function *Z* is the sum of Boltzmann weights over all states (equation 4). Thus, the probability that U1 is bound is the total weight of U1-bound states divided by the partition function: P(U1 bound) = (*S* × *E*) / (1+ *S* × *E*) and PSI = 100 × P(U1 bound). This gives (inclusion/exclusion) = *S* × *E* which leads to log (inclusion/exclusion) = log(*S*) + log(*E*), where a vertical shift of the curves can be attributed to differences only in *S* and not *E* when the y-axis is plotted on log scale. This is easier to see when the x-axis is also log-scale (bottom-right figure of Response Fig. 2. is already a downshift until saturation). This reasoning also applies to analyses using EC-dPSI30: as this value is influenced by both *S* and *E*, it is not an appropriate value to compare.
For the case of *PDXDC1*, there is a downward shift except at high concentrations, and at high concentrations, we can assume that the inclusion rate is saturated at PSI = 50 due to contextual effects. If the loss of U1C abrogates drug binding, the saturation PSI should also be lower when U1C is knocked down.

I stated that "we do not know if ΔG_drug is influenced by the presence of U1C", so to show that ΔG_drug is influenced by U1C, it must be demonstrated that *E* changes upon U1C knockdown. Taking *PDXDC1* for example, assuming that the new exon is GRCh38 chr16:15,009,499-15,009,561 with a 5'ss of AAGA|GTAAGA, when the difference in Ct values between inclusion-specific and exclusion-specific primers differs between 0 drug and 100 nM drug using RT-qPCR, it can be demonstrated that U1C changes not only *S* but also *E*. The primer combinations would be inclusion: [TCCCAGCATCAGATGTCTTCA-GGCAGTCTTCCTCGCTCTAT] and exclusion: [TCCCAGCATCAGATGGATGT-GGCAGTCTTCCTCGCTCTAT]. Another reason to carry out RT-qPCR is because the readouts are more stable and quantitative than gel imaging, which can involve a large degree of arbitrary variation in measurement.
However, when the raw Ct values are above approximately 30, the measurements will be outside the dynamic range and will not be a reliable measure (it is unreliable in gel-based RT-PCR in any case). It is highly possible that it happens with U1C KO with 0 drug. In that case, choosing another exon with a higher inc/exc at 0 drug with U1C KD or concentrating poly(A)+ RNA would be effective in lowering the overall Ct. Another way to find a candidate is to search for an exon from the RNA-seq data which the PSI is 5-95 in both control and KD conditions at no drug, and the difference in log(PSI/(100-PSI)) between mock and U1C KD changes upon drug treatment.

If U1C knockdown only influences *S* but not ΔG_drug and *E*, this can explain the different "classes" of exons. Class II, which has "the complete loss of compound response in some iExons upon U1C knockdown", is when the inclusion/exclusion of U1C KD is so low at any concentration that a 10-fold difference of inclusion/exclusion might be a PSI difference of 0.0001 against 0.001 which would be invisible on gel. Class III is the opposite when it is low in U1C control. This also raises a concern for the in vitro experiments: it suggests a possibility - although it is difficult to prove - that the differences in polarization values in the in vitro experiments are also attributable to different basal binding strengths that cannot be distinguished with the dynamic range of the experiment, as the values are too small at low drug concentrations and cannot measure the fold-change accurately.

Response to the rebuttal for major comment 2.
The reviewer appreciates the effort by the authors for additional RNA-seq experiments. I agree with the statement "this requirement is likely not specific to GA/GU but applies more broadly to 5' ss in general". This shows that in vivo, "effect of Helix H on 5' ss recognition is fundamental to U1 function", but not in the context of drug treatment. Although it has been shown in vitro that Helix H is critical for the enhancing of interaction by the drug, it is important to note that this has not been demonstrated in vivo. It must be noted that line 416 "This unexpected result indicates that the precise conformation of Helix H and consequently the positioning of the 5' end of U1 snRNA is crucial for the activity of branaplam" can be said for in vitro

experiments, but not for in vivo experiments.

Minor additional comments (which are also written on the guidelines for authors):
The authors should provide methods for gel RT-PCR and its quantification, including the primers used for each gene. Uncropped gel images and raw values for plots should be provided in the source data file. The area of the gel image used for quantification also should be provided.

The authors must have provided an access key to the high-throughput sequencing data uploaded to GEO database, next to the accession number.

All instances of *SMN2* in "SMN2 exon 7" should be italicized, as it is a gene name.

Version 2:

Reviewer comments:

Reviewer #2

(Remarks to the Author)
The reviewer appreciates the authors' additional analyses and clarifications in the revised manuscript. The responses to the previous concerns are largely satisfactory, and the manuscript is now considered suitable for publication, pending the minor revisions listed below.

Additional minor points:
- Gene names that refer to transcripts should be written in italics in both in the main text and the figures. This applies to all gene names appearing in the RNA-seq analysis.
- Please indicate the base of the logarithm in Figure 8 and Supplementary Fig. 10, unless the base is e.
- In the paragraph starting in line 533: the LUC7L proteins mentioned appear to refer to human proteins, therefore, their names should be written entirely in upper case.
- Please double check whether Supplementary Figure 11 is cited in the main text. If it is currently cited only outside the main text, please add an appropriate citation in the relevant section of the main text.

Open Access This Peer Review File is licensed under a Creative Commons Attribution 4.0 International License, which permits use, sharing, adaptation, distribution and reproduction in any medium or format, as long as you give appropriate credit to the original author(s) and the source, provide a link to the Creative Commons license, and indicate if changes were made.
In cases where reviewers are anonymous, credit should be given to 'Anonymous Referee' and the source.
The images or other third party material in this Peer Review File are included in the article's Creative Commons license, unless indicated otherwise in a credit line to the material. If material is not included in the article's Creative Commons license and your intended use is not permitted by statutory regulation or exceeds the permitted use, you will need to obtain permission directly from the copyright holder.
To view a copy of this license, visit https://creativecommons.org/licenses/by/4.0/

We appreciate the reviewers' constructive feedback on our manuscript. Below we have outlined our responses (in blue) to all comments and recommendations (in black).

Reviewer 1.
Major comments
1. I found it striking that the Kd for both the 5'ss oligos in the presence of branaplam was not substantially different in the native vs reconstituted U1 snRNP, but the binding in the absence of drug was much worse for the reconstituted snRNP (Fig 1). This and the statements about the extra U1C added to counteract weak association of U1C with the snRNP made me wonder if branaplam stabilizes the binding of U1C with the snRNP in the absence of a 5'ss, or if it prevents dissociation of U1C when a 5'ss is bound. Measuring this would require different binding experiments but could reveal something more about the mechanism. It seems the difference in behavior between the reconstituted and native snRNPs could be discussed a bit more – the other difference to think about is the absence of pseudoUs and 2'-O-methyl modifications in the reconstituted snRNP. Although the pseudoUs interact with the intronic side of the 5' splice site away from the nucleotides of interest, there could be effects on U1C binding.

To answer this question, we have labeled the N-terminus of U1C with a Cy5 fluorophore and titrated in increasing concentrations of U1 snRNP (without U1C) in FP experiment to evaluate the binding affinity between U1C and the rest of U1 snRNP. We found that branaplam does not affect the binding affinity of U1C to the rest of U1 snRNP in the absence of 5' ss. In the presence of the 5' ss, branaplam did increase the binding affinity between U1C and the rest of U1 snRNP (i.e., the compound prevents dissociation of U1C in the presence of 5' ss). This observation reveals that branaplam improves recognition of weak 5' ss through two synergistic mechanisms, by strengthening the binding between U1 snRNP and the weak 5' ss and enhancing the association of U1C with the U1 snRNP. We have added these data in Fig. 5E-F and additional discussion related to this observation (lines 432-437).

We have added a discussion of the different behavior between reconstituted and native U1 snRNP in binding the 5' ss (lines 351-356).

2. The similar order of binding effects and splicing effects for the series of branaplam derivatives is interesting – does it tell us anything about which functional groups of the most potent family member contribute to the effects? Are we only learning here that the binding assay recapitulates the activity profile? A diagram of the core molecule with the differences between it and the derivatives described would help a discussion about this.

These data indeed reveal functional groups important for the compound's effect. We have added a figure (Supplementary Fig. 3B) and discussion about this (lines 358-361).

3. In Fig 4 we are seeing the failure of every tested mutation of both 5'ss oligos to bind – the tested mutations focus on the exon nucleotides that distinguish the affected 5'ss from those not activated by branaplam in vivo. A wild type control would be nice to show here – as would a diagram showing the pairing that is thought to be necessary – visually capturing the location of the mutations in the pairing scheme will help readers identify impacts of the mutations on the pairing.

We have added the positive controls for these experiments in Supplementary Fig. 4. We also added the pairing between the U1 snRNA and 5' ss under each mutant in Fig. 4.

4. Figure 5 could also be more accessible along the lines noted above for Fig4. For example, reading this: "(C119U/U120A/G121U/C122A)" for most people will not result in an understanding of the changes in U1 snRNA folding that the authors are hoping for from their readers. At least an image of U1 is presented, perhaps adding numbers and/or an enlarged inset of the Helix H region with mutations indicated will help. A technical concern related to comment 1 here is how these mutations impact the binding of U1C. Do the mutant U1 snRNAs reconstitute equivalently to wild type? The compensatory double mutant that restores Helix H is almost back to normal, so it is likely the conclusions about the requirements for this helix are correct.

We have added an enlarged view of helix H with nucleotide numbers and mutated nucleotides indicated in Fig. 6A. Additional base pairing between the U1 variants and noncanonical 5' ss is shown in Supplemental Figure 6A and mutations to Helix H in Supplemental 6B.

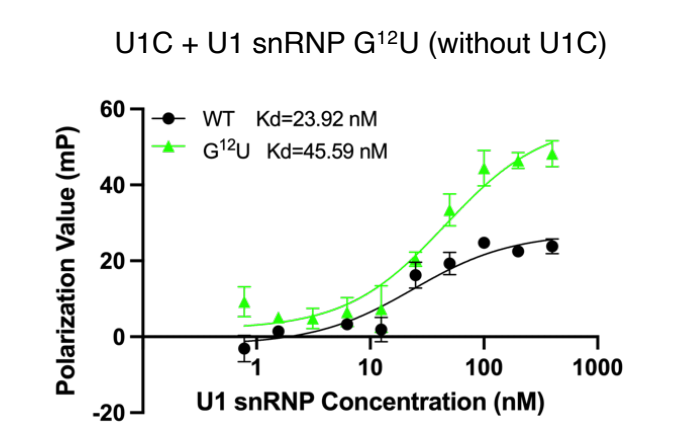

Response Fig. 1. Helix H mutant slightly reduces the binding affinity between U1 snRNP and U1C compared to the WT.

We have evaluated the binding affinity between fluorescently labeled U1C and U1 snRNP $G^{12}U$ (without U1C) using FP experiments. U1 snRNP $G^{12}U$ binds U1C with a slightly weaker binding affinity compared to the WT U1 snRNP (see Response Fig. 1). U1 snRNP $G^{12}U$ reconstitutes similarly to U1 snRNP WT (see Supplementary Fig. 1C) and still binds to the 5' ss (Fig. 6C).

5. The experiment in Figure 6 is more complex than it might appear at first. One question is why these measurements were normalized to the mock transfection (no U1 gene transfected) rather than to the GA (otherwise wild type) transfection. If we want to know how the mutations in Helix H affect the function of the U1-GA snRNP, then it seems the activity of the (otherwise wild type) U1-GA snRNP should be the comparator and should end up in the denominator. I'm also wondering if the expression levels of the U1-GA and its variants are the same or not – it seems possible that a mutation could cause U1 to fold differently and that might impact its accumulation and its activity independently of a specific Helix H defect. Are the splicing changes caused by the wild type U1-GA dependent on the level of expression of the transfected U1? What level of incoming U1 expression is required to see these changes over and above a large background of endogenous U1?

We appreciate the reviewer's suggestion that all splicing changes shown in Figure 6 (new Fig. 7 in the revised manuscript) could be normalized to the WT U1-GA variant to emphasize the changes caused directly by the mutations in Helix H. However, we feel it is also important to first highlight the exons whose inclusion is induced by base pairing between the nucleotide substitutions made in the U1 snRNA sequence and nucleotides at -2 and -1 position in the 5' ss. We therefore decided to include both modes of normalization in the revised manuscript. As reviewer #2 suggested, we repeated this experiment with two addition U1 variants that enrich for inclusion of distinct sets of exons with either a UU or UG at the 5' ss. Normalizing to the mock

transfected or newly included U1-WT transfected has no effect on the conclusion from the figure or the discussion and allows for a single analysis workflow for the multiple U1 variants with and without helix H mutations. The revised Fig. 7A plot the PSI of each splicing event being considered in both the U1-GA transfected and each of the helix H variants tested normalized to the mock transfection, and Fig. 7B and C reflect normalization to U1-GA as the reviewer suggested. Supplementary figure 6C and D present similar normalization results for the new U1-UU and U1-UG data. We have modified the results and discussion section to discuss these changes to the analysis and figures.

While expression levels of U1 snRNA variants could be contributing to the differences in splicing observed between U1 variants with WT or mutated helix H, we feel the new *in vitro* U1 snRNP reconstitution data showing similar assembly of single mutants, mutants with compensatory substitutes, and even the tetra mutant (Supplementary Fig. 1C) suggest *in vivo* assembly is likely not impaired. Direct measurement of the expression level of U1 snRNA variants can be challenging due to the proximity of the mutations at nucleotides 9 and 10 to the 5' end of the snRNA (RT reaction often does not reach the 5' end and can be further hindered by the presence of the 5' cap). We have included a comment on this being a possible interpretation, along with the above discussion, in lines 396-405.

5 [sic]. Figure 7 is an important experiment that might be presented earlier as it emphasizes the focus on the U1C protein's contributions and the lack of major contributions by U1A and U1 70K. Panel A is very helpful, however I wanted to rotate it upwards to look at the region from underneath and perhaps in a space filling rendition to see if there were nooks that might accommodate bulged nucleotides or drugs.

We appreciate the reviewer's suggestion. We initially thought keeping the U1C *in vitro* and *in vivo* data together would provide strong impact but agree the impact of figure 7 could be presented earlier and then later supported with the *in vivo* splicing changes. This change was made.

We have generated a view that is rotated 90 degrees along the horizontal axis to look at the complex from underneath and a corresponding surface representation with a potential cavity near the 5' ss highlighted (see revised Fig. 5A).

6 [sic]. The interpretations to explain the splicing changes supported by the compounds in vivo under U1C depletion are complex (Fig 8). Regarding the exons whose inclusion is activated by U1C depletion, the idea that too tight binding of U1 snRNP to the 5' splice site as inhibitory is supported by findings in yeast engineered to have hyperstabilized U1-5'ss interactions. Work from Staley and T.H. Chang showed that mutations that weaken U1C binding (and other elements of the U1 snRNP) can rescue the hyperstabilized phenotype. PRP28 helicase is responsible for releasing U1 so that U6 can bind as the spliceosome assembly pathway progresses. In terms of the behavior of the different exon classes it may be important to remember that U1C depletion is not complete – it seems possible that what the drug is doing is advantaging the target exon in a competition for U1C-containing U1 snRNPs. In other words rather than replacing the contribution of U1C or accommodating its absence, the drug is preferentially enhancing the competitive ability of the unusual 5' splice site to access U1C-

containing U1 snRNPs. It would be worth knowing in this regard whether U1C depletion reduces or destabilizes endogenous U1 snRNP. Is there a functional class of U1 snRNPs that lacks U1C and if so is it servicing a large number of canonical 5' splice sites? Or are all U1 snRNPs still bound to U1C but the population of U1 snRNP is depleted? Either way, competition for U1 could be exacerbated, enhancing the effect of the drug.

We appreciate the reviewer's thoughtful comments and have included the supporting evidence in yeast and corresponding references in the discussion (lines 466-469).

We thank the reviewer for pointing out the possibility that the compound is helping the weak 5' ss to compete for U1C-containing U1 snRNP. This indeed seems to be the case as we have shown that in the presence of branaplam, U1C binds to U1 snRNP with higher affinity (see new Fig. 5F), potentially because the compound promotes the formation of the U1 snRNA and 5' ss duplex that favors U1C binding. We believe these observations provide new insight into the mechanism of branaplam, which acts by strengthening the binding between U1 snRNA and the weak 5' ss and by enhancing the association of U1C with the U1 snRNP. These effects may synergistically reinforce one another. We have added these discussions in the revised manuscript (lines 432-437).

To answer the reviewer's additional questions:
(1) We have knocked down U1C in HEK293 cells and pulled down U1 snRNP using an anti-U1A antibody. We showed that the anti-U1A antibody pulled down similar quantities of Sm proteins (using an anti-SmB antibody, as a surrogate for U1 snRNP integrity), suggesting that U1C KD does not reduce or destabilize endogenous U1 snRNP. We added the anti-SmB probing data in Supplementary Fig. 7B. In addition, this is also consistent with our *in vitro* observations that U1 snRNP without U1C can be reconstituted similarly as the full U1 snRNP, with similar yield, stability, and biochemical behaviors (see new Supplementary Fig. 1C).

(2) We have reconstituted U1 snRNP without U1C which has similar yield and behavior during reconstitution and purification, indicating that the lack of U1C does not affect the stability of the rest of U1 snRNP (see new Supplementary Fig. 1C). We also showed that U1 snRNP without U1C still binds the 5' ss but with lower affinity (Fig. 5C). In U1C KD, it preferentially affects non-canonical 5' ss (Supplementary Fig. 8C), suggesting that U1 without U1C is functional and can probably still service canonical site.

(3) Given that under the conditions and timing used in this knockdown study, U1C KD does not seem to affect the levels of other U1 snRNP proteins (we have evaluated SmB and U1A in Supplementary Fig. 7B), thus it is unlikely that the overall population of U1 snRNP is reduced with U1C KD.

Reviewer 2.
Major comments
1. In Figure 8 and Supplementary Figures 8 and 9B, the authors claim that "reduction in U1C leads to a significant decrease in the potency of both branaplam and risdiplam," as evidenced by the need for higher drug concentrations to achieve the same PSI under U1C knockdown (KD). However, this observation can alternatively be explained by differences in basal splicing

efficiencies in the absence of drug treatment. These differences are not visible on gels or cannot be accurately measured by RNA-seq when PSI values approach 0 or 100. When these differences are maintained at higher drug concentrations in the log-scale of inclusion over exclusion ratios, it cannot be claimed that the drugs require U1C to activate target 5'ss.

To convincingly demonstrate that 5'ss sensitivity to the drugs decreases under U1C KD, exons with intermediate PSI values (5-95) at lowest drug concentration in both control and KD conditions should be analyzed. Drug responses should be plotted on a log scale of inclusion/exclusion ratios; only when the control curve is steeper or the KD condition turn up at higher concentrations in this log scale, can it be concluded that U1C reduction decreases drug potency. Currently, all examples in Fig. 8B-D and Supplementary Fig. 8 show saturated PSI values at 0 or 100 in at least one KD condition.

For RNA-seq data, inclusion/exclusion ratios can be calculated from PSI using the formula inc/exc = PSI / (100 - PSI), provided there are sufficient reads for the less abundant isoform. As an alternative to gel visualization, RT-qPCR measurements using isoform-specific primers could be used to quantify inclusion/exclusion ratios, where differences in cycles numbers are directly differences in inc/exc ratios in log2 scale.

To simplify our response to the reviewer's comment, we will only use branaplam as an example but the same analyses and conclusions apply to risdiplam. We appreciate the reviewer's concern that since the events shown in Fig. 8B have starting PSI value close to 0, it is difficult to know if U1C KD would have reduced PSI under basal or low compound concentration, and if the right shift in the dose response curve solely reflects reduced U1C level rather than reduced compound sensitivity. However, if the reduced splicing were solely due to U1C reduction, we would expect a similar degree of reduction across all compound concentrations. This is clearly not the case as the dose response curve in the mock knockdown is steeper than the curve with U1C KD. As seen in Response Fig. 2, this conclusion remains unchanged whether we plot the dose response curve using PSI or inclusion/exclusion ration in linear or log scale as the reviewer suggested (although plotting PSI or PSI/(100-PSI) on either a linear or log scale may offer certain visual benefits, mathematically, we do not expect these choices to alter the conclusion).

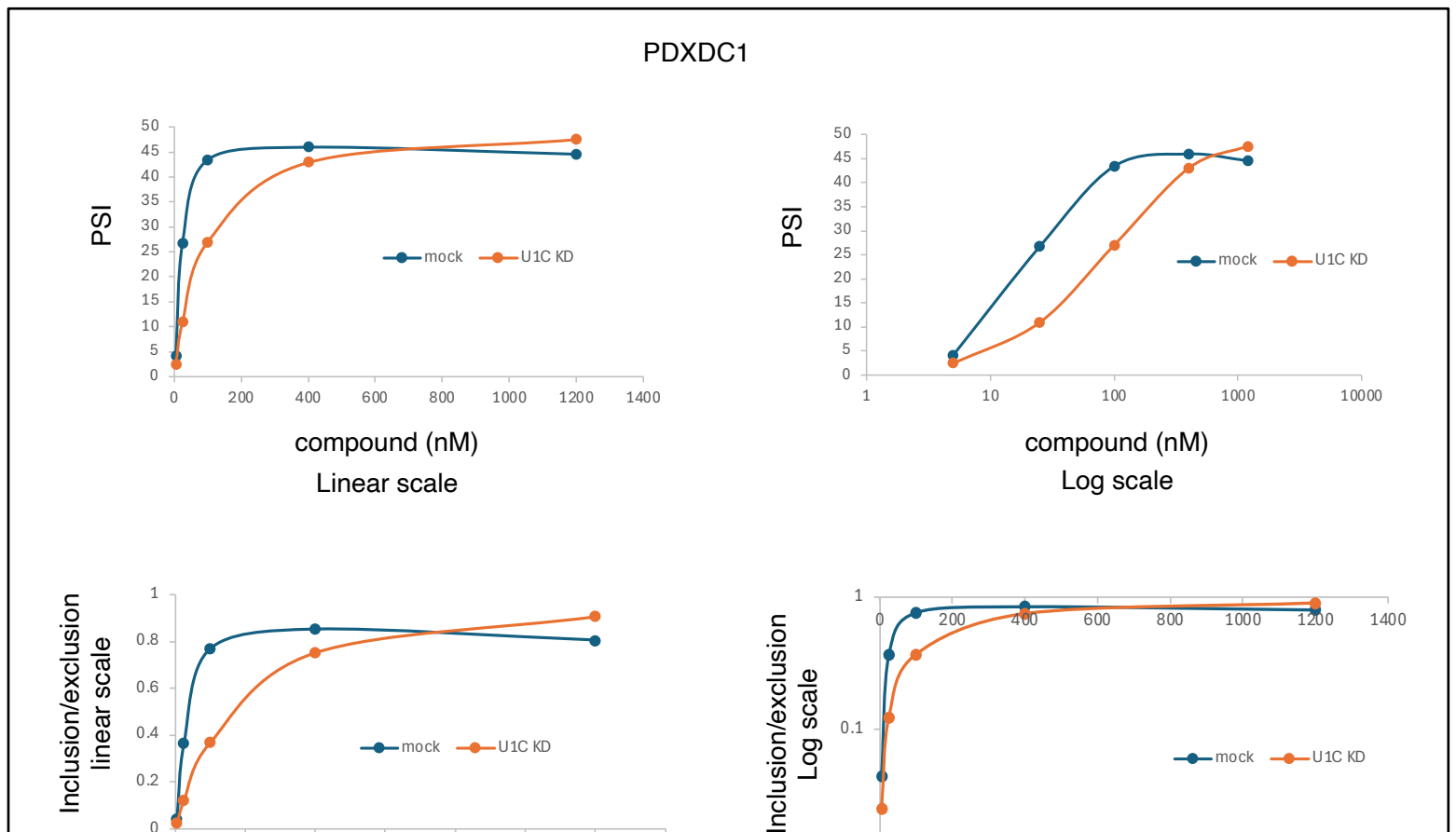

Response Fig. 2. Compound dose response curves for PDXDC1 plotted using either PSI or inclusion/exclusion ratio in either linear or log scale do not change the conclusion that the compound response is attenuated in U1C KD (the dose response curve in the mock knockdown is significantly steeper than that in the U1C KD condition).

To further support this point, we selected iExons with intermediate PSI values (9-95) at the lowest compound concentration and repeated the above analyses (Response Fig. 3), as the

reviewer suggested. For example, CMSS1 has a PSI of 6 at 5nM branaplam (most compound responsive iExons have noncanonical 5' ss and low PSI in the absence of compound). It shows a robust response to branaplam in the mock knockdown, but this response is markedly reduced upon U1C KD. Consistent with this, the dose response curve in the mock condition is steeper than that in U1C KD, whether plotted using PSI or the inclusion/exclusion ratio. Another example is WNK4 which has a PSI of 6.7 at 5nM branaplam. It exhibits a strong response to branaplam in the mock knockdown but this response is abolished upon U1C KD as shown by the dose response curve plotted using either PSI or inclusion/exclusion ratio. We have used these and similar exons as better examples illustrating the requirement for U1C for the effect of branaplam in the revised Fig. 8F and Supplementary Fig. 10A.

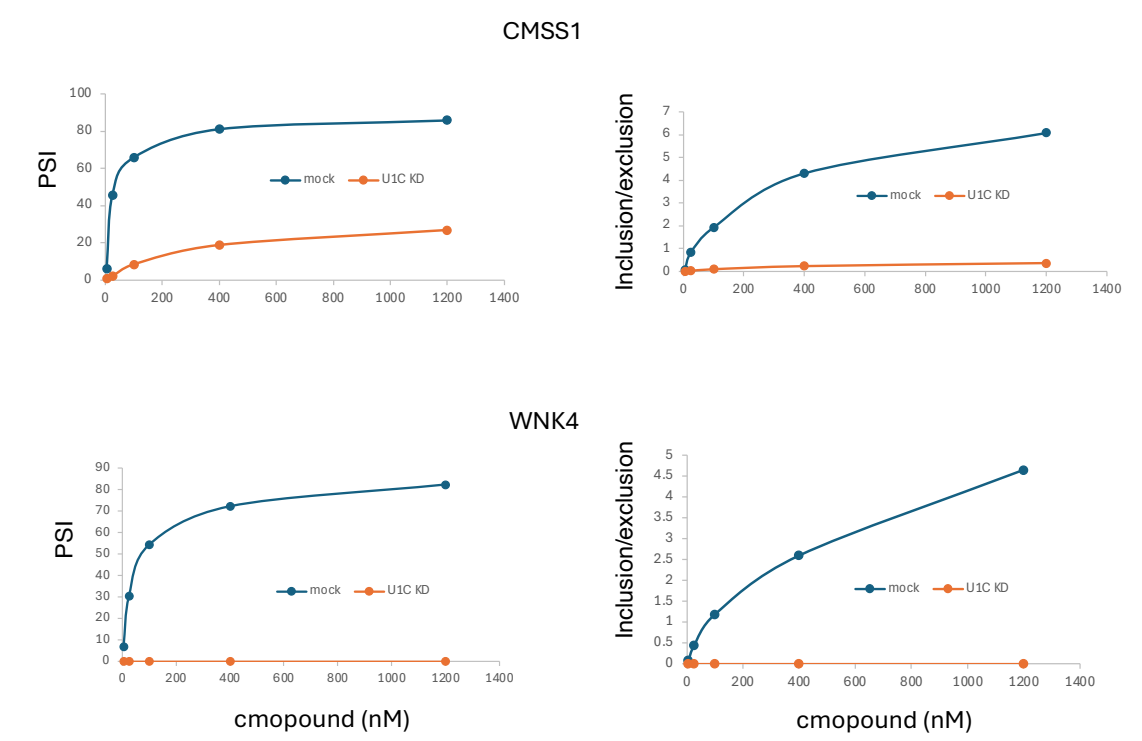

Response Fig. 3. Selected iExons with PSI > 5 at 5nM branaplam show a strong response to branaplam in the mock knockdown but dramatically reduced or abolished response in U1C KD, as demonstrated by compound dose response curves generated using either PSI or inclusion/exclusion ratios.

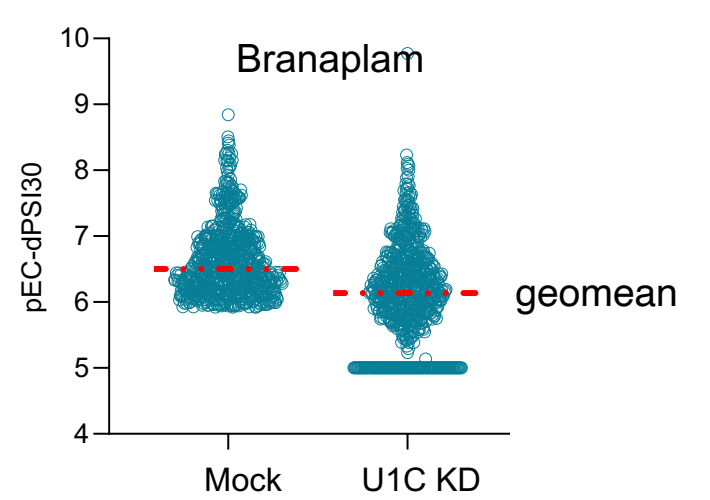

Response Fig. 4 (new Fig. 8C). Plot of pEC_dPSI30 (-log transformed compound concentration when dPSI achieves 30%) for all GA/GU iExons. The red dashed lines represent the geomean of each treatment. See revised manuscript for details.

Taking the reviewer's comment into consideration, we have altered our analysis to provide a more comprehensive view of the sequencing data, including iExons with baseline PSI values across a wide range (0-70). The analysis we utilized was a calculation of EC-dPSI30, the effective concentration of compound that achieves a splicing change of 30% deltaPSI ($PSI_{compound}$ – PSI_{DMSO}), which is based on curve fitting to the dose response effect for each iExon. Selection of 30% is based on the reviewer's concern about saturation of the splicing effect, as this is well within the exponential phase of the dose response for the majority of splicing events induced by splicing modulators. The transcriptome-wide effect of U1C knockdown on the potency of branaplam and risdiplam is presented in the new Figure 8A-C. In Fig. 8C (also Response Fig. 4), we observe a modest 3-fold reduction in branaplam potency (a decrease of 0.5 in the geomean of -Log(EC-dPSI30)) for the majority of iExons. This general effect is potentially caused by U1C reduction, which is known to reduce 5' ss binding by U1 snRNP.

In addition to the global effect potentially caused by U1C reduction, Fig. 8D (also Response Fig. 5) highlights that

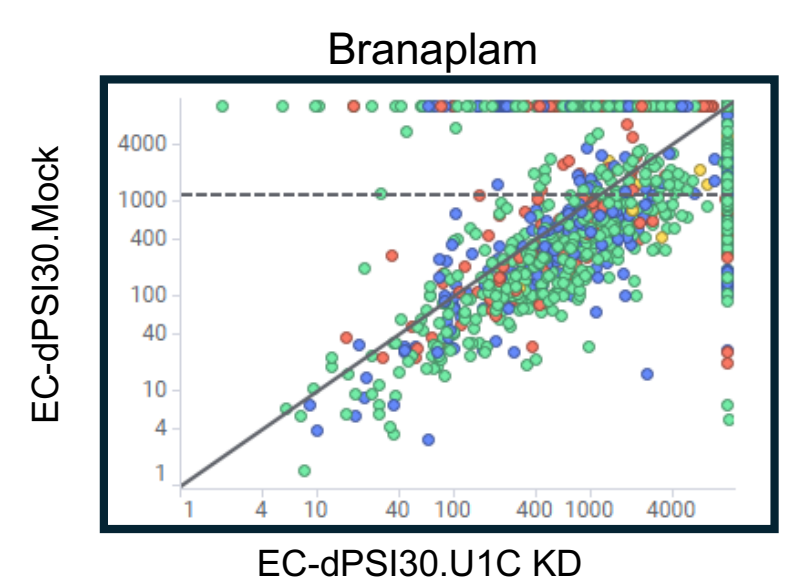

Response Fig. 5 (new Fig. 8D). Scatter plot of the nanomolar concentrations for the EC-dPSI30 for DMSO (y-axis) versus U1CKD (x-axis) in branaplam treated cells. The no effect line (y=x) is plotted; events where compound potency is attenuated by U1C reduction are shifted to the right of the no effect line and those that are enhanced shift to the left of the no effect line. Events that are inactive for either treatment are seen along the top and right side of the plot (no calculable EC-dPSI30). See revised manuscript for details.

there is a target specific effect on potency as demonstrated by a wide range of effects for compound induced splicing. These include numerous examples of exons where splicing is: I) completely abolished; II) splicing is decreased greater than 0.5 log and III) exons where inclusion is enhanced when U1C is knocked down. Together, these observations suggest that, for many splicing modulator target exons, the exon response to the compound upon reduction of U1C is differentially influenced by U1C availability, in addition to a global effect of U1C reduction on splicing. In particular, the complete loss of compound response in the subclass I exons strongly indicates a requirement of U1C in mediating the compound's effect.

2. The authors claim that U1 snRNA Helix H is a key determinant of GA/GU 5'ss recognition, using U1 snRNA mutants where positions 9 and 10 are fixed as UC (instead of CU), and Helix H is altered. This experiment indicates that Helix H mutants are less functional, likely due to interference with 5'ss recognition by endogenous U1 snRNA. However, this was tested outside the context of small-molecule treatment, and while U1 snRNP binding to 5'ss is necessary, it is not sufficient for splicing, thus the in vitro data involving both branaplam and Helix H are insufficient to justify the title claim that "Helix H of U1 snRNA is critical for small molecule splicing modulator function".

Helix H mutants were generated on the background of the UC mutation on nucleotides 9 and 10 of U1 snRNA (designated as U1-GA). We are only monitoring the splicing of a set of exons with a GA/GU 5' ss, which are recognized by U1-GA but not recognized by endogenous U1 snRNA (as demonstrated by baseline PSI values close to 0 for the majority of compound induced GA/GU exons in Fig. 8A) and are therefore not subjected to interference from endogenous U1 snRNA.

Our *in vitro* data clearly demonstrate that helix H is critical for branaplam's effect on enhancing the interaction between U1 snRNP and 5' ss (Fig. 6). Since the majority of exons induced by small molecule splicing modulators are unannotated, intron-derived sequences which are not efficiently recognized by U1 snRNP and poorly spliced, we believe the observed contributions of Helix H to U1 snRNA binding to GA/GU 5'ss *in vitro* strongly suggests that Helix H would also be critical for compound-mediated iExon recognition by U1 snRNP and iExon inclusion in vivo. We have decided to keep the current title due to word limit constraints, but we have clarified in the Discussion that this claim is derived from *in vitro* data.

We agree that the *in vivo* experiment was performed outside the context of small molecule treatment. However, the findings that Helix H is critical for the interaction between compound, U1 snRNP, and the 5'ss *in vitro* was surprising and prompted us to investigate the role of Helix H in U1 function *in vivo* where modification of specific nucleotides in Helix H, demonstrated its role and requirement for 5'ss recognition and splicing. This fundamental insight on the role of Helix H in U1snRNP function in splicing is further supported by our evaluation of additional U1 variants suggested by the reviewer (see below).

If Helix H mutants are generally less functional U1 snRNAs, the statement that "U1 snRNP recognition of GA/GU 5'ss requires a specific structural configuration of Helix H" (lines 335-336) is misleading. It would be more accurate to state that "Helix H mutant U1 snRNA is less functional", possibly by disrupting downstream splicing steps.

The primary function of U1 snRNP is to recognize the 5' ss and to our knowledge there is no evidence that U1 snRNP plays a **direct** role in downstream splicing steps. While failure to recognize the 5' ss will clearly affect downstream splicing, this effect is indirect. A less functional U1 snRNA results from its inability to recognize the 5' ss. Therefore, we believe it is justified to state that "U1 snRNP recognition of 5'ss requires a specific structural configuration of Helix H." We have removed the reference to GA/GU 5'ss, as this requirement is likely not specific to GA/GU but applies more broadly to 5' ss in general.

To demonstrate that Helix H specifically affects GA/GU 5'ss recognition, additional U1 snRNA mutants (other than UC at positions 9 and 10) should be expressed, and their effects tested against corresponding 5'ss (e.g., AC U1 snRNA against GU/GU 5'ss, GA U1 snRNA against UC/GU 5'ss). If only GA/GU-specific effects are observed, the authors could justifiably claim specificity. RNA-seq is not essential for this, but the target cassette exons should have baseline PSI values between 5-95 or be measured by RT-qPCR, as noted in comment 1.

We appreciate the reviewer's comment. When we stated Helix H affects GA/GU 5' ss recognition, we specifically refer to the context of the U1-GA snRNA variant. We do see how our language could be unclear. By restricting the analysis to the set of exons induced by U1-GA variant that have complementary bases at the -2/-1 5' ss position, we believe the analysis of GA/GU 5' ss allows us to more precisely identify the effect of the helix H mutations. We did not mean to suggest this effect is specific to GA/GU 5' ss. Since the substitution of nucleotides 9 and 10 in U1 snRNA from CU to UC alter recognition of 5'SS with -2/-1 nucleotides AG to GA, the structure at the 5' ss is likely forming a fully complementary duplex in both examples. To show this effect of Helix H on 5' ss recognition is fundamental to U1 function, we performed RNA sequencing on two additional sets of U1 variants. We engineered U1 variants to enhance splicing of UG/GU 5' ss which represents a single nucleotide difference from WT U1. For our second set we engineered a U1 variant to enhance splicing of UU/GU 5'SS (see new supplemental figure 6). RNA sequencing of these U1 variants without Helix H mutations enhanced splicing of 5' ss with UG and UU at the -2/-1 position as expected. Furthermore, the pattern of decreased splicing when combined with Helix H mutations that were observed with the U1-GA variant was also observed in U1-UG and U1-UU variants. We added these new data in the revised manuscript (lines 273-280).

We feel this finding strengthens our suggestion that Helix H stability and sequence identity could influence 5'SS recognition. This could be analogous to the model proposed for the stability of U6-ISL playing an important role in mediating the transition between first and second step conformations of the catalytic core (Eysmont et al., Mol Cell 2019). In our example of U1 Helix H, we hypothesize that alterations to the sequence identity or stability of Helix H, may promote the transition of U1 snRNP interacting with non-canonical 5' ss to a more stable conformation that allows for splicing to proceed more efficiently.

Minor comments:
- Possible citation management error at line 61: should the reference be PMID: 39223315?
The reviewer is correct and we have corrected this error.

- Line 215: "-1G and -2A" may be a typo; should this be "-1A and -2G" or "-2G and -1A"?
The reviewer is correct and we have corrected this typo.

- The terms in vitro and in vivo should be italicized consistently throughout the manuscript.
We have corrected this throughout the manuscript.

Reviewer 3.
While the experiments appear to be well conducted and the data is clear (with few exceptions outlined below), the main problem resides in the fact that this work does NOT carry virtually anything new and important, because most results were already shown in other systems. The binding of branaplam to 5'ss, its sequence specificity, and dependency on U1C were described previously. Furthermore, the branaplam dependency on helix H is not surprising as this helix is essential for the proper structure of the U1 snRNA, and it would have been more convincing to introduce mutations in all other helices and loops to show some specificity of the branaplam dependency. The final RNAseq experiments for the U1C knockdown are interesting yet not analysed in depth, and this by itself does not bring enough novelty to the table. Based on this and on the specific comments below, this referee thinks that this manuscript better belongs to another journal, because the largely confirmatory results are still important to be reported to the community.

Below, we clarify the novelty and significance of our work and have also made these points clearer in the revised manuscript.

1. The binding of branaplam to U1 snRNP and the 5' ss, its sequence specificity, and its dependency on U1C have been described previously either in cellular reporter assays or using a minimal U1 snRNP. This minimal complex contains truncated Sm proteins, lacks U1A, includes only a 61-residue fragment of U1C (out of 159 residues), a 59-residue fragment of U1-70K (out of 437 residues), and a 60-nucleotide U1 snRNA (out of 164 nt) with an engineered kissing loop. As a result, it is difficult to determine whether observations made using this minimal U1 snRNP (such as the dependency on U1C) reflects physiological behavior or raise from the absence of many native U1 snRNP components. Using a near native U1 snRNP (differing only by a short C-terminal truncation of SmB and removal of the C-terminal RS domain of U1-70K, with all other proteins and U1 snRNA being full-length), we unambiguously demonstrated the dependency of branaplam activity on U1C. That this finding is consistent with previous observations made using minimal U1 snRNP should not detract from its significance in demonstrating this dependency in a near native setting.

2. We provide the first evidence that Helix H plays an important role in both 5' ss recognition and the mechanism of action of small molecule splicing modulators. Helix H is formed by base pairing between nucleotides 119-122 and 12-15, whereas the remaining 11 nucleotides at the 5' end of U1 snRNA upstream of helix H are flexible. Disruption of helix H is therefore expected to untether or relax the 5' end of U1 snRNA without compromising the overall structural stability of the U1 snRNP (especially a single nucleotide substitution at the end of helix H (G12U)). Indeed, U1 snRNP with helix H mutant reconstitutes similarly to the WT U1 snRNP (see new Supplementary Fig. 1C) and retains the ability to bind the HTT 5' ss (Fig. 6B and C). However, even with a single nucleotide change at the end of helix H, branaplam can no longer enhance the

interaction between U1 snRNP and weak 5' ss. This unexpected result indicates that the precise conformation of helix H and consequently the positioning of the 5' end of U1 snRNA is crucial for the activity of branaplam.

3. During manuscript revision and in response to Reviewer 1's suggestion, we found that branaplam not only enhances the interaction between U1 snRNP (likely via U1 snRNA) and weak 5' ss, but also strengthens the interaction between U1C and U1 snRNP (likely facilitated by stabilization of the U1 snRNA and 5' ss duplex). These results reveal an unexpected dual mechanism of action for branaplam: it both reinforces binding between U1 snRNA and weak 5' ss and enhances the U1C association with the U1 snRNP, with these effects potentially acting synergistically.

4. Incorporating *in vivo* studies allowed us to validate our *in vitro* observations and uncover unexpected insights into the function of U1C by including transcriptome-wide analysis of endogenous substrates of splicing modifiers not represented in focused *in vitro* studies. These data enabled us to reveal a previously unrecognized role of U1C in suppressing the splicing of a subset of introns as well as in suppressing the effect of the compounds.

5. This led us to propose a model in which U1C stabilizes specific conformations at the 5' ss/U1 snRNA interface that either facilitate or hinder compound binding and effect on splicing, in contrast to the earlier model that relies on direct interaction between U1C and branaplam (PMID 39389991). These findings have broad implications for the development of other splicing modulators and RNA-targeting therapeutics in general.

6. We discovered a clear distinction between two classes of splicing modulators (risdiplam and branaplam) currently in clinical use or clinical trial for the treatment of SMA and HD. These differences will spur further studies by us and others to advance our understanding of the mechanisms by which these classes of splicing modulators enhance 5' ss recognition and usage.

Specific comments:
1. The fact that the in vitro binding system did not work for risdiplam is of concern, any potential reasons why?

The most likely reason is that the action of risdiplam requires additional cellular factors. One possible candidate is the Luc7L family of alternative splicing factors (Luc7L, L2, and L3). These proteins are homologs of the yeast Luc7 protein which binds to the U1 snRNA and 5' ss RNA duplex on the opposite side of U1C and stabilizes the duplex together with U1C. We discussed this possible reason in the last paragraph of discussion.

2. The in vitro data on Helix H in Figure 5 is not as clear as described in the text. In panel 1B, the U1 concentration lines did not show the curved shape for binding, probably because this U1 carries too many mutations and it might not be held together long enough. Panel 1C shows some of the correct shape but not as clear as 1D, which carries the only convincing data in this figure.

We have replotted Fig. 5B-D (new Fig. 6B-D, no change in the data) to make the binding curves more obvious visually. We have also added data in Supplementary Fig. 1C showing that U1

snRNP carrying the four Helix H mutants can be reconstituted and behaves similarly to the WT during purification.

3. For the U1C knockdown plus RNAseq experiment, it would have been good to rescue cells with siRNA-resistant U1C, wild type or with domain mutations containing or lacking the zinc finger, to show some specificity.

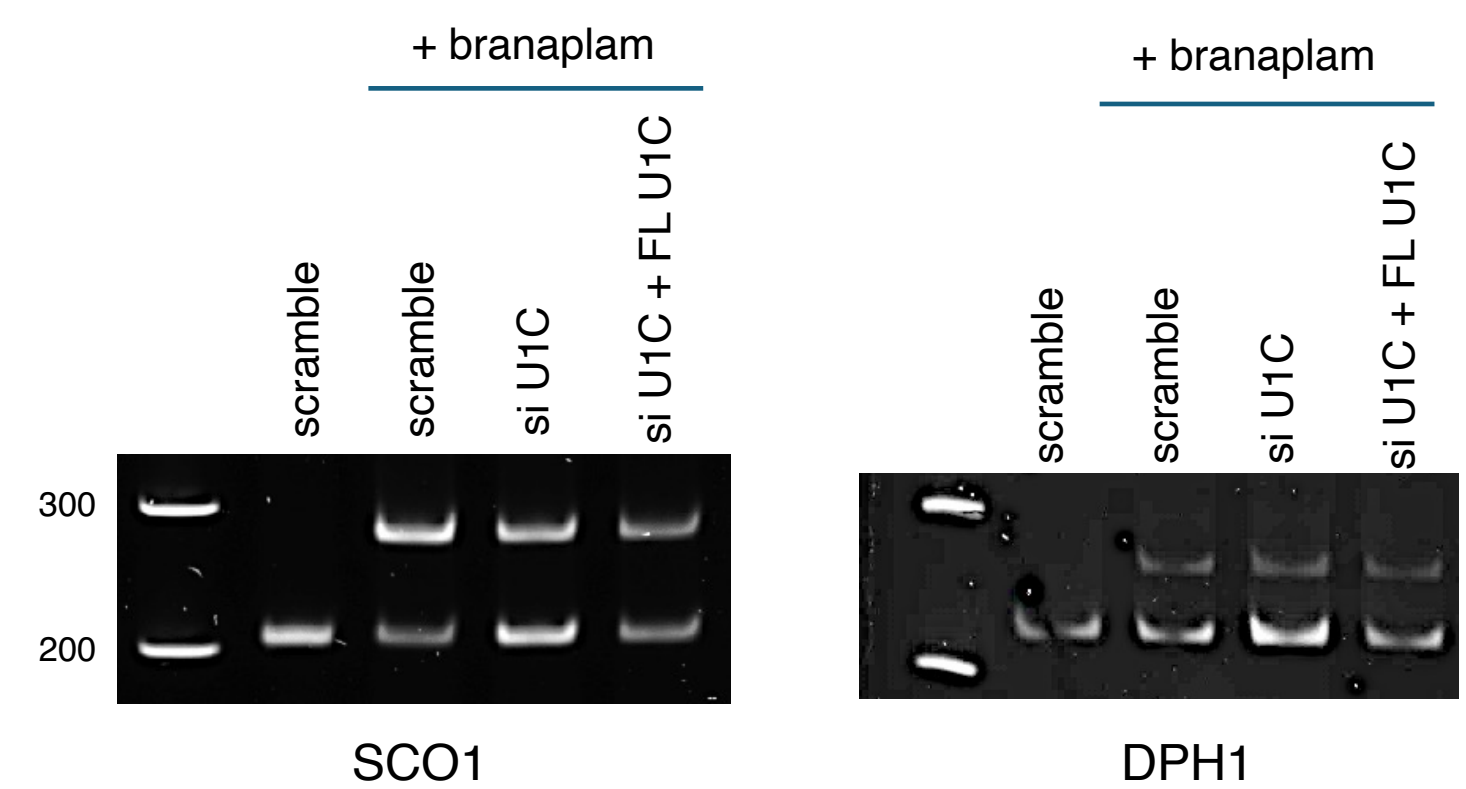

Response Fig. 6. RT-PCR showing two representative iExons that were included upon branaplam treatment with scrambled siRNA. The inclusion was reduced upon U1C KD and was rescued by FL U1C addback.

Following the reviewer's suggestion, we have knocked down U1C in HEK293 cells and added back FL U1C. The NTD of U1C that contains the ZnF domain and the CTD did not express in HEK293 cells so we were not able to add back these domains. We showed that branaplam induces the inclusion of representative iExons in scramble siRNA treated cells (Response Fig. 6). U1C KD reduced this inclusion and FL U1C addback rescues the inclusion, indicating that the loss of inclusion is specific to U1C KD. Note that the effects of KD and addback are subtle which is often the case for RT-PCR analyses of small changes in splicing isoform distribution. We have added these data to Supplementary Fig. 11.

We appreciate reviewer 2's effort to help us further improve our manuscript. Below are our responses (in blue) to all comments and recommendations (in black).

In our effort to address the reviewer's original concerns in the first revision, we performed additional analysis that demonstrated the U1C KD leads to a marked decrease in the potency of the branaplam and risdiplam (Figure 8, A-E). Based on the latest comments by reviewer 2, we agree that the change in potency alone does not immediately reveal the underlying mechanism responsible for the loss of potency. Upon their recommendation, we plotted log10(inclusion/exclusion) versus log10(drug concentration) for all exons affected by U1C KD in the absence or presence of splicing modulator.

This analysis revealed two response types for **Class II** exons for which U1C KD reduces compound potency. The first type of response (type I) is represented by numerous class II exons, where exon inclusion is at a similar level in U1C KD relative to mock in the lowest compound concentration, but is unable to achieve maximal exon inclusion (Emax) even at the highest concentration (Fig. 8F-G, Supplementary Fig. 10A, B**)**. This type of exons demonstrated increased log (inc/exc) differences between the mock and U1C KD samples as the compound concentration increases, satisfying the criteria suggested by reviewer 2 for demonstrating U1C loss reduces the compound activity. Since the binding site for splicing modulators is likely the complex between the U1 snRNA and the pre-mRNA 5'ss, this data suggests that U1C is critical to stabilize a specific structure(s) that is conducive to compound binding and ultimately splicing of noncanonical GA/gu exons.

The second type of response (type II) is represented in exons where the dose response curve is right shifted in compound upon U1C KD (Fig 8H; Supplementary Fig. 10C). A majority of these exons achieve a similar Emax, either at the same concentration seen in mock or for some requiring a higher dose. This type of response suggests that loss of U1C alters the binding affinity of the small molecule splicing modulators for their target binding site, the U1 snRNA-5'ss interface, such that the compound can only achieve its full potential of exon inclusion at high concentrations. For both types of response there are examples where both branaplam and risdiplam are equally effected and ones where the response is compound specific (Fig. 8H; Supplementary Fig. 10C). The differences between branaplam and risdiplam response to U1C KD suggest these are compound specific effect independent of U1C KD.

Reviewer 2 also suggested quantifying inclusion and exclusion using RT-qPCR (Ct values) rather than gel image or using PSI/(100-PSI) from RNAseq data. We did not utilize gel image analysis for any of our conclusions, so this is not relevant to our interpretations. In addition, as we generated a very robust RNAseq dataset, we feel that the new curve analysis that was performed across thousands of exons, is more quantitative and comprehensive than a single or several RT-qPCR evaluations of randomly selected exon events. In addition, this allowed us to examine numerous exons where RT-qPCR would be unable to determine changes in quantity due to low abundance, something RNAseq is able to capture due to its high sensitivity.

Additional comments from Reviewer 2:
For the case of *PDXDC1*, there is a downward shift except at high concentrations, and at high concentrations, we can assume that the inclusion rate is saturated at PSI = 50 due to contextual

effects. If the loss of U1C abrogates drug binding, the saturation PSI should also be lower when U1C is knocked down.

For PDXDC1, the saturation rate does appear to be ~ PSI=50. The baseline PSI in DMSO for mock and U1C KD is within 2X (0.27 vs 0.18 PSI), and at 5nM (the lowest compound concentration), there is a similar level of inclusion (2.9 vs 2.6 PSI). As dose increases, there is a clear separation of branaplam-inclusion response resulting in a right shifted curve. Ultimately both treatments reach similar saturation at the highest dose. Please note that the log (inc/exc) vs log [compound] curve plotted by the reviewer differs from ours because they accidentally applied an additional logarithmic transformation to the log (inc/exc) value and used that for the y axis. As described above, this represents a type II curve response and suggest that loss of U1C is likely impacting binding affinity of the splicing modulator. Risdiplam treatment shows a similar right shifted curve, but with a lower overall saturation at PSI=40. It is clear that compound reaches saturation, but since the dose response did not cover the full range of effect for risdiplam we selected a different representative exon event in Figure 8H.

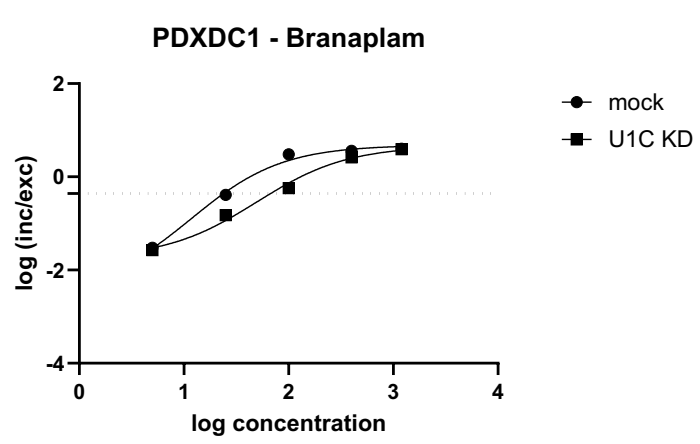

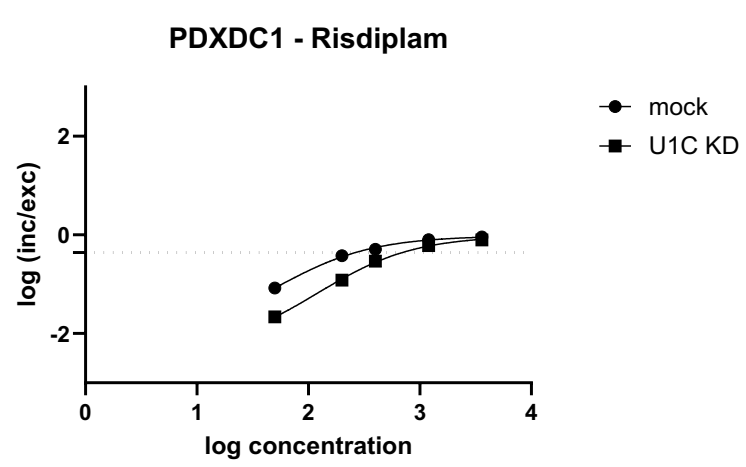

If U1C knockdown only influences *S* but not ΔG_drug and *E*, this can explain the different "classes" of exons. Class II, which has "the complete loss of compound response in some iExons upon U1C knockdown", is when the inclusion/exclusion of U1C KD is so low at any concentration that a 10-fold difference of inclusion/exclusion might be a PSI difference of 0.0001 against 0.001 which would be invisible on gel

This is relevant to our Class II exons and we have selected 3 representatives where this is not a confounding issue. All baseline values in DMSO are reliable and similar to each other (within 3-fold or less).
PSI baselines for Figure 8 graphs are:

GENE	MOCK	U1CKD
PDE7A	4.3	12.71
GXYLT1	0	0.03
MAN1A2	0.117	0.09

Response to the rebuttal for major comment 2.
The reviewer appreciates the effort by the authors for additional RNA-seq experiments. I agree with the statement "this requirement is likely not specific to GA/GU but applies more broadly to 5' ss in general". This shows that in vivo, "effect of Helix H on 5' ss recognition is fundamental to U1 function", but not in the context of drug treatment. Although it has been shown in vitro that Helix H is critical for the enhancing of interaction by the drug, it is important to note that this has not been demonstrated in vivo. It must be noted that line 416 "This unexpected result

indicates that the precise conformation of Helix H and consequently the positioning of the 5' end of U1 snRNA is crucial for the activity of branaplam" can be said for in vitro experiments, but not for in vivo experiments.

We have revised the text to more clearly state which conclusions are drawn from *in vitro* versus *in vivo* analyses.

Minor additional comments (which are also written on the guidelines for authors):
The authors should provide methods for gel RT-PCR and its quantification, including the primers used for each gene. Uncropped gel images and raw values for plots should be provided in the source data file. The area of the gel image used for quantification also should be provided.

We have incorporated this information in the Materials and Methods section and provided the gel image, quantification area, and raw values in source data file.

The authors must have provided an access key to the high-throughput sequencing data uploaded to GEO database, next to the accession number.

The access key onmxeciatxujpcb.

All instances of *SMN2* in "SMN2 exon 7" should be italicized, as it is a gene name.

Corrected.

Additional minor points from Reviewer 2:
- Gene names that refer to transcripts should be written in italics in both in the main text and the figures. This applies to all gene names appearing in the RNA-seq analysis.
- Please indicate the base of the logarithm in Figure 8 and Supplementary Fig. 10, unless the base is e.
- In the paragraph star2ng in line 533: the LUC7L proteins men2oned appear to refer to human proteins, therefore, their names should be written entirely in upper case.
- Please double check whether Supplementary Figure 11 is cited in the main text. If it is currently cited only outside the main text, please add an appropriate cita2on in the relevant sec2on of the main text.

We thank the reviewer for identifying these errors and have corrected all of them.

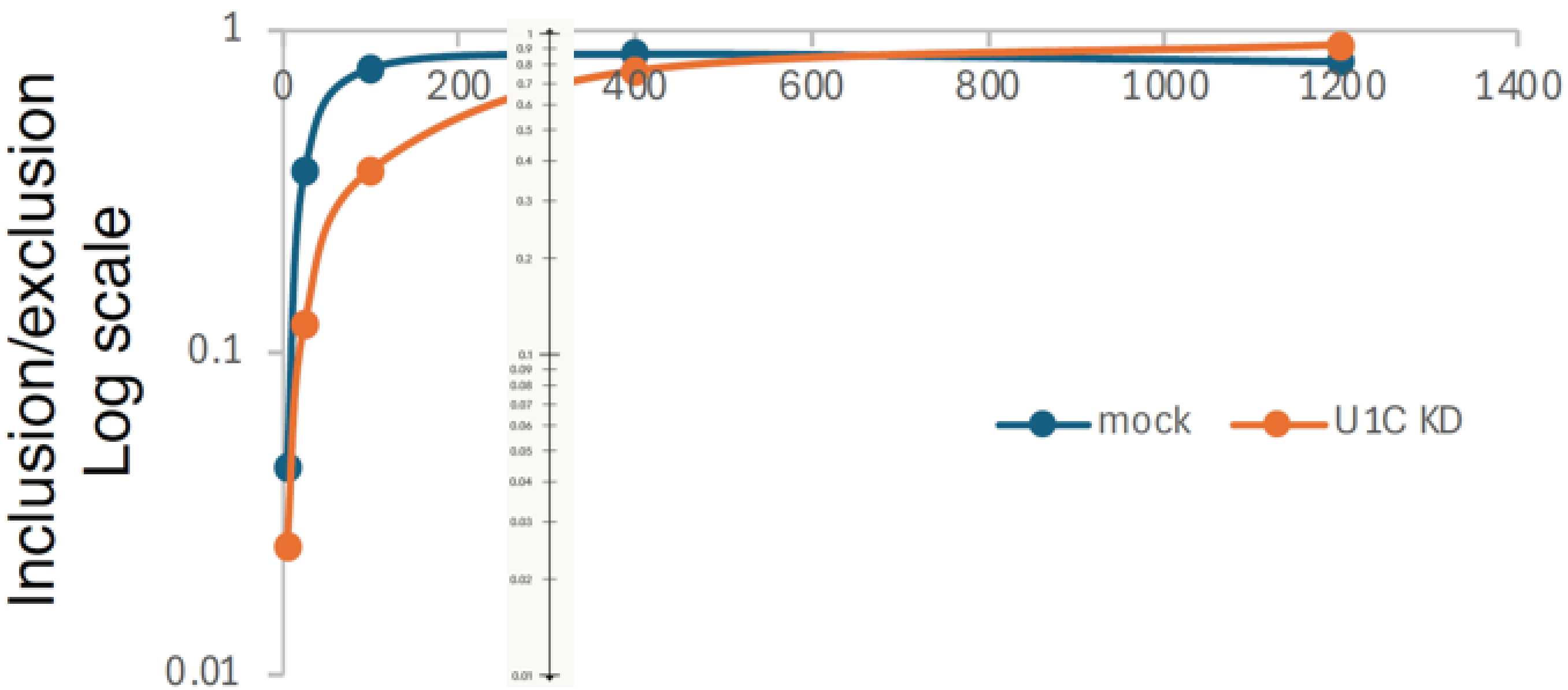

Response Fig. 2 (bottom-right)
with log-scale axis of smaller ticks

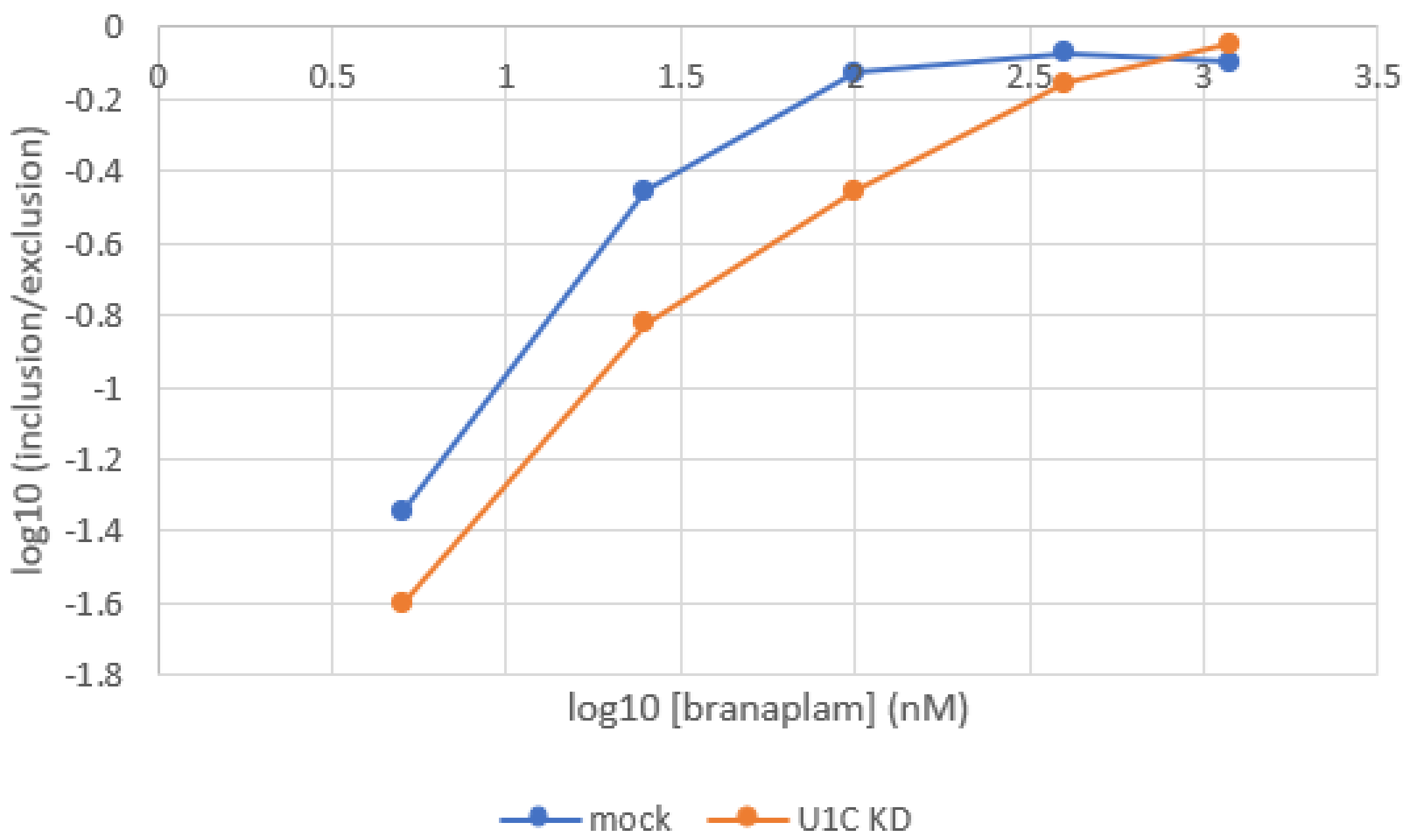

[drug]	mock	U1C KD		log[drug]	mock	U1C KD
5	0.045	0.025		0.69897	-1.34679	-1.60206
25	0.35	0.15		1.39794	-0.45593	-0.82391
100	0.75	0.35		2	-0.12494	-0.45593
400	0.85	0.7		2.60206	-0.07058	-0.1549
1200	0.8	0.9		3.079181	-0.09691	-0.04576

Equation 1:

$$p_i = \frac{e^{-G_i/RT}}{Z}, \qquad Z = \sum_j e^{-G_j/RT}$$

Equation 2:

$$E \equiv \frac{\text{total weight of all U1-bound states}}{\text{weight of U1-bound, drug-free state}} = \frac{S + S \cdot e^{-\Delta G_{\text{drug}}/RT}}{S} = 1 + e^{-\Delta G_{\text{drug}}/RT}$$

Equation 3:

$$W_{U1} = S + S \cdot e^{-\Delta G_{\text{drug}}/RT} = S \cdot \left(1 + e^{-\Delta G_{\text{drug}}/RT}\right) = S \cdot E$$

Equation 4:

$$Z = \underbrace{1}_{\text{state 1}} + \underbrace{S}_{\text{state 2}} + \underbrace{S \cdot e^{-\Delta G_{\text{drug}}/RT}}_{\text{state 3}} = 1 + S \cdot E$$
