# Supplementary material for: The U1 snRNP protein U1C and Helix H of U1 snRNA are critical for small molecule splicing modulator function
Source: Nat Commun. 2026 Jul 16;17:8837. doi: 10.1038/s41467-026-74765-8 (PMC13500670; doi:10.1038/s41467-026-74765-8)
Supplement: Supplementary file 4 [file 41467_2026_74765_MOESM2_ESM.docx]

Description of Additional Supplementary Files

File name: Supplementary Data 1

Description: Splicing analysis table of RNA-Seq of U1-GA variants with or without U1 Helix H mutations.

Column names: “coordinates”, the boundary of the exon or exonic region in human genome (hg19); “gene_id”, the NCBI gene ID; “refseqid”, Refseq transcript ID; “reg_len”, length (bp) of the exon or exonic region; “startSS_seq” and “endSS_seq”, sequences of start splice site and the end splice site positions (lowercase for intronic sequence and uppercase for exonic sequence). “PSI_SampleName”, Percentspliced-in (PSI) of an exon or region of a sample or average value of a sample group. "deltaPSI_TestSample_Control Sample", difference of average PSI values comparing two sample groups (test vs. control, two sided test); "Pfisher_TestSample_ControlS ample", Fisher's Exact test P value for inclusion and skipping read counts comparing two sample groups (test vs. control, two sided test); "ReguType_TestSample_Contr olSample", region regulation type. (UP, inclusion; DN, skipping; NC, not statistically significant change; na, not available, eg. lowly expressed genes). Helix H mutations Break1, Break2, ID1, ID2 are G12C, C122G, G12A/C122U, G12C/C122G respectively.

File name: Supplementary Data 2

Description: Splicing analysis table of RNA-seq of risdiplam or branaplam treatment in dose response with U1C knockdown or negative control knockdown in HEK293 cells.

Column names: “coordinates”, the boundary of the exon or exonic region in human genome (hg19); “gene_id”, the NCBI gene ID; “refseqid”, Refseq transcript ID; “reg_len”, length (bp) of the exon or exonic region; “startSS_seq” and “endSS_seq”, sequences of start splice site and the end splice site positions (lowercase for intronic sequence and uppercase for exonic sequence). “PSI_SampleName”, Percent-spliced-in (PSI) of an exon or region of a sample or average value of a sample group. "deltaPSI_TestSample_ControlSample", difference of average PSI values comparing two sample groups (test vs. control, two sided test); EC_dPSI30, compound concentration when dPSI achieves 30%; pEC_dPSI30: -log10 transformed EC_dPSI30; "Pfisher_TestSample_ControlSample", Fisher's Exact test *P* value for inclusion and skipping read counts comparing two sample groups (test vs. control, two sided test); "ReguType_TestSample_ControlSample", region regulation type. (UP, inclusion; DN, skipping; NC, not statistically significant change; na, not available, eg. lowly expressed genes).
