# Supplementary material for: The U1 snRNP protein U1C and Helix H of U1 snRNA are critical for small molecule splicing modulator function
Source: Nat Commun. 2026 Jul 16;17:8837. doi: 10.1038/s41467-026-74765-8 (PMC13500670; doi:10.1038/s41467-026-74765-8)
Supplement: Supplementary file 5 [file 41467_2026_74765_MOESM7_ESM.zip › Source data images.pdf]

Supplementary Fig. 7B

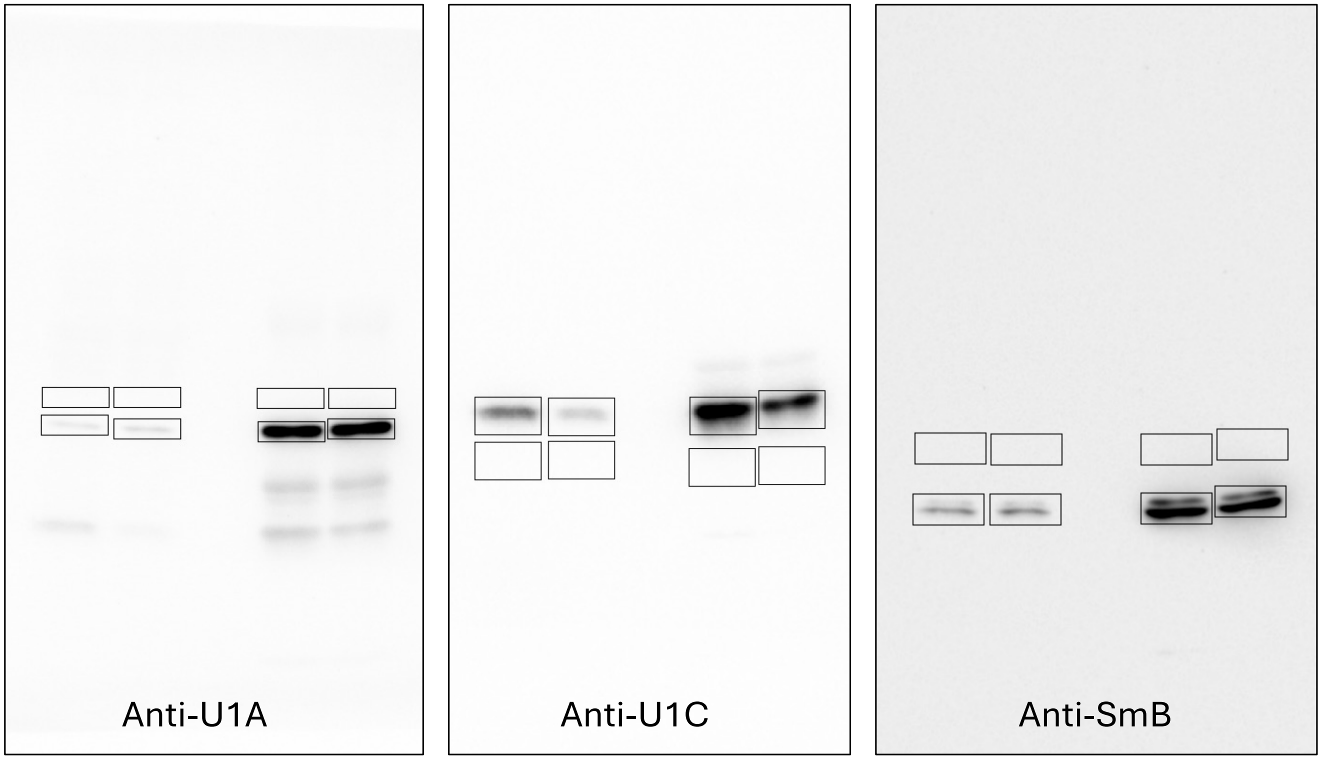

Supplementary Fig. 11C

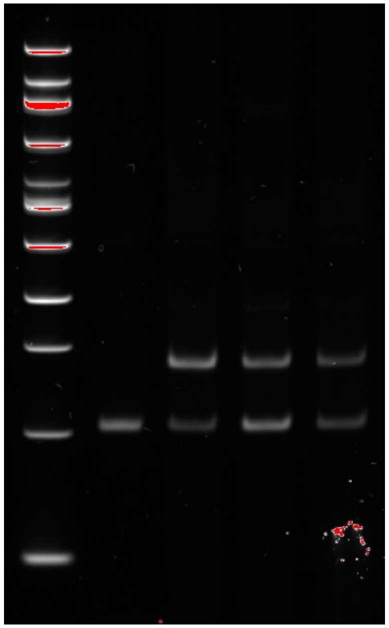

SCO1

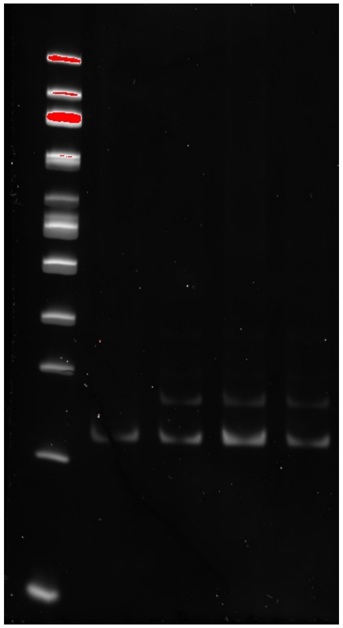

DPH1
